# Assessing the value for money, from a policy maker perspective, of 24 randomised controlled trial designs for an online weight maintenance guided self-help intervention: an expected value of sample information analysis

**DOI:** 10.1038/s41366-025-01804-7

**Published:** 2025-05-22

**Authors:** Penny Breeze, Katharine Pidd, Daniel Pollard, Shijie Ren, Sarah Bates, Chloe Thomas, Amy Ahern, Simon Griffin, Alan Brennan

**Affiliations:** 1 Sheffield Centre of Health and related Research School of Medicine and Population Health, University of Sheffield, Sheffield, United Kingdom; 2 MRC Epidemiology Unit, School of Clinical Medicine, University of Cambridge, Cambridge, United Kingdom

**Keywords:** Weight management, Health policy, Disease prevention

## Abstract

**Objective:**

To analyse whether conducting a randomised controlled trial (RCT) to evaluate an online weight maintenance guided self-help intervention (Supporting Weight Management (SWiM)) would offer good value for money in the United Kingdom.

**Method:**

We examined 24 RCT designs by varying inclusion criteria (participants completing behavioural weight management, specialist-led weight management, diabetes prevention programme, type 2 diabetes remission, digital weight management, all weight management services), trial duration (1–2 years), and sample size (*n* = 500 or 2000). Trial benefits were estimated by the method of expected value of sample information analysis using a health economic model. The model examines how the proposed intervention affects weight maintenance over time (with uncertainty), and generates estimated lifetime Quality Adjusted Life Years (QALYs) and National Health Service (NHS) costs. Structured expert elicitation with 4 experts was undertaken to quantify pre-trial uncertainty in the effectiveness of SWiM compared with usual care. All trial designs were simulated to estimate trial benefits: the reduction in the costs of an inefficient decision for future populations over 10 years. Trial designs offer value for money if trial benefits exceed trial costs.

**Results:**

For three inclusion criteria options (groups recently completing ‘diabetes remission’, ‘digital weight management’ or ‘specialist weight management’), the cost of the proposed trials was estimated to exceed the estimated trial benefit (value of the reduction in decision uncertainty) over 10 years. For the other three inclusion criteria options (groups recently completed ‘behavioural weight management’, ‘diabetes prevention programme’, or ‘all weight loss programmes’), 12 trial designs produced greater benefits than costs. The optimal trial design option would include ‘all weight loss programmes’, with 2 years follow-up and sample size *n* = 2000.

**Conclusion:**

Investment in a large RCT to evaluate the SWiM intervention for patients completing a range of weight loss interventions offers the greatest value to the NHS.

## Background

Economic evaluations of behavioural weight management programmes for individuals with elevated BMI have found that these programmes are cost-effective, and potentially cost-saving in the long-term [1–6]. A meta-analysis of trials for lifestyle, pharmacotherapy, and surgical interventions demonstrated that weight loss >10% significantly reduces the risk of, and management of, type 2 diabetes [7]. In the United Kingdom the National Institute for Health and Care Research (NICE) recommends multicomponent weight management services, addressing dietary intake, physical activity levels and behaviour change, for people with overweight or obesity [8]. Weight loss programmes follow a pattern of substantial initial weight loss followed by some or total weight regain [1, 9]. Publicly funded services for weight maintenance are not available to support patients in the UK. Strategies to promote lasting adherence to changes in diet and increased physical activity are challenging to implement because attendance at behavioural weight loss programmes declines over time [10].

Acceptance-based behavioural interventions have superior long-term weight outcomes compared to standard behavioural programmes [11]. A rigorous process was taken to develop Supporting Weight Management (SWiM), a web-based, guided self-help intervention that uses Acceptance and Commitment Therapy (ACT) [11]. SWiM was designed to be implemented at scale and uses digital technology and non-specialist guides to reduce the resources needed to deliver the intervention. A feasibility study of the SWiM program found that at 6 months, SWiM participants lost an average of 2.15 kg (SD 6.43), while the control group gained an average of 2.17 kg (SD 6.60) [12]. However, a full scale randomised controlled trial (RCT) is needed to generate estimates of effectiveness and cost-utility to inform policy decisions to commission SWiM.

Health economic models estimate lifetime costs, QALYs, and cost-utility to inform national guidelines, extrapolating from short-term intervention data [13]. This method provides a formal process to decide whether a new intervention offers value for money over other options and should be adopted [14]. This decision will always be made with uncertainty and the analysis should communicate this to policymakers [15]. Where decisions are uncertain there is a risk that the analysis will make an incorrect recommendation. It may be preferable to collect more data before implementing the new intervention at scale [16]. Health economic modelling with value of information analysis allows new research to be valued in terms of the anticipated reduction in uncertainty for policy decision-making based on cost-utility estimates [17]. Expected value of sample information (EVSI) is a method that quantifies the reduction in uncertainty for a research designs [17, 18]. Economists can then calculate the expected net benefit of sampling (ENBS), which is the difference between the expected value of the research and the expected research costs. Alternative designs can be compared to identify designs that maximise the ENBS. Taken together these approaches offer research funders a framework to improve research funding allocation and research designs to inform economic evaluations.

We evaluate the value of an RCT to assess whether participants in a structured weight maintenance intervention will experience significantly less weight regain compared with participants in a control arm. This study estimates the EVSI and ENBS of twenty-four RCT designs for SWIM with varying sample size; duration of follow-up and inclusion criteria.

## Methods

The analysis and reporting adhere to the Consolidated Health Economic Evaluation Reporting Standards - Value of Information (CHEERS-VOI) checklist [19].

### Population

Five behavioural weight management services were identified to precede SWiM: (Tier 2 weight management [20]); specialist weight management (Tier 3 weight management [20]); Diabetes prevention programme (NHS Diabetes Prevention Programme [21]); Diabetes remission (NHS Path to remission [22]); and Digital weight management (NHS Digital weight management [23]). A sixth population described an ‘all weight management’ population in which participants were referred from all five services to receive SWIM. Synthetic populations were generated from the adult Health Survey for England 2018 [24] using iterative proportional fitting (IPF) to represent the characteristics of participants completing weight management services commissioned in the UK. IPF methods generate sample weights to align simulated data to the eligible population [25] to reflect differences in baseline risk. Details of the data and methods used are detailed in the supplementary appendix. In each analysis the population entering the model were assumed to have completed a weight management programme and the initial weight loss heterogenous and simulated conditional on the simulated individual’s characteristics. Table 1 reports summary statistics for each population.

**Table 1:** Baseline characteristics for the 50,000 simulated individuals & estimated annual eligible population in England for each of 6 defined population trial inclusion criteria options.

	Population inclusion criteria - People who recently completed					
	Behavioural weight management	Specialist weight management	Diabetes prevention programme	Digital weight management	Diabetes remission	All weight loss programme populations
	Number (%)	Number (%)	Number (%)	Number (%)	Number (%)	Number (%)
Male	12,127 (24%)	14,984 (30%)	22,464 (45%)	16,293 (33%)	23,419 (47%)	16,942 (33.9%)
Female	37,873 (76%)	35,016 (70%)	27,536 (55%)	33,707 (67%)	26,581 (53%)	33,058 (66.1%)
White	43,435	43,687 (87%)	44,413 (89%)	41,810 (84%)	40,054 (80%)	43,028 (86.1%)
Black African/Caribbean	1988	2592 (5%)	1254 (3%)	2591 (5%)	4297 (9%)	2431 (4.9%)
Asian	3178	2880 (6%)	3487 (7%)	4775 (10%)	4701 (9%)	3477 (7.0%)
Other ethnicity	1399	841 (2%)	846 (2%)	824 (2%)	948 (2%)	1064 (2.1%)
Underweight/Healthy (<25 kg/m^2^)	0	0	9120 (18%)	0 (0)	5269 (11%)	2743 (5.5%)
Overweight (25–30)	7721 (15%)	0	17,711 (35%)	697 (1.4%)	8803 (18%)	8321 (16.6%)
Obesity class 1 and 2 (30–40)	30,200 (60%)	16,281 (33%)	23,169 (46%)	33,420 (66.8%)	35,928 (72%)	25,554 (51.1%)
Obesity class 3 (40+)	12,079 (24%)	33,719 (67%)	0	15,883 (31.8%)	0	13,382 (26.8%)
Current smoker	7967 (16%)	7169 (14%)	7789 (16%)	4589 (9%)	6738 (13%)	7575 (15.2%)
Type 2 diabetes	3307(7%)	16,088 (32.2%)	0 (0%)	19,089 (38.2%)	50,000 (100%)	14,541 (29.1%)
Non-diabetic hyperglycaemia	5236 (10.5%)	5634 (11.3%)	50,000 (100%)	25,467 (10.9%)	0 (0%)	13,209 (26.4%)
Normoglycemia	41,457 (82.9%)	28,278 (56.6%)	0 (0%)	5444 (50.9%)	0 (0%)	22,250 (44.5%)
Hypertension	6215 (12%)	11,639 (23%)	13,888 (28%)	36,308 (73%)	16,458 (33%)	11,108 (22.2%)
Statins	4330 (9%)	9157 (18%)	14,730 (29%)	16,612 (33%)	18,114 (36%)	10,135 (20.3%)
	Mean (SD)	Mean (SD)	Mean (SD)	Mean (SD)	Mean (SD)	Mean (SD)
Age	50.4 (15.3)	53.4 (15.9)	64.3 (12.9)	59.5 (13.7)	60.5 (12.4)	55.7 (15.5)
BMI (kg/m^2^)	35.23 (6.44)	41.47 (5.78)	30.11 (5.87)	37.43 (6.29)	33.42 (6.68)	35.15 (7.26)
HbA1c (%)	5.62 (0.72)	6.14 (1.18)	6.12 (0.13)	6.34 (1.24)	7.46 (1.40)	6.21 (1.16)
SBP (mmHg)	126.36 (16.97)	129.98 (18.04)	126.81 (16.09)	131.65 (17.77)	132.62(15.87)	128.49 (16.98)
CHOL (mmol/l)	5.19 (1.01)	5.00 (1.03)	5.14 (1.10)	4.97 (1.01)	4.62 (1.10)	5.04 (1.09)
HDL (mmol/l)	1.40 (0.38)	1.31 (0.36)	1.37 (0.39)	1.38 (0.38)	1.30 (0.38)	1.36 (0.38)
Height (m)	1.65 (0.09)	1.64 (0.10)	1.65 (0.10)	1.64 (0.10)	1.66 (0.10)	1.65 (0.10)
Estimated annual eligible population in England	18 323	5 446	53 000	14 000	960	89 742

*BMI* Body Mass Index following weight management programme, *HDL* High-density lipoprotein, *SBP* systolic blood pressure, *SD* standard deviation.

### SWiM Intervention

**Fig. 1:**
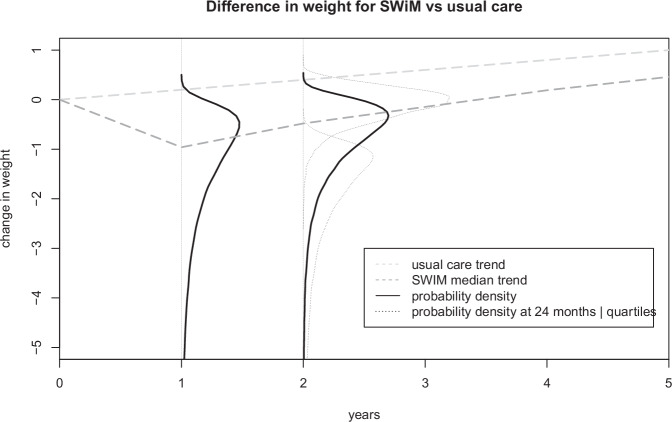
Difference in weight for SWiM vs. usual care. Pre-trial estimation of uncertainty in treatment effect for difference in weight for SWiM versus usual care at 12 months and 24 months based on structured elicitation with four experts. Solid black lines indicate fitted probability distribution for weight difference, with solid grey lines indicating variability of effect given 12 month outcomes. Dashed lines indicate simulated mean trajectory for SWiM and usual care.

The SWIM intervention was developed to support patients who have recently completed a weight management programme to reduce weight regain. The SWIM feasibility study provided an estimate of the adjusted difference between the study groups in weight change from baseline to 6 months was −3.86 kg (95% CI: −7.83 to 0.11 kg, *p* = 0.06) [12]. This data is from a small feasibility study and the effectiveness beyond 6 months is unknown. We consulted with stakeholders with expertise in weight management to characterise weight differences over time. We employed the Sheffield ELicitation Framework (SHELF) methodology to elicit expert opinions on the distribution of uncertainty in intervention effects on weight [26], and followed best practice guidance [27]. The University of Sheffield School of Medicine and Population health research ethics committee granted approval for the elicitation (reference 050092) in accordance with the relevant guidelines and regulations. We obtained informed consent and circulated an evidence dossier to workshop participants based on the SWIM feasibility study (6 month weight outcome) with evidence synthesis of similar interventions (ACT and behavioural weight maintenance interventions) prior to the workshop. The evidence dossier identified that behavioural weight maintenance interventions reduce weight regain, with some evidence that ACT-based interventions are more effective than standard behavioural approaches. The workshop aimed to estimate probability distributions for the difference in weight at 12 and 24 months for SWiM compared with usual care [26]. Previous service and magnitude of weight loss was not assumed to impact the effectiveness of SWIM. The difference in weight at 24 months was elicited conditional on weight difference at 12 months, such that larger treatment effects at 12 months were associated with larger treatment effects at 24 months. Beyond 24 months the intervention effects decline linear until returning to the natural history trajectory after 8 years. The evidence dossier and outcomes of the expert elicitation are reported in Section 11 of the supplementary material. The final parameter distributions for differences in weight are illustrated in Fig. 1.

We assumed that intervention effects on HbA1c would be conditional on weight loss [28]. We used associations between these outcomes for individuals with non-hyperglycaemic, non-diabetic hyperglycaemia and type 2 diabetes derived from statistical analyses of two weight management randomised controlled trials (Supplementary Information: Section 11). The intervention cost for SWIM was estimated to be £221 and no cost was assigned to usual care (Section 12 of the supplementary appendix).

### Health economic analysis

The economic evaluation estimated the cost-utility of SWiM vs. usual care in the United Kingdom followed national guidelines [1, 13]. UK usual care does not offer referral to weight maintenance services, but individuals may pay for services out of pocket. The analysis adopted an NHS and Personal Social Services perspective to estimate the lifetime costs in 2020/21 UK pounds (£). Lifetime Costs and QALYs were discounted at 3.5%. The lifetime discounted costs and QALYs are used to generate incremental cost-utility ratios, and incremental Net Monetary Benefit assuming a willingness to pay threshold of £20,000-per-QALY [13].

### The model

The School for Public Health Research (SPHR) Diabetes prevention model (version 5.2) is an individual patient level microsimulation based on the evolution of personalised trajectories body mass index (BMI), HbA1c and other metabolic risks. The model was selected to capture long-term benefits of the intervention and reflect heterogenous risks of obesity-related complications. Short-term weight trajectories for weight were conditional on age, sex, BMI at baseline and weight dynamics in the previous period [1]. Beyond 5 years metabolic risk trajectories utilise trajectories derived from UK cohort studies [29, 30]. Statistical models from the UKPDS [31, 32], and QResearch algorithms [33, 34] simulated the risk of type 2 diabetes, or diabetes related complications depending on the individual’s characteristics and metabolic health status and assigns costs to these. Health related quality of life decrements were applied to age, weight, and health complications over time. Full details of the model and uncertain parameter inputs are provided in the supplementary appendix. The model can be used to assess the long-term cost-utility of weight loss interventions in the UK by analysing the health impact of reduced weight regain and HbA1c between SWiM and usual care, and cost of the intervention. The code is written in R and available upon request from the authors.

A probabilistic sensitivity analysis (PSA) was generated with 3000 sampled inputs for a population of 50,000 individuals to generate 3000 Incremental Net Monetary Benefit estimates. The stability of the model across individuals and PSA runs are reported in Section 13 of the supplementary appendix.

### Value of information analysis

In the UK health economic models use PSA to describe uncertainty about whether a new intervention is cost-effective compared to a comparator [13, 15]. Model parameter inputs are represented as distributions around the point estimate to capture the uncertainty in the input [35]. This can be used to estimate the likelihood that an intervention meets a well-established willingness to pay threshold for a cost per quality adjusted life year (QALY) gained. The analysis may indicate that the intervention is likely to be cost-effective, but also express a level of uncertainty. New research may reduce the uncertainty in model parameters and reduce the risk of a sub-optimal decision; i.e. a decision to fund an intervention based on an expected cost-utility estimate when the true cost-utility is above the cost-per-QALY threshold of £20,000-per-QALY [16, 36]. This value of information analysis explored how 24 simulated RCT design choices (sample size, follow-up, inclusion criteria) influence the precision of evidence. Previous studies show that trial designs that collect more data increase precision in their estimates and have greater value but often with diminishing returns [17]. The analysis values the reduction in uncertainty provided by the simulated RCT data in terms of the increase in the expected health and economic benefit for a single patient. This is consistent with the output from health economic analyses, but this only describes the benefit at the patient level. The decision-maker should consider the total value to society of the RCT, conditional on how many patients will receive the intervention and over what time horizon [37].

### Simulating the proposed RCT designs

The analysis considered twenty-four trial design options combining two sample size options (500 participants, 2000 participants), two duration of follow-up options (12 months, 24 months) and six inclusion criteria options (Table 2). An RCT is assumed to provide data for SWiM and usual care for up to 4 quantities (1) difference in weight at 12 months, (2) difference in HbA1c at 12 months, (3) difference in weight at 24 months, and (4) difference in HbA1c at 24 months. RCT data was simulated using Microsoft Excel (Version 3202) to predict the four observable quantities. The mean change in weight at 12 months and 24 months were sampled from the prior parameter distribution. Variability in weight was assumed with a standard deviation of 6.4 to be consistent with data from a previous weight management trial (1). The sampled prior information for the relationships between weight and HbA1c by diabetes status was used to estimate the mean change in HbA1c for the population. The standard deviation for HbA1c was 5.95 based on a previous weight loss trial (1). For all study designs we assume that 30% of participants would be lost to follow-up, impacting on the simulated standard error from the trial [38].

**Table 2:** Results from simulation of 3000 possible future trial outcomes: simulated mean difference for SWiM versus Usual Care in Weight and HbA1c across 24 proposed trial designs.

Population inclusion criteria People who recently completed …	Duration of follow-up	Sample size	Trial cost £	12 Months		24 Months	
				Mean Change in Weight (kg) (standard error)	Mean Change in Hba1c (%) (standard error	Mean Change in Weight (kg) (standard error)	Mean Change in Hba1c (units) (standard error
Behavioural weight management	1 year	500	£1,543,500	−1.953 (0.034)	−0.079 (0.006)		
		2000	£2,010,996	−1.957 (0.033)	−0.088 (0.003)		
	2 years	500	£1,999,167	−1.953 (0.034)	−0.079 (0.006)	−1.619 (0.033)	−0.069 (0.006)
		2000	£2,568,667	−1.957 (0.033)	−0.088 (0.003)	−1.606 (0.033)	−0.065 (0.003)
Specialist weight management	1 year	500	£1,543,500	−1.952 (0.033)	−0.132 (0.006)		
		2000	£2,010,996	−1.953 (0.033)	−0.127 (0.004)		
	2 years	500	£1,999,167	−1.952 (0.033)	−0.132 (0.006)	−1.606 (0.033)	−0.109 (0.006)
		2000	£2,568,667	−1.953 (0.033)	−0.127 (0.004)	−1.604 (0.033)	−0.105 (0.004)
Diabetes prevention programme	1 year	500	£1,543,500	−1.953 (0.034)	−0.102 (0.006)		
		2000	£2,010,996	−1.952 (0.033)	−0.106 (0.003)		
	2 years	500	£1,999,167	−1.953 (0.034)	−0.102 (0.006)	−1.610 (0.033)	−0.095 (0.006)
		2000	£2,568,667	−1.952 (0.033)	−0.106 (0.003)	−1.607 (0.033)	−0.083 (0.003)
Digital weight management	1 year	500	£1,543,500	−1.953 (0.033)	−0.147 (0.006)		
		2000	£2,010,996	−1.950 (0.033)	−0.143 (0.004)		
	2 years	500	£1,999,167	−1.953 (0.033)	−0.147 (0.006)	−1.603 (0.033)	−0.117 (0.006)
		2000	£2,568,667	−1.950 (0.033)	−0.143 (0.004)	−1.605 (0.033)	−0.117 (0.004)
Diabetes remission	1 year	500	£1,543,500	−1.954 (0.034)	−0.277 (0.008)		
		2000	£2,010,996	−1.947 (0.033)	−0.272 (0.006)		
	2 years	500	£1,999,167	−1.954 (0.034)	−0.277 (0.008)	−1.604 (0.033)	−0.217 (0.007)
		2000	£2,568,667	−1.947 (0.033)	−0.272 (0.006)	−1.606 (0.033)	−0.217 (0.005)
All weight loss programme populations	1 year	500	£1,543,500	−1.960 (0.034)	−0.131 (0.006)		
		2000	£2,010,996	−1.950 (0.033)	−0.135 (0.004)		
	2 years	500	£1,999,167	−1.960 (0.034)	−0.131 (0.006)	−1.612 (0.033)	−0.104 (0.006)
		2000	£2,568,667	−1.950 (0.033)	−0.135 (0.004)	−1.604 (0.033)	−0.108 (0.004)

Standard deviations used in sampling are calculated based on the sample size, with an assumed total participant loss to follow-up of 30% per trial independent of trial design.

### The EVSI and ENBS analysis

We used an efficient regression-based approximation method, that has been previously tested and evaluated, to generate EVSI estimates [37]. Approximation methods are less computationally demanding processes than traditional methods compatible with computationally intensive models [18]. Following terminology used in Bayesian statistics we refer to distributions before the RCT as the prior. The method required the following steps:

1. We performed a PSA the SPHR diabetes prevention model simulation to obtain a sample of 3000 uncertain parameter inputs and corresponding discounted costs, QALYs. The prior incremental Net Monetary Benefit for each programme population based on the prior parameter distributions, was calculated from the simulated costs and QALYs for each PSA parameter input sample.
2. RCT outcomes for weight and HbA1c differences were simulated for all the proposed trials. For each trial design 3000 trial outcomes were sampled from a normal distribution in which each the mean was equal to prior parameter input sample and estimated standard error based on the trial sample size and standard deviation.
3. We used the Sheffield Accelerated Value of Information tool (SAVI) [39] to apply the efficient regression-based approach to calculate per person EVSI. SAVI is one of four web-based tools available to support value of information analysis, and was chosen due for its efficiency and ease of use by the research team [40]. This describes the extent to which the additional information from the study reduces the expected losses of an inefficient decision. We extracted the values from SAVI to estimate the per-patient EVSI for the SWiM intervention. The EVSI calculation generates a value of information per person who will be affected by the decision.
4. To make this useful for publicly funded research funders in the UK, the population EVSI was calculated for the potential eligible population over the time horizon the intervention will be implemented. This estimates the total value of research from an NHS and personal social services perspective. We estimate the potential eligible population as the estimated annual size of the population completing the weight management service. The per person ESVI was multiplied by the total population for a time horizon of 10 years to be consistent with other EVSI studies and acknowledge that the intervention will be superseded.
5. The Expected Net Benefit of Sampling is calculated by subtracting the estimated cost of the RCT away from the population EVSI.

The number of people who benefit in each of the five programme populations listed above have been estimated [41–43], with full details provided in the supplementary appendix. The size of the sixth all weight management population is the sum of the five individual services, adjusting for overlapping populations (Table 1). We estimate the per-person EVSI for a total of 24 trial designs, combining alternative combinations of sample size, duration of follow-up and inclusion criteria. For each trial design we also report the total EVSI over the eligible population and a discounted 10 year time horizon at 3.5%. We approximate the cost of an RCT including fixed overhead costs for research consumables, staff costs were conditional on the duration of follow-up, and per participant costs conditional on the number of participants recruited. The final costs estimate for the research designs are reported in Table 2, with full details provided in Section 15 of the supplementary appendix.

## Results

The expected incremental cost-utility ratios are less than cost-per-QALY threshold of £20,000 for SWiM compared with usual care across all weight management populations, but varies from £597/QALY gained to £4940/QALY gained. There is uncertainty, the probability that the intervention is cost-effective ranges from 65-80%. SWIM following a digital weight management programme offers the highest expected Net Monetary Benefit at £459 (95% CI £-168 to £2 003) and Diabetes Prevention Programme the lowest at £164 (95% CI 167 to £877), due to variation in average BMI and prevalence of comorbidities. The diabetes remission population estimates lowest incremental costs £10 (95% CI (£-513 to £172) compared with other populations due to the additional cost saving from reduced diabetes medication costs, and higher risk of complications. The prior incremental expected net monetary benefit of the SWIM intervention across all five populations completing alternative weight management programmes and a combined population is reported in Table 3.

**Table 3:** Pre-Trial Uncertainty in Health Economic outcomes based on 3000 probabilistic sensitivity analysis samples of the prior parameter values incorporating the structured elicitation from 4 experts on the uncertainty in effectiveness of SWiM intervention versus usual care.

	Prior mean incremental costs [95% credible interval]	Prior mean incremental QALYS	Prior mean incremental cost per QALY (ICER)	Net monetary benefit (QALY valued at £20,000)	Probability cost-effective at £20,000 per QALY threshold
Behavioural weight management	£94.63 [£−232.90, £195.87]	0.019 [0.000, 0.046]	£4,940.31	£287.37 [£−188.02, £1010.26]	0.76
Specialist weight management	42.14 [£−418.94, £185.74]	0.023 [0.001, 0.062]	£1,852.32	£411.86 [£−171.01, £1603.16]	0.78
Diabetes Prevention Programme	£105.72 [£−141.59, £177.62]	0.013 [0.000, 0.038]	£7,834.89	£164.16 [£−167.37, £877.18]	0.65
Digital weight management	£28.76 [£−447.58, £181.10]	0.024 [0.001, 0.080]	£1,181.05	£459.23 [£−167.99, £2003.37]	0.80
Diabetes remission	£9.87 [£−512.90, £172.19]	0.017 [0.000, 0.050]	£579.00	£332.13 [£−159.71, £1487.70]	0.74
All patients	£71.03 [£−300.54, £183.95]	0.018 [0.001, 0.045]	£3,885.30	£294.97 [£−173.07, £1162.00]	0.75

The simulated difference in weight and HbA1c are reported in Table 2 for each RCT design with an approximate −1.95 reduction in weight at 12 months. Increasing the sample size reduces the uncertainty in measure of weight and HbA1c, as illustrated in the standard errors reported in Table 2. Increasing the duration of follow-up to 24 months provides reduces uncertainty in the maintenance of weight and HbA1c changes across two time-points.

The per-patient EVSI increases with larger sample size and duration of follow-up. A trial in patients completing a diabetes prevention programme is estimated to be the most valuable per patient at £29.39 per person, whereas a trial in patients completing the digital weight loss programme is the least valued per patient at £6.84 per person. The per patient EVSI are similar across each of the proposed trial designs and are reported in column C of Table 4.

**Table 4:** Results comparing expected trial cost with expected value of sample information for 24 possible RCT designs in 6 population subgroup inclusion criteria over a 10 year time horizon in England.

	Population subgroup inclusion criteria – People who have recently completed …	Duration of follow-up	Sample size	Annual eligible population (A)	Estimated RCT cost (B)	Per person EVSI (C)	Total EVSI (D) =(A)*10 years (discounted)*(C)	ENBS (E) = (D) – (B)
1	Behavioural weight management	1 year	500	18,323	£1,543,500	£12.09	£1,906,907	£363,407
2			2000	18,323	£2,010,996	£15.49	£2,442,983	£431,987
3		2 years	500	18,323	£1,999,167	£17.06	£2,690,055	£690,888
4			2000	18,323	£2,568,667	£20.58	£3,246,569	£677,902
5	Specialist weight management	1 year	500	5446	£1,543,500	£7.87	£368,718	−£1,174,782
6			2000	5446	£2,010,996	£11.67	£547,157	−£1,463,839
7		2 years	500	5446	£1,999,167	£12.60	£590,760	−£1,408,407
8			2000	5446	£2,568,667	£16.12	£755,438	−£1,813,229
9	Diabetes prevention programme	1 year	500	53,000	£1,543,500	£22.17	£10,112,973	£8,569,473
10			2000	53,000	£2,010,996	£25.29	£11,538,776	£9,527,780
11		2 years	500	53,000	£1,999,167	£26.53	£12,101,503	£10,102,336
12			2000	53,000	£2,568,667	£29.39	£13,407,835	£10,839,168
13	Digital weight management	1 year	500	14,268	£1,543,500	£6.84	£840,060	−£703,440
14			2000	14,268	£2,010,996	£11.15	£1 369,319	−£641,677
15		2 years	500	14,268	£1,999,167	£11.36	£1 395,366	−£603,801
16			2000	14,268	£2,568,667	£14.16	£1 738,996	−£829,671
17	Diabetes Remission	1 year	500	960	£1,543,500	£11.89	£98,276	−£1,445,224
18			2000	960	£2,010,996	£16.29	£134,636	−£1,876,360
19		2 years	500	960	£1,999,167	£15.96	£131,924	−£1,867,243
20			2000	960	£2,568,667	£20.15	£166,514	−£2,402,153
21	All weight loss programme populations	1 year	500	89,742	£1,543,500	£12.29	£9,492,062	£7,948,562
22			2000	89,742	£2,010,996	£16.27	£12,567,593	£10,556,597
23		2 years	500	89,742	£1,999,167	£17.30	£13,366,236	£11,367,069
24			2000	89,742	£2,568,667	£20.47	£15,809,322	£13,240,655

The all weight loss programme population has the largest population EVSI of £15,809,322 due to the very large potential numbers of people completing weight loss services and eligible to receive the SWIM intervention following the trial. Populations with smaller expected eligible numbers, such as specialist weight management and diabetes remission services report the lowest population EVSI (£368,718 and £98,276 respectively). The population EVSI for all the proposed RCT designs are reported in column D of Table 4.

**Fig. 2:**
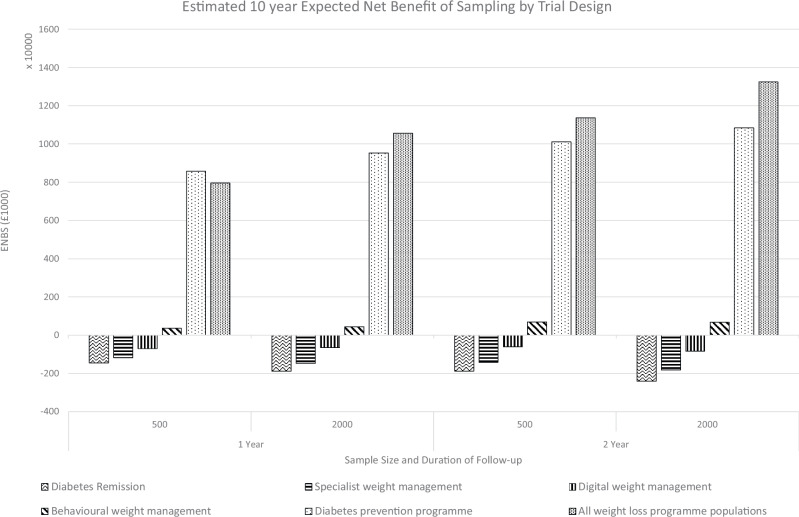
Estimated expected Net benefit of Sampling (ENBS) by trial design.

The estimated RCT cost for each trial design is reported in column B of Table 4. Due to the large overheads in running a trial, increases in sample size and duration of follow-up result in modest increases in the estimate trial cost. After deducting the trial cost, the Expected Net Benefit of Sampling is reported in Column E of Table 4, and illustrated in Fig. 2. Most trials evaluating SWiM generated a negative Expected Net Benefit of Sampling, suggesting that investments in weight loss maintenance research in these populations would not offer good value for money. The trial design generating the largest Expected Net Benefit of Sampling was the large trial with 2 years duration with an inclusion criteria recruiting participants from all weight loss services. This trial design is expected to generate approximately £13 million worth of benefits to the NHS.

## Discussion

This analysis quantifies the uncertainty in the cost-utility of SWiM and demonstrates that investment in further research on intervention effectiveness offers value for money, conditional on trial design. The analysis has found that SWiM could be cost-effective compared with usual care if implemented following five different weight management services. Despite low incremental cost-utility ratios for SWiM, there is uncertainty in these estimates due to the short-term intervention effectiveness that could be updated with RCT evidence, but also from uncertainty in other model parameters not updated in the trial. The risk of making an inefficient commissioning decision for SWiM can be reduced but not eliminated by collecting more evidence. Although the analysis highlights the variation in cost-utility of SWiM across patient populations, the trial design with the greatest ENBS was that which combined all five populations, because this increases the size of the future populations who may benefit from the intervention. A smaller trial of 500 participants in this population was also estimated to generate £8 million of benefits to the NHS.

Predicting the future number of individuals eligible for SWiM is difficult due to limitations in national monitoring, uncertainties in population demographics and service funding. However, given the large difference between the estimated value of research and trial costs the trials would require approximately 15,000 patients per year to use SWiM to justify investment in further research. In the UK, demand for weight management interventions are likely to exceed this estimate [44].

This is the first study to use value of information methods to assess the value of research into weight maintenance interventions. Previously researchers have employed EVSI analyses to estimate sample size estimates for proposed research [45, 46]. Comparisons of per person EVSI estimates are challenging due to differences in study setting and methodological approaches. Nevertheless, a crude comparison with these studies suggests that the maximum value £29 per patient for a SWiM trial design is similar to the maximum £27 and £24 per patient reported in these previous EVSI studies [45, 46] and the duration of follow-up and inclusion criteria were also considered important trial design features. The value to society of the SWIM RCT is similar in magnitude to a previous EVSI analysis. The analysis assessed the value of cost data collection study, which was valued at £11 million [45]. However, despite recent research demonstrating and advocating the use of efficient methods for conducting EVSI analyses there are very few examples within the literature.

It is necessary to make simplifying assumptions in developing a model structure and running health economic analyses. In our simulation weight trajectories are dynamic and personalised, with individual weight at baseline and weight loss influencing subsequent changes. However, weight trajectories may be affected by numerous factors that cannot be captured in the model and annual cycle lengths limit how the rate of weight loss and weight regain within a 1 year period impact health outcomes. Our analysis was limited because it was not possible to elicit from experts the SWIM effect on weight, conditioned on patient characteristics such as baseline BMI, gender, socioeconomic status, comorbidities, initial weight loss, and programme type. Based on existing literature it is uncertain how SWiM intervention effectiveness will vary by initial weight loss or programme type and it was not feasible to explore this in the elicitation exercise. An RCT for an ACT weight maintenance intervention showed greater effects in participants with less initial weight loss, albeit in a very small sample size [47]. In other literature greater initial weight loss was predictive of improved weight maintenance [48], faster weight regain [10], and a review concluded there is insufficient evidence that initial weight loss determines the success of weight loss maintenance [49].

The elicitation exercise required substantial engagement from experts, and was time consuming, particularly because the exercise adheres to guidelines [27]. Our elicitation exercise was conducted within a 4-h workshop, suggesting that each elicited value requires approximately 1–1.5 h. Participants were recruited to capture diverse expertise across different professional and research backgrounds. Due to participant availability, we were only able to recruit 4 participants for this exercise, which is less than suggestions in the literature [50]. This is a limitation of the study and a larger and longer workshop would improve confidence in the distribution and future studies may consider alternative elicitation designs, including consulting experts individually.

It was necessary to use approximation methods to estimate the EVSI in this study. Until recently, EVSI calculations were computationally expensive, because they required nested simulation methods. As such, it would not be possible to generate EVSI estimates for a complicated microsimulation model, as used here. The accuracy and efficiency of approximation methods have been tested and compared and found to generate accurate EVSI estimates [36]. The conditions for this analysis, including the trial design and model simulation time, were consistent with the use of the regression-based method. However, it should be noted that in other studies the PSA samples used to test the approximation methods were larger than the 3000 samples generated for this study. It was not feasible with this model to generate a larger PSA for the EVSI analysis, and stability tests confirmed this was not necessary. The adoption of EVSI approximation techniques has substantially reduced the computational burden of value of information analyses. As such, we have been able to evaluate twenty-four trial designs within this study. Previous studies have highlighted the skills and experience required to implement EVSI analysis as barriers to implementing EVSI methods [37]. Open access tools, such as SAVI used here, increase the opportunity for health economists to adopt these techniques. Therefore, we believe that more widespread use of EVSI is achievable in the future.

## Conclusions

The analysis indicated a strong likelihood of net gains from a new trial. This analysis further suggested that a large-scale trial, encompassing participants recruited from various weight management programs, would be most effective in evaluating the benefits of the SWiM intervention.

## Supplementary information

Supplementary Material

## Data Availability

Most inputs to the model are from published sources and results from the expert elicitation are reported in the supplementary material. The anonymised Health Survey for England datasets can be accessed via the UK Data Service. Researchers interested in accessing the datasets will need to register with UK Data Service to access the data.
