# Supplementary material for: Assessing the value for money, from a policy maker perspective, of 24 randomised controlled trial designs for an online weight maintenance guided self-help intervention: an expected value of sample information analysis
Source: Int J Obes (Lond). 2025 May 22;49(8):1598–607. doi: 10.1038/s41366-025-01804-7 (PMC12396955; doi:10.1038/s41366-025-01804-7)
Supplement: Supplementary file 1 — Supplementary Material [file 41366_2025_1804_MOESM1_ESM.docx]

File S1: Supplementary Methods

# Contents

[1. Contents 1](#_Toc172534912)

[2. Developing the Conceptual Model 5](#_Toc172534913)

[3. Model Structure 6](#_Toc172534914)

[4. Baseline Population Characteristics: Health Survey for England 11](#_Toc172534915)

[Exclusion Criteria 11](#_Toc172534916)

[Re-weighting HSE 2018 data to trial population characteristics 13](#_Toc172534917)

[HSE 2018 Missing data imputation 16](#_Toc172534918)

[5. GP Attendance in the General Population 18](#_Toc172534919)

[6. Longitudinal Trajectories of Metabolic Risk Factors 21](#_Toc172534920)

[WRAP trial Data 22](#_Toc172534921)

[Whitehall II Data Analysis 24](#_Toc172534922)

[ELSA Data Analysis 28](#_Toc172534923)

[BMI Trajectory 29](#_Toc172534924)

[Glycaemic Trajectory in Non-Diabetics 29](#_Toc172534925)

[HbA1c trajectory in type 2 diagnosed diabetics 30](#_Toc172534926)

[Total Cholesterol and HDL Cholesterol Trajectories in Individuals not receiving Statins 31](#_Toc172534927)

[Total Cholesterol and HDL Cholesterol Trajectories in Individuals receiving Statins 32](#_Toc172534928)

[SBP Trajectories in Individuals Not receiving Anti-hypertensive treatment 33](#_Toc172534929)

[SBP Trajectories in Individuals receiving anti-hypertensive treatment 33](#_Toc172534930)

[Metabolic Risk factor screening 34](#_Toc172534931)

[Diagnosis and Treatment Initiation 34](#_Toc172534932)

[Diabetes Diagnosis 35](#_Toc172534933)

[7. Comorbid Outcomes and Mortality 39](#_Toc172534934)

[Cardiovascular Disease 39](#_Toc172534935)

[First Cardiovascular event 39](#_Toc172534936)

[First Cardiovascular event calibration 40](#_Toc172534937)

[First Cardiovascular event inputs 41](#_Toc172534938)

[Subsequent Cardiovascular events 45](#_Toc172534939)

[Congestive Heart Failure 47](#_Toc172534940)

[Microvascular Complications 49](#_Toc172534941)

[Cancer 53](#_Toc172534942)

[Breast cancer 54](#_Toc172534943)

[Colorectal cancer 54](#_Toc172534944)

[Osteoarthritis 55](#_Toc172534945)

[Depression 56](#_Toc172534946)

[Dementia 58](#_Toc172534947)

[Dementia Diagnosis 60](#_Toc172534948)

[Disease Progression 61](#_Toc172534949)

[Mortality 62](#_Toc172534950)

[Cardiovascular Mortality 62](#_Toc172534951)

[Cancer Mortality 62](#_Toc172534952)

[Other cause Mortality (including diabetes and Dementia risk) 62](#_Toc172534953)

[8. Direct Health Care Costs 65](#_Toc172534954)

[GP attendance 66](#_Toc172534955)

[Diabetes 66](#_Toc172534956)

[Metformin Monotherapy 67](#_Toc172534957)

[Metformin plus Gliptins 68](#_Toc172534958)

[Insulin plus Oral Anti-diabetics 69](#_Toc172534959)

[Statins 70](#_Toc172534960)

[Anti-hypertensives 70](#_Toc172534961)

[Cardiovascular Events 71](#_Toc172534962)

[Renal Failure 73](#_Toc172534963)

[Foot Ulcers 73](#_Toc172534964)

[Amputation 73](#_Toc172534965)

[Blindness 74](#_Toc172534966)

[Cancer 74](#_Toc172534967)

[Osteoarthritis 76](#_Toc172534968)

[Depression 76](#_Toc172534969)

[Dementia 78](#_Toc172534970)

[Cost of Diagnosis 78](#_Toc172534971)

[Ongoing healthcare costs 78](#_Toc172534972)

[9. Social Care costs 78](#_Toc172534973)

[Stroke 79](#_Toc172534974)

[Dementia 79](#_Toc172534975)

[10. Utilities 80](#_Toc172534976)

[Baseline Utility 80](#_Toc172534977)

[Utility Decrements 80](#_Toc172534978)

[11. Intervention Effectiveness 85](#_Toc172534979)

[Intervention Effectiveness 85](#_Toc172534980)

[Elicitation Report 85](#_Toc172534981)

[Duration of Intervention Effect 91](#_Toc172534982)

[12. Intervention Costs 91](#_Toc172534983)

[13. Probabilistic Sensitivity Analysis 92](#_Toc172534984)

[14. Model Validation 93](#_Toc172534985)

[15. Expected Value of Sample Information data 95](#_Toc172534986)

[References 100](#_Toc172534987)

# Developing the Conceptual Model

The conceptual model was developed according to a new conceptual modeling framework for complex public health models (1). In line with this framework the conceptual model was developed in collaboration with a project stakeholder group comprising health economists, public health specialists, research collaborators from other SPHR groups, diabetologists, local commissioners and lay members. The initial broad scope for the conceptual model was based on the structure of previous diabetes prevention models used for National Institute for Health and Care Excellence public health guidance (2, 3) and discussions with experts in diabetes prevention modeling. The model was further extended to include Dementia as a possible health outcome for individuals aged over 60 years in the model.

# Model Structure

We developed an individual patient simulation that estimates individuals’ health in yearly cycles until death. The simulation draws baseline demographic and clinical status for individuals sampled from the Health Survey for England (HSE) 2018 (4). The simulation estimates yearly changes in metabolic risk factors based upon the individuals’ baseline characteristics. Within each annual cycle the individuals may be screened for hypertension, dyslipidaemia or diabetes during a visit to the General Practitioner (GP). Opportunistic screening is used to determine diabetes diagnosis or the initiation of anti-hypertensive treatment or statins. Baseline characteristics and metabolic risk factors determine the individuals’ probability of cardiovascular events, diabetes microvascular complications, cancer, osteoarthritis and depression. Individuals within the model may die in any cycle as a result of cardiovascular disease, cancer or from other causes.

Figure S1 illustrates the sequence of updating clinical characteristics and clinical events that are estimated within a cycle of the model. This sequence is repeated for every annual cycle of the model.

The first stage of the sequence updates the age of the individual. The second stage estimates how many times the individual attends the GP. The third stage estimates the change in BMI of the individual from the previous period. In the fourth stage, if the individual has not been diagnosed as diabetic (Diabetes_Dx=0) their change in glycaemia is estimated using the Whitehall II model. If they are diabetic (Diabetes_Dx=1), it is estimated using the UKPDS model. In stages five and six the individual’s blood pressure and cholesterol are updated using the Whitehall II model if the individual is not identified as hypertensive or receiving statins. In stage seven, the individual may undergo assessment for diabetes, hypertension and dyslipidaemia during a GP consultation. From stage eight onwards the individual may experience cardiovascular outcomes, diabetes related complications, cancer, osteoarthritis, depression, dementia, and updated cognitive decline associated with dementia diagnosis. If the individual has a history of cardiovascular disease (CVD history=1), they follow a different pathway in stage eight to those without a history of cardiovascular disease (CVD history=0). Individuals with HbA1c greater than 48 mmol/mol (6.5%) are assumed to be at risk of diabetes related complications. Individuals who do not have a history of cancer (Cancer history=0) are at risk of cancer diagnosis, whereas those with a diagnosis of cancer (Cancer history=1) are at risk of mortality due to cancer. Individuals without a history of osteoarthritis or depression may develop these conditions in stages 12 and 13. In stage 14 individuals with dementia have their cognitive status updated and those individuals aged over 60 without a diagnosis of dementia may receive a diagnosis. Finally, all individuals are at risk of dying due non cardiovascular or cancer mortality. Death from renal disease is included in the estimate of other-cause mortality.

The modeling structure and cycle sequence is explained in more detail using a hypothetical patient below:

Consider a white male aged 53 sampled from the baseline population, referred to hereafter as Mr X. Mr X has a series of baseline demographics informed by the baseline population dataset, or imputation if missing. These characteristics influence his future health outcomes in the model. In the first cycle of the model the age of Mr X is 53. In this cycle Mr X’s attendance at the GP is generated and recorded within the model dependent upon his age and gender. In the first cycle of the model Mr X’s BMI is extracted from his baseline data. The effect of an intervention on BMI in the first 12 months is applied here if required. Similarly baseline values for HbA1c, systolic blood pressure (SBP), total cholesterol, and HDL cholesterol are extracted from his baseline dataset and modified for treatment effect if necessary. If Mr X has attended the GP in this cycle he may receive opportunistic screening for diabetes, hypertension or high cardiovascular risk if he meets certain risk criteria, agreed by the stakeholder group. If he is diagnosed with any of these conditions/risks, treatments are initiated according to current guidelines in the UK for diabetes diagnosis, anti-hypertensive treatment and statin treatment. If Mr X receives any of these treatments his HbA1c, SBP and/or total cholesterol are reduced accordingly.

Having established Mr X’s metabolic risk profile the model determines if Mr X experiences any major health events in this first cycle. If Mr X does not have a history of cardiovascular disease (CVD) the model estimates the probability that he has a fatal or non-fatal cardiovascular event in this cycle. The event is determined using a Bernoulli trial. If Mr X has a history of CVD his probability of a progressing to unstable angina, MI, stroke or a fatal event is determined. If Mr X has HbA1c greater than 48 mmol/mol (6.5%) or a diagnosis of diabetes, the probability of foot ulcer, renal disease, amputation and blindness are calculated and evaluated using a Bernoulli trial. If Mr X does not have diabetes he is not at risk of these complications in this cycle.

In the next stage of the cycle Mr X may develop breast or colon cancer if he has not already got a history of cancer. The probability of these complications is generated and evaluated in a Bernoulli trial. If Mr X has a history of cancer, he is at risk of mortality due to cancer in this cycle. If Mr X does not have osteoarthritis the probability of developing this complication is evaluated in this cycle and a diagnosis is given according to a Bernoulli trial. If Mr X has a diagnosis of osteoarthritis his health status for this complication remains unchanged. Similarly, if Mr X does not have depression the probability of developing it is evaluated in this cycle and a diagnosis is given according to a Bernoulli trial. If Mr X has a diagnosis of depression his health status for this complication remains unchanged. If Mr X is over 60 years old and does not have a diagnosis of dementia, he may receive a new diagnosis according to a Bernoulli trial using his current probability of a dementia diagnosis. If Mr X has an existing diagnosis of dementia his MMSE score will be updated to reflect any deterioration in memory and disease severity.

Finally, assuming Mr X has not experienced a fatal event due to CVD or cancer, the probability of death is calculated and evaluated in a Bernoulli trial based on Office of National Statistics life tables combined with hazard ratios for dementia and diagnosis (5). If Mr X remains alive he proceeds to the next cycle. If Mr X dies his health status, costs and QALYs are stored and he is removed from the model.

In the second and subsequent cycles, the model proceeds through a similar sequence of events. However, Mr X firstly ages by the cycle length of one year. A new number of GP visits within the cycle is generated. BMI will increase or decrease according to a trajectory assigned to Mr X at baseline, and intervention effect maintenance if relevant. Similarly, HbA1c, SBP, total cholesterol, and HDL cholesterol all change in this period on a prespecified trajectory and intervention effect. Mr X may undergo opportunistic screening as specified in year one. The sequence of evaluations to determine health events and complications experienced by Mr X in this cycle is the same as described above, however Mr X’s metabolic risk factors, treatments, and history are updated with the changes described above.

Figure S1: Model Schematic

2. GP visits

.

3.

BMI

.

4.a. Glucose

.

Diabetes_Dx

=1

Diabetes_Dx

=0

1

. Age

4.a. HbA1c+treatment

.

5.a. Blood pressure

.

5.b. Blood pressure

.

Hypertenson

=0

Hypertenson

=1

6.a. Cholesterol

6.a. Cholesterol

Statin=0

Statin=1

7. Screening

.

8.a. CVD events

8.b. CVD events

CVD history=0

CVD history=1

9. CVD

Mortality

10. Renal failure, ulcer,

amputation and blind

HbA

<6.5

HBA>6.5

11.a. Cancer events

11.b. Cancer events

Cancer history=0

Cancer history=1

11.b. Cancer

Mortality

Osteo

history=1

12.

Osteo

events

Osteo

history=0

13. Depression

Depression=0

Depression=1

15. All cause

mortality

Dementia=0

Dementia=1

14.b Update MMSE

14.a Dementia

# Baseline Population Characteristics: Health Survey for England

The model required demographic, anthropometric and metabolic characteristics that would be representative of the UK general population. The Heath Survey for England (HSE) was suggested by the stakeholder group because it collects up-to-date cross-sectional data on the characteristics of all ages of the English population. It also benefits from being a reasonably good representation of the socioeconomic profile of England. A major advantage of this dataset is that includes important clinical risk factors such as HbA1c, SBP, and cholesterol. The characteristics of individuals included in the cost-effectiveness model were based sampled from the HSE 2018 dataset (4). The whole dataset was obtained from the UK Data Service.

## Exclusion Criteria

The total sample size of the HSE 2018 was 10,250. Individuals who were younger than 16 years (N=2072) were excluded from the sample. This left a final sample size of 8,178 individuals. Summary statistics for the data extracted from the HSE 2018 dataset are reported in Table S1 and Table S2.

Table S1: Characteristics of final sample from HSE 2018 (N=8,178)

| Variable name (description) | Mean | Median | SD | Missing (N) |
| --- | --- | --- | --- | --- |
| Age | 50.928 | 51.00 | 18.870 | 0 |
| Weight | 78.64 | 76.50 | 18.338 | 1307 |
| Height | 167.7 | 167.5 | 9.674 | 1286 |
| BMI | 27.77 | 26.94 | 5.706 | 1472 |
| Total Cholesterol | 5.048 | 5.0 | 1.064 | 4628 |
| HDL Cholesterol | 1.495 | 1.4 | 0.425 | 4628 |
| HbA1c | 5.646 | 5.5 | 0.779 | 4681 |
| SBP | 125.4 | 124.0 | 16.728 | 4112 |
| DBP | 72.76 | 72.0 | 10.769 | 4112 |
| EQ-5D | 0.8715 | 0.9370 | 0.194 | 934 |

Table S2: Summary data for categorical (N=8,178)

| Variable name (description) | Category | N | % |
| --- | --- | --- | --- |
| Sex | Total Sample over 16 | 8178 | 100 |
|  | Male | 3669 | 44.86% |
|  | Female | 4509 | 55.14% |
| Age | Age 16-19 | 340 | 4.6% |
|  | Age 20-24 | 408 | 4.9% |
|  | Age 25-29 | 502 | 6.1% |
|  | Age 30-34 | 652 | 8.0% |
|  | Age 35-39 | 661 | 8.1% |
|  | Age 40-44 | 633 | 7.7% |
|  | Age 45-49 | 681 | 8.3% |
|  | Age 50-54 | 705 | 8.6% |
|  | Age 55-59 | 710 | 8.7% |
|  | Age 60-64 | 632 | 7.7% |
|  | Age 65-69 | 688 | 8.4% |
|  | Age 70-74 | 637 | 7.8% |
|  | Age 75-79 | 418 | 5.1% |
|  | Age 80-84 | 276 | 3.4% |
|  | Age 85-89 | 164 | 2.0% |
|  | Age 90+ | 71 | 0.8% |
| Ethnicity | White | 7088 | 86.7% |
|  | Black | 256 | 3.1% |
|  | Asian | 594 | 7.3% |
|  | Mixed/multiple | 136 | 1.7% |
|  | Any other | 78 | 1.0% |
| IMD | Least deprived IMD1 | 1537 | 18.8% |
|  | IMD2 | 1728 | 21.1% |
|  | IMD3 | 1708 | 20.9% |
|  | IMD4 | 1696 | 20.7% |
|  | Most deprived IMD5 | 1509 | 18.5% |
| Hypertensive treatment | Hypertensive medication | 939 | 11.5% |
| Past Cardiovascular disease | History of CVD | 211 | 2.6% |
| Diabetes | History of diabetes | 701 | 8.6% |
| Statins | Statins | 863 | 10.6% |
| Smoking Status | Current smoker | 1316 | 16.1% |
|  | Ex-smoker | 2243 | 27.4% |
|  | Never smoker | 4544 | 55.6% |
|  | Less than 10 cigarettes a day | 573 | 7.0% |
|  | 10-20 a day | 500 | 6.1% |
|  | 20 or more a day | 241 | 2.9% |
|  | Don’t know | 2 | 0.0002% |
| Other health conditions | Non-smoker | 6790 | 83.0% |
|  | Gestational diabetes | 58 | 0.8% |
|  | Mental Disorder | 752 | 9.2% |
|  | Atrial Fibrillation | 400 | 4.8% |
|  | Depression | 1177 | 14.4% |
|  | Arthritis | 839 | 10.3% |

A complete dataset was required for all individuals at baseline. However, no measurements for Fasting Plasma Glucose (FPG) or 2 hour glucose were obtained for the HSE 2018 cohort. In addition, the questionnaire did not collect information about individual family history of diabetes or family history of CVD. These variables were imputed from the Whitehall II dataset (see below) (6).

Many individuals were lacking responses to some questions but had data for others. One way of dealing with this was to exclude all individuals with incomplete data from the sample. However, this would have reduced the sample size and representativeness dramatically, which would have been detrimental to the analysis. It was decided that it would be better to make use of all the data available to represent a broad range of individuals within the UK population. With this in mind, we decided to use assumptions and imputation models to estimate missing data.

## Re-weighting HSE 2018 data to trial population characteristics

Sample weights were generated for the Health Survey for England dataset so that the populations used in the model were representative of the English general population who would be eligible for a group-based weight maintenance intervention using iterative proportional fittings methods (7). There were five populations sampled which represented those eligible for the weight mainentance program, each representative of the population leaving different NHS weight management pathways. The five populations represent individuals leaving a Tier 2 Weight management program, a tier 3 weight management program, a diabetes prevention program, a diabetes remission program and a digital tier 2 weight management program. A sixth population was formed from equal parts of the 5 other populations so generate a population of individuals that could come of any of these five pathways to weight maintenance. The re-weighted Health Survey for England was sampled with replacement to get a simulation population of 50,000 for each subgroup.

We generate sample weights to reflect age, sex, BMI and diabetes status characteristics of these different populations. The Tier 2 Weight management program population was generated using the number of program completers between April 2021 to march 2022 by demographic characteristics in government published data tables (8) to generate weights according to gender, age, BMI classification and diabetes status, excluding individuals with BMI less than 25kg/m^2^. To estimate the number of completers by BMI classification, the number of individuals enrolled by BMI classification was multipled by the proportion of individuals that completed the program by classification. Only those who had a BMI>25kg/m^2^ were included as this is the inclusion criteria to take part in a Tier 2 weight management program. The population representing tier 3 weight management program leavers was weighted by gender, BMI classification and diabetes status based on baseline characteristic from an evaluation (9). The population simulating diabetes prevention program leavers used the baseline statistics of the attenders in a cohort study to generate weights by gender, age, and BMI classification (10). The population of diabetes remission program leavers was simulated using trial data to weight the HSE population based on gender, age and BMI classification (11). To simulation the population leaving a digital Tier 2 weight management program, the Tier 2 population was oversampled and those not meeting the additional exclusion criteras of this program were removed to generate a population of 50,000. The inclusion criteras include having a BMI >30kg/m^2^ or having a BMI >27.5kg/m^2^ for individuals from black, asian and ethnic minority backgrounds and also being diagnosed with type 2 diabetes and/or hypertension. After simulation, the populations HbA1c was compared to the source to check consistency. The summary statistics and simulated HSE population for each subgroup are reported in Table S3 and Table S4. Missing data were imputed using the techniques described below.

Table S3: Summary statistics for the simulation populations and the data sources

| **Characteristic** | **Unit** | **Health Survey for England (2018)** | **Tier 2 Weight Management data (8)** | **Sampled re-weighted Tier 2 population** | **Sampled re-weighted Digital Tier 2 population** | **Tier 3 Weight Management data (9)** | **Sampled re-weighted Tier 3 population** |
| --- | --- | --- | --- | --- | --- | --- | --- |
|  | N | 8178 | 22,415 | 50,000 | 50,000 | 230 | 50,000 |
| Female | N (%) | 4489 (54%) | 15,720 (74%) | 37873 (76%) | 33707 (67%) | 161 (70%) | 35016 (70%) |
| Male | N (%) |  | 5,450 (26%) | 12127 (24%) | 16293 (33%) | 69 (30%) | 14984 (30% |
| Diabetes | N (%) | 548 (6.7%) | 190 (15.6%) | 3307 (15.9%) | 19089 (38.2%) | 73 (32%) | 16088 (32%) |
| Age | Mean (sd) | 50.61 (17.81) |  | 50.38 (15.28) |  | 52.7 (13.6) | 53.41 (15.87) |
| BMI (kg/m^2^) | Mean (sd) | 27.52 (5.44) | 34.54 (5.13) | 35.23 (6.44) | 37.43 (6.29) |  | 41.47 (5.78) |
| Underweight/Healthy (<25kg/m^2^) | N (%) | 0 | 0 | 0 | 0 | 0 | 0 |
| Overweight (>=25 & <30 kg/m^2^) | N (%) | 862 (31%) | 2081 (15%) | 7721 (15%) | 697 (1%) | 0 | 0 |
| Obese I (>=30 & <35 kg/m^2^) | N (%) | 1241 (45%) | 12733 (61%) | 30200 (60%) | 33420 (67%) | 29 (13%) | 6269 (13%) |
| Obese II (>=35 & <40 kg/m^2^) | N (%) | 458 (16%) |  |  |  | 46 (20%) | 10012 (20% |
| Morbidly Obese (>=40 kg/m^2^) | N (%) | 224 (8%) | Est.4985 (24%) | 12079 (24%) | 15883 (32%) | 155 (67%) | 33219 (67%) |
| SD = Standard deviation, IQR = Interquartile range | | | | | | | |

Table S4: Summary statistics for the simulation populationsand the data sources

| **Characteristic** | **Unit** | **Health Survey for England** | **Diabetes Prevention Program data (10)** | **Sampled re-weighted DPP population HSE 2018** | **Diabetes Remission Program data (11)** | **Sampled re-weighted DRP population HSE 2018** |
| --- | --- | --- | --- | --- | --- | --- |
|  | N | 8178 | 55275 | 50,000 | 577 | 50,000 |
| Female | N (%) | 4489 (54%) | 30404 (55%) | 27536 (55%) | 301 (52%) | 26581 (53%) |
| Male | N (%) |  | 24577 (45%) | 22464 (45%) | 276 (48%) | 23419 (47%) |
| Diabetes | N (%) | 548 (6.7%) | 0 (0%) | 0 (0%) | 100% | 100% |
| Age |  | Mean (sd) 50.61 (17.81) | Median (IQR) 66 (17) | Mean (sd) 64.25 (12.86) | Mean (sd) 59.8 (12.6) | Mean (sd) 60.52 (12.54) |
| BMI (kg/m^2^) | Mean (sd) | 27.52 (5.44) |  | 30.11 (5.87) |  | 33.42 (6.68) |
| Underweight/Healthy (<25kg/m^2^) | N (%) | 0 | 7665 (17%) | 9120 (18%) | 9 (2%) | 5269 (11%) |
| Overweight (>=25 & <30 kg/m^2^) | N (%) | 862 (31%) | 16742 (37%) | 17711 (35%) | 140 (24%) | 8803 (18%) |
| Obese I (>=30 & <35 kg/m^2^) | N (%) | 1241 (45%) | 21253 (47%) | 23169 (46%) | 426 (74%) | 35928 (72%) |
| Obese II (>=35 & <40 kg/m^2^) | N (%) | 458 (16%) |  |  |  |  |
| Morbidly Obese (>=40 kg/m^2^) | N (%) | 224 (8%) |  |  |  |  |
| SD = Standard deviation, IQR = Interquartile range | | | | | | |

## HSE 2018 Missing data imputation

#### Ethnicity

Only a small number of individuals had missing data for ethnicity. In the QRISK2 algorithm the indicator for white included individuals for whom ethnicity is not recorded. In order to be consistent with the QRISK2 algorithm we assumed that individuals with missing ethnicity data were white.

#### Anthropometric data

Data were imputed using multiple imputation methods from the mice package in R. Predictive mean methods imputation methods were used to impute missing data based on age, socioeconomic status, anti-hypertensive treatment, diabetes diagnosis, anthropometric, metabolic data and EQ-5D within the dataset. Limiting the number of relationships to include in the imputation method was necessary to reduce the computational time required by the imputation.

#### Metabolic data

Data were imputed using multiple imputation methods from the mice package in R. Predictive mean methods imputation methods were used to impute missing data based on age, socioeconomic status, anti-hypertensive treatment, diabetes diagnosis, anthropometric, metabolic data and EQ-5D within the dataset. Limiting the number of relationships to include in the imputation method was necessary to reduce the computational time required by the imputation.

#### Treatment for Hypertension and Statins

A large proportion of individuals had missing data for questions relating to whether they received treatment for hypertension or high cholesterol. The majority of non-responses to these questions were coded to suggest that the question was not applicable to the individual. As a consequence it was assumed that individuals with missing treatment data were not taking these medications.

#### Anxiety/Depression

Most individuals who had missing data for anxiety and depression did so because the question was not applicable. A small sample N=69 refused to answer the question. We assumed that individuals with missing data for anxiety and depression did not have severe anxiety/depression.

#### Smoking

Individuals with missing data for smoking status were assumed to be non-smokers, without a history of smoking.

#### Rheumatoid Arthritis

Indiivduals reporting existing arthritis/rheumatism were assigned to a history rheumatoid arthritis.

Atrial Fibrillation

Individuals reporting “other heart conditions” in response to questions about long-standing illnesses were assumed to have a history of Atrial Fibrillation.

#### Family history of diabetes

No questions in the HSE referred to the individual having a family history of diabetes, so this data had to be imputed. It was important that data was correlated with other risk factors for diabetes, such as HbA1c and ethnicity. We analysed a cross-section of the Whitehall II dataset to generate a logistic regression to describe the probability that an individual has a history of diabetes conditional on their HbA1c and ethnic origin. The model is described in Table S5.

**Table S5: Imputation model for history of diabetes**

|  | Coefficient | Standard error |
| --- | --- | --- |
| Intercept | -3.29077 | 0.4430 |
| HbA1c | 0.28960 | 0.0840 |
| HDL Cholesterol | 0.81940 | 0.1388 |

#### History of Cardiovascular disease

Individuals with a history of cardiovascular disease were assigned to a health status of either stable angina, unstable angina, myocardial infarction, or stroke based on responses to health survey for England responses to long standing conditions. Individuals reporting stroke were assigned to stroke, heart attak/angina to unstable angina and MI at random using distributions estimated in the statins HTA (12).

#### Baseline EQ-5D

Data were imputed using multiple imputation methods from the mice package in R. Predictive mean methods imputation methods were used to impute missing data based on age, socioeconomic status, anti-hypertensive treatment, diabetes diagnosis, anthropometric, metabolic data and EQ-5D within the dataset. Limiting the number of relationships to include in the imputation method was necessary to reduce the computational time required by the imputation.

# GP Attendance in the General Population

GP visit frequency was simulated in the dataset to estimate healthcare utilisation for the general population and the costs associated with these GP appointments, in order to complete a comprehensive cost-effective analysis in the model. A statistical model of GP attendance, conditional on characteristics that are associated, such as age, ethnicity and comorbidities, was developed. If those with life limiting health concerns visit the GP more, then a change in the presence of these conditions will change the number attending GP appointments and thus creating an overall change in primary-care costs.

A negative binomial model was used to generate count data and a skewed distribution was observed in the dataset.

|  | $\mu_{i}=exp(x_{i}\beta)$ |  |
| --- | --- | --- |

The dispersion parameter of the Negative Binomial distribution $v_{i}$ was sampled from a gamma distribution with mean 1 and variance $\alpha$ based on estimates reported in Table S7. The dose was estimated from the Poisson function.

|  | $p\left( Y=y \vert y>0,x \right)=\frac{\left( v_{i}\mu_{i} \right)^{y}e^{-\left( v_{i}\mu_{i} \right)}}{y!}$ |  |
| --- | --- | --- |

We used data from the Health Survey for England 2019, which collected a patient reported variable on the number times they attended a GP in the last year. It also collected demographic and health related information, including whether they had diabetes or not. This would allow the GP attendance model to represent the relevant population within the Diabetes Treatment Model. While explicit variables indicating comorbidities were not included, variables indicating which related medications they are taken is reported. Whether the patient has a prescription for antidepressants, lipid lowering medication or anti-hypertensive medication is thought to have a relation with GP attendance, especially considering the policies relating to regular medication reviews. A variable indicating whether an individual has a prescription for these medications is included in the model. Finally, to account for any comorbidities, a variable indicating whether they have a life limiting illness was included. The population included were adults above 20 years old. The characteristics of the study population are reported in Table S6.

Table S6: Characteristics of HSE 2019

| **Variable** | **Observations** | **Mean** | **SD** |
| --- | --- | --- | --- |
| GP Visits | 7,746 | 2.96 | 2.99 |
| Age (20+) | 7,855 | 52.52 | 17.69 |
| Male | 7,855 | 0.46 | 0.50 |
| Black | 7,826 | 0.03 | 0.18 |
| Asian | 7,826 | 0.10 | 0.30 |
| Ethnic Minority | 7,826 | 0.17 | 0.37 |
| BMI | 6,470 | 26.24 | 6.60 |
| Diabetes | 7,848 | 0.08 | 0.27 |
| Life Limiting Illness | 7,847 | 0.24 | 0.43 |
| Lipid Lowering Medication | 4,774 | 0.15 | 0.36 |
| Anti-Hypertensive Medication | 4,768 | 0.24 | 0.43 |
| Antidepressant | 4,774 | 0.10 | 0.30 |

The coefficients of the Negative Binomial model, described in Table 6, were used to calculate the first parameter of the Negative Binomial distribution$\mu_{i}$ based on an individual’s characteristics. The dispersion parameter sampled from the gamma distribution using the alpha reported in Table S7. Three regression specifications are reported in Table 6. Model 1 regresses GP visits against age, sex, ethnicity, BMI, whether they have diabetes and whether they have a life limiting illness. Model 2 regresses GP visits against all parameters in model 1, but with the additional variable indicating if they are prescribed antidepressant medications. Finally Model 3 included whether they are prescribed CVD medication (lipid lowering and anti-hypertensive medication) on top of Model 2.

Table S7: GP attendance reported in the HSE 2019

|  | **Model 1** | | **Model 2** | | **Model 3** | |
| --- | --- | --- | --- | --- | --- | --- |
|  | Mean | SE | Mean | SE | Mean | SE |
| Age | 0.00263*** | (0.000725) | 0.00195** | (0.000880) | -0.00221** | (0.000989) |
| Male | -0.211*** | (0.0246) | -0.177*** | (0.0295) | -0.220*** | (0.0297) |
| Black | 0.135* | (0.0726) | 0.296*** | (0.0906) | 0.303*** | (0.0902) |
| Asian | 0.211*** | (0.0438) | 0.247*** | (0.0562) | 0.242*** | (0.0557) |
| BMI | 0.0105*** | (0.00207) | 0.00866*** | (0.00246) | 0.00548** | (0.00247) |
| Diabetes | 0.282*** | (0.0438) | 0.238*** | (0.0517) | 0.112** | (0.0533) |
| Life Limiting Illness | 0.700*** | (0.0263) | 0.587*** | (0.0320) | 0.553*** | (0.0319) |
| Antidepressants |  |  | 0.406*** | (0.0417) | 0.407*** | (0.0413) |
| Lipid lowering Medication |  |  |  |  | 0.207*** | (0.0424) |
| Anti-Hypertensive Medication |  |  |  |  | 0.224*** | (0.0392) |
| Constant | 0.422*** | (0.0704) | 0.480*** | (0.0844) | 0.727*** | (0.0883) |
| Alpha | 0.547 | (0.0180) | 0.494 | (0.0206) | 0.476 | (0.0202) |
| LnAlpha | -0.603*** | (0.0330) | -0.705*** | (0.0417) | -0.741*** | (0.0424) |
| N | 6,378 | | 4,215 | | 4,209 | |
| Pseudo R^2^ | 0.0357 | | 0.0392 | | 0.0434 | |
| Standard errors in parentheses (*** p<0.01, ** p<0.05, * p<0.1) | | | | | | |

The average number of GP visits was approximately 3 times in the year. The number of GP visits was statistically significantly related with all variables included. Individuals that are Black or Asian are more likely to attend the GP, while males are less likely. Those with a higher BMI, a life limiting illnesses or are taking CVD medication and antidepressants go to the GP more often. The large positive relationships between GP attendance and indicators of ill health justify their inclusion. An unexpected negative relationship is seen in age when including CVD medication variables. This could be explained by the strong positive correlation between age and taking CVD related medication.

Model 3 was chosen to model GP utilisation due to the high statistical significance of all variables and the greater Pseudo R^2^ value. The variable covariance matrix from Model 3 is reported in Table S8.

Table S8: Variance-covariance matrix for GP Attendance, Model 3

|  | Age | Male | Black | Asian | BMI | Diabetes | Life Limiting Illness | Antidepressants | Lipid Lowering Med | Anti-Hypertensive Med | Constant | LnAlpha |
| --- | --- | --- | --- | --- | --- | --- | --- | --- | --- | --- | --- | --- |
| Age | 0.0000 |  |  |  |  |  |  |  |  |  |  |  |
| Male | -0.0000 | 0.0009 |  |  |  |  |  |  |  |  |  |  |
| Black | 0.0000 | 0.0001 | 0.0081 |  |  |  |  |  |  |  |  |  |
| Asian | 0.0000 | 0.0000 | 0.0003 | 0.0031 |  |  |  |  |  |  |  |  |
| BMI | 0.0000 | -0.0000 | -0.0000 | 0.0000 | 0.0000 |  |  |  |  |  |  |  |
| Diabetes | -0.0000 | -0.0000 | -0.0002 | -0.0001 | -0.0000 | 0.0028 |  |  |  |  |  |  |
| Life Limiting Illness | -0.0000 | 0.0000 | -0.0000 | 0.0000 | -0.0000 | -0.0001 | 0.0010 |  |  |  |  |  |
| Antidepressants | 0.0000 | 0.0001 | 0.0002 | 0.0002 | -0.0000 | -0.0001 | -0.0003 | 0.0017 |  |  |  |  |
| Lipid lowering Medication | -0.0000 | -0.0002 | 0.0001 | -0.0000 | -0.0000 | -0.0005 | -0.0000 | -0.0001 | 0.0018 |  |  |  |
| Anti-Hypertensive Medication | -0.0000 | -0.0000 | -0.0000 | -0.0000 | -0.0000 | -0.0002 | -0.0001 | 0.0000 | -0.0005 | 0.0015 |  |  |
| Constant | -0.0001 | -0.0003 | -0.0005 | -0.0009 | -0.0002 | 0.0004 | 0.0000 | -0.0001 | 0.0006 | 0.0008 | 0.0078 |  |
| **LnAlpha** | 0.0000 | -0.0000 | -0.0000 | 0.0000 | 0.0000 | 0.0000 | 0.0000 | 0.0000 | 0.0000 | 0.0000 | -0.0000 | 0.0018 |

# Longitudinal Trajectories of Metabolic Risk Factors

Three sets of statistical analyses were used to describe metabolic trajectories for individuals in the model. Analysis of the WRAP-UP trial was used to simulate metabolic trajectories in the first 5 years after attending a weight management programme. The trajectories in the subsequent years were simulated statistical models from the Whitehall II cohort for adults aged less than 60 and English Longitudinal Study of Ageing for those aged over 60. A summary for each set of metabolic trajectory models are provided below.

## WRAP trial Data

The effectiveness of the weight watchers intervention was estimated from differences in BMI, systolic blood pressure, total cholesterol and HbA1c using the WRAP trial data. Separate ordinary least squares regression models were estimated for the changes in metabolic risk factors over time between trial timepoints. For BMI we estimate change in BMI from baseline to 12 months, 12 months to 24 months, and 24 months and 5 years. For the HbA1c trajectory trial data estimates the changes between baseline and 12 months, and 12 months and 5 years. The regressions included a covariate for the 12 and 52 treatment groups. The regressions also adjusted for age, sex and type 2 diabetes. The WRAP trail data wer used to simulate systolic blood pressure and cholesertoal for the first 12 months only. An interaction term for treatment effect in diabetics was included to allow differential treatment effect in diabetics and non-diabetics. The regression models for the changes from 12 months to 5 years include covairates for the treatment groups, a covariate for then change in the previous period, age, sex, and type-2 diabetes. We assumed data was Missing at Random (MAR). The regression analyses were estimated in STATA version 15. The covariance matrices were used in the Probabilistic Sensitivity Analysis to describe uncertainty. The coefficients from the BMI regression analysis of the trial data are reported in Table S9. The change in BMI at 12 months was statistically significantly lower for the 12 and 52 Weight Watchers intervention. In the simulation we assume all individuals have recieved a weight loss intervention, and we characterise the ongoing effects on their metabolic risks using the 12 week weight watchers coefficient effects on BMI, HbA1c, systolic blood pressure and cholesterol.

Table S9: The regression coefficients for the BMI trajectories (N=1267)

|  | Change at 12 months | | Change at 24 months | | Change at 60 months | |
| --- | --- | --- | --- | --- | --- | --- |
|  | Coefficient | p-value | Coefficient | p-value | Coefficient | p-value |
| Age | -0.029 | 0.000 | -0.0001 | 0.979 | -0.018 | 0.064 |
| Sex | -0.410 | 0.049 | -0.173 | 0.218 | -0.181 | 0.478 |
| BMI at baseline | -0.088 | 0.000 | -0.011 | 0.404 | -0.062 | 0.015 |
| Diabetes | 0.491 | 0.069 | -0.412 | 0.031 | -0.182 | 0.600 |
| 12 week Weight Watchers | -0.551 | 0.054 | 0.167 | 0.386 | -0.434 | 0.222 |
| 52 week Weight Watchers | -1.393 | 0.000 | 0.273 | 0.164 | -0.269 | 0.461 |
| Change at 12 months |  |  | -0.139 | 0.000 | -0.408 | 0.000 |
| Change at 24 months |  |  |  |  | -0.376 | 0.000 |
| Constant | 3.572 | 0.000 | 0.770 | 0.177 | 3.722 | 0.001 |

The coefficients from the HbA1c regression analysis of the trial data are reported in Table S10. The change in HbA1c at 12 months was not statistically significantly lower for the 12 and 52 Weight Watchers intervention in non-diabetic. However, the diabetes and treatment effect interaction terms suggest that the interventions have a significant effect on HBA1c in diabetics. In the simulation we assume that all participants benefit from the coefficient for 12 weeks of weight watchers.

Table S10: The regression coefficients for the hbA1c (%) trajectories (N=1267)

|  | Change at 12 months | | Change at 5 years | |
| --- | --- | --- | --- | --- |
|  | Coefficient | p-value | Coefficient | p-value |
| Age | 0.005 | 0.004 | -0.004 | 0.250 |
| Sex | -0.011 | 0.811 | -0.217 | 0.008 |
| HbA1c at baseline | -0.385 | 0.000 | -0.134 | 0.059 |
| Diabetes | 0.713 | 0.000 | 0.156 | 0.630 |
| 12 week Weight Watchers | -0.048 | 0.507 | -0.062 | 0.643 |
| 52 week Weight Watchers | -0.089 | 0.220 | 0.022 | 0.869 |
| 12 week Weight Watchers*Diabetes | -0.336 | 0.037 | 0.803 | 0.015 |
| 12 week Weight Watchers*Diabetes | -0.688 | 0.000 | 0.478 | 0.160 |
| Change at 12 months |  |  | -0.068 | 0.433 |
| Constant | 1.858 | 0.000 | 1.207 | 0.006 |

We use the WRAP trajectories for systolic blood pressure and cholesterol in the 12 months following a weight loss programme only. Beyond 12 months we use the Whitehall II and ELSA trajectories because evidence from the WRAP suggests that there was no sustain differences in treatment and controls beyond 12 months.

Table S11: The regression coefficients for the systolic blood pressure and cholesterol trajectories (N=1267)

|  | Change at 12 months in systolic blood pressure | | Change at 12 months in total cholesterol | |
| --- | --- | --- | --- | --- |
|  | Coefficient | Coefficient | Coefficient | p-value |
| Age | 0.143 | 0.004 | 0.004 | 0.060 |
| Sex | -3.153 | 0.194 | 0.194 | 0.002 |
| Systolic blood pressure at baseline | -0.468 | -0.242 |  |  |
| Cholesterol at baseline |  |  | -0.242 | 0.000 |
| Diabetes | 0.505 | 0.687 | -0.044 | 0.564 |
| 12 week Weight Watchers | 0.891 | 0.508 | -0.029 | 0.741 |
| 52 week Weight Watchers | 0.323 | 0.809 | -0.084 | 0.344 |
| IMD 2 | -1.381 | 0.451 |  |  |
| IMD 3 | -1.594 | 0.337 |  |  |
| IMD 4 | -4.563 | 0.004 |  |  |
| IMD 5 | -4.521 | 0.004 |  |  |
| Change at 12 months |  |  |  |  |
| Constant | 55.498 | 0.000 | 0.654 | 0.003 |

## Whitehall II Data Analysis

Changes in BMI, latent blood glucose, total cholesterol, HDL cholesterol and SBP were estimated from statistical analysis of the Whitehall II cohort. The growth factors for all 5 risk factors were estimated using parallel latent growth modelling. This enabled the growth factors for BMI to be implemented as covariates for the growth processes of glycaemia, systolic blood pressure, and total cholesterol^[[1]](#footnote-1)^. The structural assumptions of the analysis are described in more detail below.

In the Whitehall II data analysis it was assumed that individuals have an underlying level of glycaemia, which cannot be observed but can be measured by HbA1c, FPG, and 2-hour glucose. This underlying propensity for diabetes is referred to as latent glycaemia. The statistical model estimated the unobservable latent glycaemia, and from this identified associations with test results for HbA1c, FPG, and 2-hour glucose. The longitudinal changes in BMI, glycaemia, SBP, total cholesterol and HDL cholesterol could then be estimated through statistical analysis.

These growth factors are conditional on several individual characteristics including age, sex, ethnicity, smoking, family history of CVD, and family history of type 2 diabetes. We related the effect of changes in BMI to changes in glycaemia, SBP and total cholesterol. However, if an intervention is known to be effective in reducing BMI and the other metabolic risk factors, the Whitehall II model is adjusted to temporarily remove the indirect effect of the intervention through BMI. This ensures that the effectiveness of the intervention is not over-estimated. Unobservable heterogeneity between individual growth factors not explained by patient characteristics was incorporated into the growth models as random error terms. Correlation between the random error terms for glycaemia, total cholesterol, HDL cholesterol and systolic blood pressure was estimated from the Whitehall II cohort. This means that in the simulation, an individual with a higher growth rate for glycaemia was more likely to have a higher growth rate of total cholesterol and SBP.

An advantage of this parallel growth analysis is that it was able to estimate the effect of growth in BMI on the other metabolic risk factors. The statistical analysis also described the correlation between changes in glycaemia, SBP, total cholesterol and HDL cholesterol. As a consequence, the growth factor random error terms were not assumed to be independent and were sampled from a multivariate normal distribution$\boldsymbol{\upsilon}\sim N(0,\Omega)$. Estimates for the covariance matrix are derived from the covariance estimates reported in the statistical analysis.

The baseline observations for BMI, HbA1c, SBP, cholesterol and HDL cholesterol were extracted from the Health Survey for England 2014 in order to simulate a representative sample of the UK population. The predicted intercept for these metabolic risk factors was estimated using the Whitehall II analysis to give population estimates of the individuals’ starting values, conditional on their characteristics. The difference between the simulated and observed baseline risk factors was taken to estimate the individuals’ random deviation from the population expectation. The individual random error in the slope trajectory was sampled from a conditional multivariate normal distribution to allow correlation between the intercept and slope random errors.

Figure S2: Path analysis of final statistical analysis of the Whitehall II cohort

## ELSA Data Analysis

Changes in BMI, HbA1c, systolic blood pressure, total cholesterol and HDL cholesterol were estimated from a statistical analysis of the ELSA cohort. The changes with age were estimated using independently estimate random coefficient growth models in Stata 13. The growth trajectory models for the metabolic risk factor were estimated under the statistical framework of growth curve modelling (GCM) (13). GCM is an approach to using longitudinal data to estimate shape and rate of change over time. GCM was chosen because it can allow modelling of variability in participants fixed and slope parameters. The growth factors for the metabolic risk factors were assumed to vary between individuals to allow unobservable random effects to describe the heterogeneity in intercept and slope parameters. Assessment of the data indicated that there was significant variance in the intercept (risk factor starting value) and slope (change in risk factor over time) for all metabolic risks. The growth factor models without covariates were specified as.

$$Y_{ij}=\left( \beta_{1}+\zeta_{1j} \right)+\left( \beta_{2}+\zeta_{2j} \right)t_{ij}+\epsilon_{ij}$$

Where Y describes the observed metabolic risk factor for individual i at time j, $\beta_{1}$ is the population mean intercept, and $\beta_{2}$ population mean slope. The random factors $\zeta_{1j}$ and $\zeta_{2j}$ describe the random variability across individuals in the intercept and slopes respectively. The statistical models were weighted for selection bias using nurse visit weights for BMI and systolic blood pressure and blood sample weights for Total Cholesterol, HDL Cholesterol and HbA1c supplied by the ELSA dataset to improve the representativeness of the analysis for an English population.

The model assumed that BMI growth was quadratic with age, due to trends observed in the data and in other cohorts (14). HbA1c, systolic blood pressure, total cholesterol and HDL cholesterol were assumed to be linear with time. All model intercepts and slopes were adjusted for sex, smoking status, deprivation and ethnicity. Anti-hypertensive treatment was included as an additional covariate in the systolic blood pressure model. Unfortunately data on statin were not available for any wave except wave 6, therefore we did not include this as a covariate in the analysis. Covariates were included in the final model if the variable was statistical significant with a p-value less than 0.1.

## BMI Trajectory

At baseline, BMI estimates from the HSE determine an individual’s BMI. In the first 5 years of the simulation the individual’s BMI will follow the trajectory determined by the analysis of the WRAP trial data . Beyond 5 years if the individual is aged 60 years or less annual changes in BMI are calculated from the Whitehall II study based on population average changes for the individual and a sampled random coefficient factor. From ages 61 and over the ELSA BMI statistical model is used estimate their older age trajectory in BMI. New random coefficient growth factors are estimated based on the covariance structure of the ELSA random intercept and slope. As a consequence, current BMI status is informative in determining the future trajectory of BMI.

## Glycaemic Trajectory in Non-Diabetics

At baseline, HbA1c estimates for HbA1c are used to determine an individual’s HbA1c and glycaemic status. For individuals aged 60 years or less the Whitehall II study is used to estimate annual changes in HbA1c, and through latent glycaemia FPG, and 2-hr glucose observations. In the Whitehall II analysis we assume that changes in latent glycaemia have a quadratic relationship with time. The Whitehall II models allow random coefficient factors for growth in glycaemia for an individual and measurement error in test results according to estimated parameters from the Whitehall II analysis. For individuals aged 61 and over the ELSA statistical model is used to estimate an individuals linear changes in HbA1c every year. Random coefficient growth factors are re-estimated using the bivariate covariance structure from the ELSA HbA1c growth model. It is not possible to estimate FPG and 2-hr glucose using the ELSA statistical models.

**HbA1c trajectory in undiagnosed diabetes**

At diabetes onset HbA1c is assumed to increase at a rate of 0.318% per year based on analyses of undiagnosed diabetes patients in the UK Biobank (15). In this study they identified a group of undiagnosed diabetes cases. These cases of undiagnosed diabetes were followed up from GP records to identify the time and median HBA1c score at diagnosis. On study entry the median HbA1c ws 6.8% (IQR 6.6-7.4). Median time to diabete diagnosis was 2.2 years (IQR 2.0 – 2.4), and mean HbA1c was 7.5% (IQR 6.8-9.5).

## HbA1c trajectory in type 2 diagnosed diabetics

Following a diagnosis of diabetes in the simulation all individuals experience an initial fall in HbA1c due to changes in diet and lifestyle as observed in the UKPDS trial (16). We have estimated the expected change in HbA1c conditional on HbA1c at diagnosis by fitting a simple linear regression to three aggregate outcomes reported in the study. These showed that the change in HbA1c increases for higher HbA1c scores at diagnosis. The regression parameters to estimate change in HbA1c are reported in Table S12.

Table S12: Estimated change in HbA1c following diabetes diagnosis

|  | Mean | Standard error |
| --- | --- | --- |
| Change in HbA1c Intercept | -2.99 | .048 |
| HbA1c at baseline | 0.55 | 0.40 |

After this initial reduction in HbA1c the longitudinal trajectory of HbA1c is estimated using the UKPDS outcomes model (16) rather than the Whitehall II statistical analysis. The UKPDS dataset is made up of a newly diagnosed diabetic population. As part of the UKPDS Outcomes model, longitudinal trial data were analysed using a random effects model. The coefficients of the model are reported in Table S13.

Table S13: Coefficient estimates for HbA1c estimated from UKPDS data

|  | Mean Coefficient | Coefficient standard error |
| --- | --- | --- |
| Intercept | -0.024 | 0.017 |
| Log transformation of year since diagnosis | 0.144 | 0.009 |
| Binary variable for year after diagnosis | -0.333 | 0.05 |
| HbA1c score in last period | 0.759 | 0.004 |
| HbA1c score at diagnosis | 0.085 | 0.004 |

The model can be used to predict HbA1c over time from the point of diagnosis. The model suggests that HbA1c increases with time. A graph illustrating change in HbA1c over time from two different HbA1c levels at diagnosis is illustrated in Figure S3.

Figure S3: Trajectory of HbA1c estimated from UKPDS longitudinal model

##

## Total Cholesterol and HDL Cholesterol Trajectories in Individuals not receiving Statins

At baseline, an individual’s total and HDL cholesterol is determined from the HSE 2014 data. In the simulation, individuals aged 60 years and younger have annual changes in total and HDL cholesterol according to the estimates from the statistical analysis of the Whitehall II cohort. The slope of total and HDL cholesterol are assumed to be linear with time. These growth factors are estimated in the model to be conditional on cholesterol at baseline, age at baseline, sex, and an error parameter to reflect unobservable variability in growth trajectories between individuals. As with latent glycaemia, changes in total cholesterol are also influenced by the trajectory of BMI. For individuals aged 61 and over the ELSA statistical models for Total and HDL cholesterol are used to estimate annual change in cholesterol. As individuals transition between the trajectory models the random coefficient factors are updated allowing current observations to inform the trajectories in Total and HDL cholesterol.

## Total Cholesterol and HDL Cholesterol Trajectories in Individuals receiving Statins

During the simulation process, individuals are prescribed statins to reduce their risk of cardiovascular disease. It is assumed within the model that the statins are effective in reducing an individual’s total cholesterol, and an average effect is applied to all patients receiving statins. A recent HTA reviewed the literature on the effectiveness and cost-effectiveness of statins in individuals with acute coronary syndrome (17). This report estimated the change in LDL cholesterol for four statin treatments and doses compared with placebo from a Bayesian meta-analysis. The analysis estimated a reduction in LDL cholesterol of -1.45 for simvastatin. This estimate was used to describe the effect of statins in reducing total cholesterol. It was assumed that the effect was instantaneous upon receiving statins and maintained as long as the individual receives statins. It was also assumed that individuals receiving statins no longer experienced annual changes in total cholesterol. HDL cholesterol was assumed constant over time if patients receive statins.

Non-adherence to statin treatment is a common problem. Two recent HTAs reviewed the literature on continuation and compliance with statin treatment. They both concluded that there was a lack of adequate reporting, but that the proportion of patients fully compliant with treatment appears to decrease with time, particularly in the first 12 months after initiating treatment, and can fall below 60% after five years (12, 17). Although a certain amount of non-compliance is included within trial data, clinical trials are not considered to be representative of continuation and compliance in general practice. A yearly reduction in statin compliance used in the HTA analysis is reported in Table S14. It is based on the published estimate of compliance for the first five years of statin treatment for primary prevention in general clinical practice (17). Compliance declines to a minimum of 65% after five years of treatment. It is assumed that there is no further drop after five years.

Table S14: Proportion of patients assumed to be compliant with statin treatment, derived from Table 62 in (17)

| Year after statin initiation | 1 | 2 | 3 | 4 | 5 |
| --- | --- | --- | --- | --- | --- |
| Proportion compliant | 0.8 | 0.7 | 0.68 | 0.65 | 0.65 |

In the simulation, we assume in the base case that only 65% of individuals initiate statins when they are deemed eligible. However those that initiate statins remain on statins for their lifetime. Those who refuse statins may be prescribed them again at a later date.

## SBP Trajectories in Individuals Not receiving Anti-hypertensive treatment

At baseline an individual’s SBP is determined from the HSE 2014 data. In the simulation, individuals’ aged 60 and younger experience SBP changes every year according to the estimates from the statistical analysis of the Whitehall II cohort. The annual change in SBP is assumed to be linear with time. The growth factors are estimated in the model to be conditional on SBP at baseline, age at baseline, sex, ethnicity, family history of cardiovascular disease, smoking and an error parameter to reflect unobservable variability in growth trajectories between individuals. From ages 61 onwards the ELSA statistical model for systolic blood pressure is used to estimate annual changes. The random coefficient factors are updated using the bivariate covariance matrix for intercept and slope factors.

## SBP Trajectories in Individuals receiving anti-hypertensive treatment

During the simulation process, if individuals are identified as having SBP higher than 160mm Hg, or SBP higher than 140mm Hg with comorbid diabetes, cardiovascular disease, or 10 year risk of cardiovascular disease greater than 20%, they will be prescribed anti-hypertensive treatment in line with the National Institute for Health and Care Excellence (NICE) guidelines (18). The change in SBP following initiation of calcium channel blockers was estimated in a meta-analysis of anti-hypertensive treatments (19). This study identified an average change in SBP of -8.4 for monotherapy with calcium channel blockers. In the simulation model it is assumed that this reduction in SBP is maintained for as long as the individual receives anti-hypertensive treatment. Once an individual is receiving anti-hypertensive treatment it is assumed that their SBP is stable and does not change over time, which implicitly assumes that patients continue to be well managed for their hypertension. For simplicity we do not explicitly simulate treatment switching. The assumed zero flat trajectory in systolic blood pressure whilst receiving anti-hypertensives is supported by the analysis of the ELSA dataset in which self-reported use of anti-hypertensives was included as a covariate for age-related change in systolic blood pressure. The analysis found that most of the observed changes in systolic blood pressure were removed if individuals were taking anti-hypertensives.

## Metabolic Risk factor screening

We assume that individuals eligible for anti-hypertensive treatment or statins will be identified through opportunistic screening if they meet certain criteria and attend the GP for at least one visit in the simulation period.

1. Individuals with a history of cardiovascular disease;
2. Individuals with a major microvascular event (foot ulcer, blindness, renal failure or amputation);
3. Individuals with diagnosed diabetes;
4. Individuals identified with Impaired Glucose Regulation;
5. Individuals with systolic blood pressure greater than 160mmHg.

The base case has been designed to represent a health system with moderate levels of screening for hypertension, and dyslipidaemia. Alternative assumptions for more or less intensive opportunistic screening can be assumed.

## Diagnosis and Treatment Initiation

It is assumed that there are three, non-mutually exclusive outcomes from the vascular checks or opportunistic screening. Firstly, that the patient receives statins to reduce cardiovascular risk. Secondly, that the patient has high blood pressure and should be treated with anti-hypertensive medication. The following threshold estimates were used to determine these outcomes.

1. Statins are initiated if the individual has greater than or equal to 20% 10 year CVD risk estimated from the QRISK2 2012 algorithm (20).
2. Anti-hypertensive treatment is initiated if systolic blood pressure is greater than 160. If the individual has a history of CVD, diabetes or a CVD risk >20%, the threshold for systolic blood pressure is 140 (18).

## Diabetes Diagnosis

The HSE repeatedly demonstrates that a large proportion of the English population have a HbA1c test score above 6.5%, and do not report a diagnosis of diabetes. Therefore, it is important to allow some undiagnosed cases of diabetes to persist in the model. Diabetes onset is simulating using the QDiabetes 2018 risk algorithm (21). The model estimates the QDiabetes score, with HBA1c covariates, for undiagnosed individuals within the population in each annual cycle. This score describe the individuals 10 year probability of being diagnosed with diabetes conditional on demographic, socioeconomic, ethnicity, medical history and HbA1c score. The QDiabetes 2018 model was chosen to describe individuals risk of diabetes in the model to describe differential risks across individuals. The one-year probability of diabetes uses the coefficients from the QDiabetes 2018 tool, applied to a baseline risk calibrated to observed data on the rate of diabetes diagnoses in this population. In each cyle an individual is diagnosed with Diabetes if they meet the following criteria.

- Diagnosed hypertension
- Micro/macro vascular diabetes complication
- HbA1c above simulated HbA1c at diagnosis, median 7.5% (15)

The QDiabetes 2018 risk equation can be used to calculate the probability of a cardiovascular event including: coronary heart disease (angina or myocardial infarction), stroke, or transient ischaemic attacks, fatality due to cardiovascular disease. The equation estimates the probability of a cardiovascular event in the next period conditional on the coefficients listed in Table 11. The equation for the probability of an event in the next period is calculated as

$$p\left( Y=1 \right)=1-{S(1)}^{\theta}$$

$$\theta=\sum\beta X$$

The probability of an event is calculated from the survival function at 1 year raised to the power of $\theta$, where $\theta$ is the sum product of the coefficients reported in Table S16 multiplied by the individual’s characteristics. Underlying survival curves for men and women were extracted from the QDiabetes 2018 open source file. Mean estimates for the continuous variables were also reported in the open source files.

Table S15: Coefficients from the 2018 QDiabetes risk equation and estimated standard errors

| Estimated coefficients adjusting for individual characteristics | | | | | | | | | |
| --- | --- | --- | --- | --- | --- | --- | --- | --- | --- |
|  | Women | | Men | |  | Women | | Men | |
| Covariates | Mean | Standard error | Mean | Standard error | Interaction terms | Mean | Standard error | Mean | Standard error |
| White | 0.0000 |  | 0.0000 |  | Age1*former smoker | -0.8125 | 0.035 | -1.0013 | 0.776 |
| Indian | 0.5991 |  | 0.6757 |  | Age1*light smoker | -0.9085 | 0.066 | -0.8916 | 3.341 |
| Pakistani | 0.7832 |  | 0.8315 |  | Age1*moderate smoker | -1.8558 | 0.231 | -1.7075 | 3.075 |
| Bangladeshi | 1.1947 |  | 1.0969 |  | Age1*Heavy smoker | 0.6023 | 0.308 | 0.4507 | 3.529 |
| Other Asian | 0.7142 |  | 0.7682 |  | Age1*AF | -0.0345 | 0.922 | -0.1085 | 1.406 |
| Caribbean | 0.1195 |  | 0.2090 |  | Age1*renal disease | -0.2728 | 0.528 | -0.6141 | 3.403 |
| Black African | 0.0137 |  | 0.3809 |  | Age1*hypertension | 25.4412 | 0.450 | 27.6706 | 6.793 |
| Chinese | 0.5709 |  | 0.3424 |  | Age1*Diabetes | -6.8076 | 0.369 | -7.4006 | 2.558 |
| Other | 0.1709 |  | 0.2205 |  | Age1*BMI | 0.0005 | 0.617 | 0.0002 | 0.654 |
| Non-smoker | 0.0000 |  | 0.0000 |  | Age1*family history CVD | 0.0009 | 0.050 | 0.0007 | 3.584 |
| Former smoker | 0.0658 |  | 0.1159 |  | Age1*SBP | 0.0023 | 0.003 | 0.0014 | 0.030 |
| Light smoker | 0.1458 |  | 0.1462 |  | Age1*Townsend | -0.0043 | 0.007 | -0.0012 | 0.510 |
| Moderate smoker | 0.1526 |  | 0.1078 |  | Age2*former smoker | 0.0001 |  | 0.0002 |  |
| Heavy smoker | 0.3079 |  | 0.1985 |  | Age2*light smoker | 0.0004 |  | 0.0005 |  |
| Age 1* | 3.5655 |  | 4.0193 |  | Age2*moderate smoker | -0.0523 |  | -0.0592 |  |
| Age 2* | -0.0056 |  | -0.0048 |  | Age2*Heavy smoker | 0.0141 |  | 0.0156 |  |
| BMI* | 2.5043 |  | 0.8183 |  | Age2*AF | -0.8125 |  | -1.0013 |  |
| Ratio Total / HDL chol | -0.0429 |  | -0.1256 |  | Age2*renal disease | -0.9085 |  | -0.8916 |  |
| SBP | 8.7368 |  | 8.0512 |  | Age2*hypertension | -1.8558 |  | -1.7075 |  |
| Townsend | -0.0782 |  | -0.1465 |  | Age2*Diabetes | 0.6023 |  | 0.4507 |  |
| AF | 0.0359 |  | 0.0252 |  | Age2*BMI | -0.0345 |  | -0.1085 |  |
| Rheumatoid arthritis | 0.5498 |  | 0.4554 |  | Age2*family history CVD | -0.2728 |  | -0.6141 |  |
| Renal disease | 0.1687 |  | 0.1382 |  | Age2*SBP | 25.4412 |  | 27.6706 |  |
| Hypertension | 0.1644 |  | 0.1455 |  | Age2*Townsend | -6.8076 |  | -7.4006 |  |
| Diabetes | 1.1250 |  | 0.2596 |  |  |  |  |  |  |
| Family history of CVD | 0.2891 |  | 0.2852 |  |  |  |  |  |  |
| AF Atrial Fibrillation CVD Cardiovascular disease SBP systolic blood pressure * covariates transformed with fractional polynomials | | | | | | | | | |

#

# Comorbid Outcomes and Mortality

In every model cycle individuals within the model are evaluated to determine whether they have a clinical event, including mortality, within the cycle period. In each case the simulation estimates the probability that an individual has the event and uses a random number draw to determine whether the event occurred.

## Cardiovascular Disease

### First Cardiovascular event

Several statistical models for cardiovascular events were identified in a review of economic evaluations for diabetes prevention (22). The UKPDS outcomes model (23), Framingham risk equation (24) and QRISK2 (25) have all been used in previous models to estimate cardiovascular events. The Framingham risk equation was not adopted because, unlike the QRISK2 model, it is not estimated from a UK population. The UKPDS outcomes model would be ideally suited to estimate the risk of cardiovascular disease in a population diagnosed with type 2 diabetes. Whilst this is an important outcome of the cost-effectiveness model, there was concern that it would not be representative of individuals with normal glucose tolerance or impaired glucose regulation. Recent analyses show that the UKPDS over-predicts cardiovascular outcomes in newly diagnosed diabetes patients (26). It was important that reductions in cardiovascular disease risk in these populations were represented to capture the population-wide benefits of public health interventions. The QRISK2 model was selected for use in the cost-effectiveness model because it is a validated model of cardiovascular risk in a up to date UK population and could be used to generate probabilities for diabetic and non-diabetic populations. We considered using the UKPDS outcomes model specifically to estimate cardiovascular risk in patients with type 2 diabetes. However, it would not be possible to control for shifts in absolute risk generated by the different risk scores due to different baselines and covariates. This would lead to some individuals experiencing counterintuitive and favourable shifts in risk after onset of type 2 diabetes. Therefore, we decided to use diabetes as a covariate adjustment to the QRISK2 model to ensure that the change in individual status was consistent across individuals.

The probability of the first cardiovascular event is estimated from the QRISK2 predicted model of cardiovascular disease (20). The QRISK2 is a validated risk prediction algorithm to identify individuals at high risk of cardiovascular disease. The algorithm was developed from UK data and incorporates social deprivation and ethnicity. We accessed the coefficients and algorithm from the online QRISK website (27). The QRISK2 equation estimates the probability of a cardiovascular event in the next 10 years conditional on ethnicity, smoking status, age, BMI, ratio of total/HDL cholesterol, Townsend score, atrial fibrillation, rheumatoid arthritis, renal disease, hypertension, diabetes, and family history of cardiovascular disease. Data on all these variables was available from the HSE 2014.

### First Cardiovascular event calibration

In version 4 of the model the QRISK2 algorithm was updated with 2014 risk coefficients. The 2014 version did not report the 1 year survival function. For the 2014 version we estimated the 1 year parameter using a simple calibration process to match the CVD incidence from the WRAP trial after 5 years of follow-up to the simulated incidence of CVD events. A simple calibration approach was taken to identify a parameter estimate for the 1 year survival parameter for men and women that would simulate the estimated CVD incidence reported from the 5 year follow-up data within a 2% margin or error. This data was based on cardiovascular events to include, MI, percutaneous coronary intervention (PCI), Experienced bypass (CABG), invasive cardiovascular procedure, Transient Ischaemic Attack or stroke reported in GP records.

At 5 years follow-up GP records for CVD history were available for for 859 women, and 408 men. Of those 14 and 18 had experienced a CVD event respectively. For the calibration were identified survival parameters for men and women that would generate an incidence of 3.26 per 1000 person years for women and 8.824 per 1000 person years for men in the brief intervention simulation. These parameters were estimated with a simple iterative process simulating 20,000 randomly selected indiviudals. The final estimates were tested against other random samples. Starting values were selected from using the survival parameters reported in the QRISK 2012 source code.

### First Cardiovascular event inputs

Table S16 reports the coefficient estimates for the QRISK2 algorithm. The standard errors were not reported within the open source code. Where possible, standard errors were imputed from a previous publication of the risk equation (28). Coefficients that were not reported in this publication were assumed to have standard errors of 20%.

Table S16: Coefficients from the 2014 QRISK2 risk equation and estimate standard errors

|  | | Estimated coefficients adjusting for individual characteristics | | | | | | | | | | | | |
| --- | --- | --- | --- | --- | --- | --- | --- | --- | --- | --- | --- | --- | --- | --- |
|  | | | Women | | | Men | |  | | Women | | | Men | |
| Covariates | Mean | | | Standard error | Mean | | Mean | Interaction terms | Mean | | Standard error | Mean | | Standard error |
| White | 0.0000 | | | 0.0000 | 0.0000 | | 0.0000 | Age1*smoke1 | 0.6891 | | 0.035 | 0.9244 | | 0.776 |
| Indian | 0.2672 | | | 0.0537 | 0.2785 | | 0.0425 | Age1*smoke2 | 0.6943 | | 0.066 | 1.9598 | | 3.341 |
| Pakistani | 0.7148 | | | 0.0698 | 0.6068 | | 0.0547 | Age1*smoke3 | -1.6952 | | 0.231 | 2.9994 | | 3.075 |
| Bangladeshi | 0.3703 | | | 0.1073 | 0.7104 | | 0.0727 | Age1*smoke4 | -1.2150 | | 0.308 | 5.0371 | | 3.529 |
| Other Asian | 0.2074 | | | 0.1071 | 0.8626 | | 0.0845 | Age1*AF | -3.5855 | | 0.922 | 8.2354 | | 1.406 |
| Caribbean | -0.1744 | | | 0.0619 | 3.8735 | | 0.0641 | Age1*renal disease | -3.0767 | | 0.528 | -3.9747 | | 3.403 |
| Black African | -0.3272 | | | 0.1275 | 0.1347 | | 0.1094 | Age1*hypertension | -4.0295 | | 0.450 | 7.8738 | | 6.793 |
| Chinese | -0.2201 | | | 0.1721 | -0.1558 | | 0.1538 | Age1*Diabetes | -3.3145 | | 0.369 | 5.0624 | | 2.558 |
| Other | -0.2090 | | | 0.0793 | -3.7728 | | 0.0734 | Age1*BMI 1 | -5.5934 | | 0.617 | 33.5438 | | 0.654 |
| Non-smoker | 0.0000 | | | 0.0000 | 0.1526 | | 0.0000 | Age1*BMI 2 | 64.3636 | | 0.050 | -129.9767 | | 3.584 |
| Former smoker | 0.1947 | | | 0.0152 | 0.0132 | | 0.0108 | Age1*family history CVD | 0.8605 | | 0.050 | 1.9280 | | 3.584 |
| Light smoker | 0.6229 | | | 0.0220 | 0.0644 | | 0.0166 | Age1*SBP | -0.0509 | | 0.003 | 0.0523 | | 0.030 |
| Moderate smoker | 0.7406 | | | 0.0178 | 1.4235 | | 0.0148 | Age1*Townsend | 0.1519 | | 0.007 | -0.1731 | | 0.510 |
| Heavy smoker | 0.9134 | | | 0.0194 | 0.3021 | | 0.0143 | Age2*smoke1 | -0.1765 | | 0.001 | -0.0034 | | 1.594 |
| Age 1 | 3.8735 | | |  | -17.6226 | |  | Age2*smoke2 | -0.2324 | | 0.000 | -0.0051 | | 4.737 |
| Age 2 | 0.1347 | | |  | 0.0242 | |  | Age2*smoke3 | 0.2734 | | 0.002 | 0.0003 | | 4.627 |
| BMI 1* | -0.1558 | | | 0.0423 | 1.7320 | | 0.0299 | Age2*smoke4 | 0.1433 | | 0.003 | 0.0031 | | 5.373 |
| BMI2 * | -3.7728 | | |  | -7.2312 | |  | Age2*AF | 0.4987 | | 0.010 | 0.0073 | | 2.890 |
| Ratio Total / HDL chol | 0.1526 | | | 0.0044 | 0.1751 | | 0.0022 | Age2*renal disease | 0.4393 | | 0.007 | -0.0262 | | 5.654 |
| SBP | 0.0132 | | | 0.0045 | 0.0102 | | 0.0046 | Age2*hypertension | 0.6904 | | 0.005 | 0.0086 | | 3.763 |
| Townsend | 0.0644 | | | 0.0068 | 0.0298 | | 0.0048 | Age2*Diabetes | 0.4865 | | 0.004 | -0.0002 | | 0.193 |
| AF | 1.4235 | | | 0.0310 | 0.9891 | | 0.1018 | Age2*BMI 1 | 1.5223 | | 0.007 | 0.0812 | | 2.110 |
| Rheumatoid arthritis | 0.3021 | | | 0.0319 | 0.2542 | | 0.0445 | Age2*BMI 2 | -12.7413 | |  | -0.2559 | |  |
| Renal disease | 0.8615 | | | 0.0639 | 0.7950 | | 0.0702 | Age2*family history CVD | -0.2757 | | 0.001 | -0.0057 | | 5.321 |
| Hypertension | 0.5889 | | | 0.0115 | 0.6229 | | 0.0112 | Age2*SBP | 0.0074 | | 0.000 | -0.0001 | | 0.058 |
| Diabetes | 1.1350 | | | 0.0199 | 0.9373 | | 0.0175 | Age2*Townsend | -0.0487 | | 0.000 | -0.0011 | | 0.601 |
| Family history of CVD | 0.5134 | | | 0.0122 | 0.5923 | | 0.0111 |  |  | |  |  | |  |
| AF Atrial Fibrillation CVD Cardiovascular disease SBP systolic blood pressure * covariates transformed with fractional polynomials | | | | | | | | | | | | | | |

The QRISK2 risk equation can be used to calculate the probability of a cardiovascular event including: coronary heart disease (angina or myocardial infarction), stroke, or transient ischaemic attacks, fatality due to cardiovascular disease. The equation estimates the probability of a cardiovascular event in the next period conditional on the coefficients listed in Table S16.

The equation for the probability of an event in the next period is calculated as

$$p\left( Y=1 \right)=1-{S(1)}^{\theta}$$

$$\theta=\sum\beta X$$

The probability of an event is calculated from the survival function at 1 year raised to the power of $\theta$, where $\theta$ is the sum product of the coefficients reported in Table S16 multiplied by the individual’s characteristics. Underlying survival curves for men and women were extracted from the QRISK2 open source file. Mean estimates for the continuous variables were also reported in the open source files.

We modified the QRISK2 assumptions regarding the relationship between IGR, diabetes and cardiovascular disease. Firstly, we assumed that individuals with HbA1c>6.5 have an increased risk of cardiovascular disease even if they have not received a formal diagnosis. Secondly, risk of cardiovascular disease was assumed to increase with HbA1c for test results greater than 6.5 to reflect observations from the UKPDS that HbA1c increases the risk of MI and Stroke (23). Thirdly, prior to type 2 diabetes (HbA1c>6.5) HbA1c is linearly associated with cardiovascular disease. A study from the EPIC Cohort has found that a unit increase in HbA1c increases the risk of coronary heart disease by a hazard ratio of 1.25, after adjustment for other risk factors (29). We apply this risk ratio to linearly increase risk above the mean HBA1c observed in the HSE 2011 cohort. A linear risk reduction was applied at HbA1c levels below the HSE mean.

The QRISK2 algorithm identifies which individuals experience a cardiovascular event but does not specify the nature of the event. The nature of the cardiovascular event was determined independently. A targeted search of recent Health Technology appraisals of cardiovascular disease was performed to identify a model for the progression of cardiovascular disease following a first event. A Health Technology Assessment (HTA) assessing statins gives age and sex specific distributions of CVD, which were used to assign all QRISK2 events (12). Table S17 reports the probability of cardiovascular outcomes by age and gender. Stakeholders suggested that there may be different relationships between the risk factors and the different types of CVD (e.g. hypertension is more of a risk factor for stroke). However, we decided not to incorporate these differential factors in evaluating the risk of cardiovascular event types into the model due to a lack of evidence.

Table S17: The probability distribution of cardiovascular events by age and gender

|  | Age | Stable angina | Unstable angina | MI rate | Fatal CHD | TIA | Stroke | Fatal CVD |
| --- | --- | --- | --- | --- | --- | --- | --- | --- |
| Men | 45-54 | 0.307 | 0.107 | 0.295 | 0.071 | 0.060 | 0.129 | 0.030 |
|  | 55-64 | 0.328 | 0.071 | 0.172 | 0.086 | 0.089 | 0.206 | 0.048 |
|  | 65-74 | 0.214 | 0.083 | 0.173 | 0.097 | 0.100 | 0.270 | 0.063 |
|  | 75-84 | 0.191 | 0.081 | 0.161 | 0.063 | 0.080 | 0.343 | 0.080 |
|  | 85+ | 0.214 | 0.096 | 0.186 | 0.055 | 0.016 | 0.351 | 0.082 |
| Women | 45-54 | 0.325 | 0.117 | 0.080 | 0.037 | 0.160 | 0.229 | 0.054 |
|  | 55-64 | 0.346 | 0.073 | 0.092 | 0.039 | 0.095 | 0.288 | 0.067 |
|  | 65-74 | 0.202 | 0.052 | 0.121 | 0.081 | 0.073 | 0.382 | 0.090 |
|  | 75-84 | 0.149 | 0.034 | 0.102 | 0.043 | 0.098 | 0.464 | 0.109 |
|  | 85+ | 0.136 | 0.029 | 0.100 | 0.030 | 0.087 | 0.501 | 0.117 |

### Subsequent Cardiovascular events

After an individual has experienced a cardiovascular event, it is not possible to predict the transition to subsequent cardiovascular events using QRISK2. As with assigning first CVD events, the probability of subsequent events was estimated from the HTA evaluating statins (12). This study reported the probability of future events conditional on the nature of the previous event. Table S18 reports an example of the probabilities within a year of transitioning from stable angina, unstable angina, myocardial infarction (MI), transient ischemic attack (TIA) or stroke for individuals by age group.

Table S18: Probability of cardiovascular event conditional on age and status of previous event (column1)

|  | Stable angina | Unstable angina 1 | Unstable angina 2 | MI 1 | MI 2 | TIA | Stroke 1 | Stroke 2 | CHD death | CVD death |
| --- | --- | --- | --- | --- | --- | --- | --- | --- | --- | --- |
| **Age 45** |  |  |  |  |  |  |  |  |  |  |
| Stable angina | 0.9946 | 0.0013 | 0 | 0.0032 | 0 | 0 | 0 | 0 | 0.0009 | 0 |
| Unstable angina (1^st^ yr) | 0 | 0 | 0.9127 | 0.0495 | 0 | 0 | 0 | 0 | 0.0362 | 0.0016 |
| Unstable angina (subsequent) | 0 | 0 | 0.9729 | 0.0186 | 0 | 0 | 0 | 0 | 0.0081 | 0.0004 |
| MI (1^st^ yr) | 0 | 0 | 0 | 0.128 | 0.8531 | 0 | 0.0015 | 0 | 0.0167 | 0.0007 |
| MI (subsequent) | 0 | 0 | 0 | 0.0162 | 0.978 | 0 | 0.0004 | 0 | 0.0052 | 0.0002 |
| TIA | 0 | 0 | 0 | 0.0016 | 0 | 0.9912 | 0.0035 | 0 | 0.0024 | 0.0013 |
| Stroke (1^st^ yr) | 0 | 0 | 0 | 0.0016 | 0 | 0 | 0.0431 | 0.9461 | 0.0046 | 0.0046 |
| Stroke (subsequent) | 0 | 0 | 0 | 0.0016 | 0 | 0 | 0.0144 | 0.9798 | 0.0021 | 0.0021 |
| **Age 55** |  |  |  |  |  |  |  |  |  |  |
| Stable angina | 0.9874 | 0.0029 | 0 | 0.0062 | 0 | 0 | 0 | 0 | 0.0035 | 0 |
| Unstable angina (1^st^ yr) | 0 | 0 | 0.8859 | 0.0497 | 0 | 0 | 0 | 0 | 0.0617 | 0.0027 |
| Unstable angina (subsequent) | 0 | 0 | 0.9548 | 0.0348 | 0 | 0 | 0 | 0 | 0.01 | 0.0004 |
| MI (1^st^ yr) | 0 | 0 | 0 | 0.1152 | 0.8483 | 0 | 0.0032 | 0 | 0.0319 | 0.0014 |
| MI (subsequent) | 0 | 0 | 0 | 0.0179 | 0.9716 | 0 | 0.001 | 0 | 0.0091 | 0.0004 |
| TIA | 0 | 0 | 0 | 0.0031 | 0 | 0.9626 | 0.0181 | 0 | 0.0092 | 0.007 |
| Stroke (1^st^ yr) | 0 | 0 | 0 | 0.0031 | 0 | 0 | 0.0459 | 0.9288 | 0.0111 | 0.0111 |
| Stroke (subsequent) | 0 | 0 | 0 | 0.0031 | 0 | 0 | 0.0186 | 0.9685 | 0.0049 | 0.0049 |
| **Age 65** |  |  |  |  |  |  |  |  |  |  |
| Stable angina | 0.976 | 0.006 | 0 | 0.011 | 0 | 0 | 0 | 0 | 0.007 | 0 |
| Unstable angina (1^st^ yr) | 0 | 0 | 0.8435 | 0.0488 | 0 | 0 | 0 | 0 | 0.1031 | 0.0046 |
| Unstable angina (subsequent) | 0 | 0 | 0.9244 | 0.0632 | 0 | 0 | 0 | 0 | 0.0119 | 0.0005 |
| MI (1^st^ yr) | 0 | 0 | 0 | 0.1019 | 0.8287 | 0 | 0.0068 | 0 | 0.0599 | 0.0027 |
| MI (subsequent) | 0 | 0 | 0 | 0.0185 | 0.9634 | 0 | 0.0022 | 0 | 0.0152 | 0.0007 |
| TIA | 0 | 0 | 0 | 0.0055 | 0 | 0.9174 | 0.0423 | 0 | 0.0185 | 0.0163 |
| Stroke (1^st^ yr) | 0 | 0 | 0 | 0.0055 | 0 | 0 | 0.0481 | 0.8944 | 0.026 | 0.026 |
| Stroke (subsequent) | 0 | 0 | 0 | 0.0055 | 0 | 0 | 0.0223 | 0.9514 | 0.0104 | 0.0104 |
| **Age 75** |  |  |  |  |  |  |  |  |  |  |
| Stable angina | 0.9681 | 0.0091 | 0 | 0.0158 | 0 | 0 | 0 | 0 | 0.007 | 0 |
| Unstable angina (1^st^ yr) | 0 | 0 | 0.7789 | 0.0466 | 0 | 0 | 0 | 0 | 0.1671 | 0.0074 |
| Unstable angina (subsequent) | 0 | 0 | 0.8733 | 0.1122 | 0 | 0 | 0 | 0 | 0.0139 | 0.0006 |
| MI (1^st^ yr) | 0 | 0 | 0 | 0.0874 | 0.7849 | 0 | 0.0141 | 0 | 0.1088 | 0.0048 |
| MI (subsequent) | 0 | 0 | 0 | 0.0178 | 0.953 | 0 | 0.0047 | 0 | 0.0235 | 0.001 |
| TIA | 0 | 0 | 0 | 0.008 | 0 | 0.8588 | 0.0828 | 0 | 0.0185 | 0.0319 |
| Stroke (1^st^ yr) | 0 | 0 | 0 | 0.008 | 0 | 0 | 0.0446 | 0.8302 | 0.0586 | 0.0586 |
| Stroke (subsequent) | 0 | 0 | 0 | 0.008 | 0 | 0 | 0.0246 | 0.9262 | 0.0206 | 0.0206 |
| **Age 85** |  |  |  |  |  |  |  |  |  |  |
| Stable angina | 0.9601 | 0.0122 | 0 | 0.0207 | 0 | 0 | 0 | 0 | 0.007 | 0 |
| Unstable angina (1^st^ yr) | 0 | 0 | 0.6873 | 0.0425 | 0 | 0 | 0 | 0 | 0.2587 | 0.0115 |
| Unstable angina (subsequent) | 0 | 0 | 0.7878 | 0.1955 | 0 | 0 | 0 | 0 | 0.016 | 0.0007 |
| MI (1^st^ yr) | 0 | 0 | 0 | 0.0711 | 0.7053 | 0 | 0.0278 | 0 | 0.1875 | 0.0083 |
| MI (subsequent) | 0 | 0 | 0 | 0.016 | 0.9394 | 0 | 0.0091 | 0 | 0.034 | 0.0015 |
| TIA | 0 | 0 | 0 | 0.0104 | 0 | 0.838 | 0.0961 | 0 | 0.0185 | 0.037 |
| Stroke (1^st^ yr) | 0 | 0 | 0 | 0.0104 | 0 | 0 | 0.0446 | 0.702 | 0.1215 | 0.1215 |
| Stroke (subsequent) | 0 | 0 | 0 | 0.0104 | 0 | 0 | 0.0252 | 0.8894 | 0.0375 | 0.0375 |

### Congestive Heart Failure

The review of previous economic evaluations of diabetes prevention cost-effectiveness studies found that only a small number of models had included congestive heart failure as a separate outcome. Discussion with the stakeholder group identified that the UKPDS Outcomes model would be an appropriate risk model for congestive heart failure in type 2 diabetes patients. However, it was suggested that this would not be an appropriate risk equation for individuals with normal glucose tolerance or impaired glucose tolerance. The Framingham risk equation was suggested as an alternative. As described above, switching from the framgingam risk score to the UKPDS was not possible due to differences in covariate selection. The main limitations of this equation is that it is quite old, based on a non-UK population, and include diabetes as a discrete health state rather than on a continuous scale.

Congestive heart failure was included as a separate cardiovascular event because it was not included as an outcome of the QRISK2. The Framingham Heart Study has reported logistic regressions to estimate the 4 year probability of congestive heart failure for men and women (30). The equations included age, diabetes diagnosis, BMI and systolic blood pressure to adjust risk based on individual characteristics. We used this risk equation to estimate the probability of congestive heart failure in the SPHR diabetes prevention model. Table S19 describes the covariates for the logit models to estimate the probability of congestive heart failure in men and women.

Table S19: Logistic regression coefficients to estimate the 4-year probability of congestive heart failure from the Framingham study

| Variables | Units | Regression  Coefficient | OR (95% CI) | P |
| --- | --- | --- | --- | --- |
| Men | | | | |
| Intercept |  | -9.2087 |  |  |
| Age | 10 y | 0.0412 | 1.51 (1.31-1.74) | <.001 |
| Left ventricular hypertrophy | Yes/no | 0.9026 | 2.47 (1.31-3.77) | <.001 |
| Heart rate | 10 bpm | 0.0166 | 1.18 (1.08-1.29) | <.001 |
| Systolic blood pressure | 20 mm Hg | 0.00804 | 1.17 (1.04-1.32) | 0.007 |
| Congenital heart disease | Yes/no | 1.6079 | 4.99 (3.80-6.55) | <.001 |
| Valve disease | Yes/no | 0.9714 | 2.64 (1.89-3.69) | <.001 |
| Diabetes | Yes/no | 0.2244 | 1.25 (0.89-1.76) | 0.2 |
| Women | | | | |
| Intercept |  | -10.7988 |  |  |
| Age | 10 y | 0.0503 | 1.65 (1.42-1.93) | <.001 |
| left ventricular hypertrophy | Yes/no | 1.3402 | 3.82 (2.50-5.83) | <.001 |
| Heart rate | 100 cL | 0.0105 | 1.11 (1.01-1.23) | 0.03 |
| Systolic blood pressure | 10 bpm | 0.00337 | 1.07 (0.96-1.20) | 0.24 |
| congenital heart disease | 20 mm Hg | 1.5549 | 4.74 (3.49-6.42) | <.001 |
| Valve disease | Yes/no | 1.3929 | 4.03 (2.86-5.67) | <.001 |
| Diabetes | Yes/no | 1.3857 | 4.00 (2.78-5.74) | <.001 |
| BMI | kg/m2 | 0.0578 | 1.06 (1.03-1.09) | <.001 |
| Valve disease and diabetes | Yes/no | -0.986 | 0.37 (0.18-0.78) | 0.009 |
| *OR indicates odds ratio; CI, confidence interval; LVH, left ventricular hypertrophy; CHD, congenital heart disease; and BMI, body mass index. Predicted probability of heart failure can be calculated as: p = 1/(1+exp(-xbeta)), where xbeta = Intercept + Sum (of regression coefficient*value of risk factor) | | | | |

Many of the risk factors included in this risk equation were not simulated in the diabetes model, therefore they could not be included in the model to predict CHD. We adjusted the baseline odds of CHD to reflect the expected prevalence of these symptoms in a UK population.

The proportion of the UK population with left ventricular hypertrophy was assumed to be 5% in line with previous analyses of the Whitehall II cohort (31). The heart rate for men was assumed to be 63.0bpm and for women 65.6bpm based on data from previous Whitehall II cohort analyses (32). The prevalence of congenital heart disease was estimated from an epidemiology study in the North of England. The study reports the prevalence of congenital heart disease among live births which was used to estimate the adult prevalence (33). This may over-estimate the prevalence, because the life expectancy of births with congenital heart disease is reduced compared with the general population. However, given the low prevalence it is unlikely to impact on the results. The prevalence of valve disease was estimated from the Echocardiographic Heart of England Screening study (34).

Using the estimated population values we adjusted the intercept values to account for the population risk in men and women. This resulted in a risk equation with age, systolic blood pressure, diabetes (diabetes diagnosis or HbA1c>6.5), and BMI in women to describe the risk of congestive heart failure for the policy analysis model.

## Microvascular Complications

The review of previous economic evaluations identified that the UKPDS data was commonly used to estimate the incidence of microvascular complications (22). This data has the advantage of being estimated from a UK diabetic population. Given that the events described in the UKPDS outcomes model are indicative of late stage microvascular complications, we did not believe it was necessary to seek an alternative model that would be representative of an impaired glucose tolerance population.

We adopted a simple approach to modelling microvascular complications. We used both versions of the UKPDS Outcomes model to estimate the occurrence of major events relating to these complications, including renal failure, amputation, foot ulcer, and blindness (23). These have the greatest cost and utility impact compared with earlier stages of microvascular complications, so are more likely to have an impact on the SPHR diabetes prevention outcomes.

As a consequence, we assumed that microvascular complications only occur in individuals with HbA1c>48 mmol/mol (6.5%). Whilst some individuals with hyperglycaemia (HbA1c>42 mmol/mol [6.0%]) may be at risk of developing microvascular complications, it is unlikely that they will progress to renal failure, amputation or blindness before a diagnosis of diabetes. Importantly, we did not assume that only individuals who have a formal diagnosis of diabetes are at risk of these complications. This allows us to incorporate the costs of undetected diabetes into the simulation.

The UKPDS includes four statistical models to predict foot ulcers, amputation with no prior ulcer, amputation with prior ulcer and a second amputation (23). In order to simplify the simulation of neuropathy outcomes we consolidated the models for first amputation with and without prior ulcer into a single equation. The parametric survival models were used to generate estimates of the cumulative hazard in the current and previous period. From which the probability of organ damage being diagnosed was estimated.

|  | $p\left( Death \right)=1-exp(-\left( H\left( t \right)-H\left( t-1 \right) \right))$ |  |
| --- | --- | --- |

The functional form for the microvascular models included exponential and Weibull.

#### Retinopathy

We used the UKPDS outcomes model v2 to estimate the incidence of blindness in individuals with HbA1c>48 mmol/mol (6.5%) (23). The exponential model assumes a baseline hazard $\lambda$, which can be calculated from the model coefficients reported in Table S20 and the individual characteristics for $\boldsymbol{X}$.

$$\lambda=exp\left( \beta_{0}\mathbf{+}\boldsymbol{X}\boldsymbol{\beta}_{\boldsymbol{k}} \right)$$

Table S20: Parameters of the UKPDS2 Exponential Blindness survival model

|  | Mean coefficient | Standard error | Modified mean coefficient |
| --- | --- | --- | --- |
| Lambda | -11.607 | 0.759 | -10.967 |
| Age at diagnosis | 0.047 | 0.009 | 0.047 |
| HbA1c | 0.171 | 0.032 | 0.171 |
| Heart rate | 0.080 | 0.039 |  |
| SBP | 0.068 | 0.032 | 0.068 |
| White Blood Count | 0.052 | 0.019 |  |
| CHF History | 0.841 | 0.287 | 0.841 |
| IHD History | 0.0610 | 0.208 | 0.061 |
| SBP Systolic Blood Pressure; CHF Congestive Heart Failure; IHD Ischaemic Heart Disease | | | |

The age at diagnosis coefficient was multiplied by age in the current year if the individual had not been diagnosed with diabetes, and by the age at diagnosis if the individual had received a diagnosis.

The expected values for the risk factors not included in the SPHR model (heart rate and white blood count) were taken from Figure 3 of the UKPDS publication in which these are described (23). Assuming these mean values, it was possible to modify the baseline risk without simulating heart rate and white blood cell count.

#### Neuropathy

We used the UKPDS outcomes model v2 to estimate the incidence of ulcer and amputation in individuals with HbA1c>48 mmol/mol (6.5%) (23). The parameters of the ulcer and first amputation models are reported in Table S21.

Table S21: Parameters of the UKPDS2 Exponential model for Ulcer, Weibull model for first amputation with no prior ulcer and exponential model for 1^st^ amputation with prior ulcer

|  | Ulcer | | 1^st^ Amputation no prior ulcer | | 1^st^ Amputation prior ulcer | | 2^nd^ Amputation | |
| --- | --- | --- | --- | --- | --- | --- | --- | --- |
|  | Logistic | | Weibull | | Exponential | | Exponential | |
|  | Mean | Standard error | Mean | Standard error | Mean | Standard error | Mean | Standard error |
| Lambda | -11.295 | 1.130 | -14.844 | 1.205 | -0.881 | 1.39 | -3.455 | 0.565 |
| Rho |  |  | 2.067 | 0.193 |  |  |  |  |
| Age at diagnosis | 0.043 | 0.014 | 0.023 | 0.011 | -0.065 | 0.027 |  |  |
| Female | -0.962 | 0.255 | -0.0445 | 0.189 |  |  |  |  |
| Atrial fibrillation |  |  | 1.088 | 0.398 |  |  |  |  |
| BMI | 0.053 | 0.019 |  |  |  |  |  |  |
| HbA1c | 0.160 | 0.056 | 0.248 | 0.042 |  |  | 0.127 | 0.06 |
| HDL |  |  | -0.059 | 0.032 |  |  |  |  |
| Heart rate |  |  | 0.098 | 0.050 |  |  |  |  |
| MMALB |  |  | 0.602 | 0.180 |  |  |  |  |
| PVD | 0.968 | 0.258 | 1.010 | 0.189 | 1.769 | 0.449 |  |  |
| SBP |  |  | 0.086 | 0.043 |  |  |  |  |
| WBC |  |  | 0.040 | 0.017 |  |  |  |  |
| Stroke History |  |  | 1.299 | 0.245 |  |  |  |  |

The exponential model assumes a baseline hazard $\lambda$, which can be calculated from the model coefficients reported in Table 15 and the individual characteristics for $\boldsymbol{X}$.

$$\lambda=exp\left( \beta_{0}\mathbf{+}\boldsymbol{X\beta} \right)$$

The Weibull model for amputation assumes a baseline hazard:

$$h\left( t \right)=\rho t^{\rho-1}exp(\lambda)$$

where $\lambda$is also conditional on the coefficients and individual characteristics at time t.

The logistic model for ulcer is described below.

$$\Pr\left( y=1 | \mathbf{X} \right)=\frac{exp(\boldsymbol{X\beta})}{1+exp(\boldsymbol{X\beta}))}$$

The ulcer and amputation models include a number of covariates that were not included in the simulation. As such it was necessary to adjust the statistical models to account for these measures. We estimated a value for the missing covariates and added the value multiplied by the coefficient to the baseline hazard.

The expected values for the risk factors not included in the SPHR model (heart rate, white blood count, micro-/macroalbuminurea, peripheral vascular disease and atrial fibrillation) were taken from Figure 3 of the UKPDS publication in which these are described (23). In the ulcer model we assumed that 2% of the population had peripheral vascular disease.

The amputation risk model with a history of ulcer was not included in the simulation, but was used to estimate an additional log hazard ratio to append onto the amputation model without a history of ulcer. The log hazard was estimated for each model assuming the same values for other covariates. The difference in the log hazard between the two models was used to approximate the log hazard ratio for a history of ulcer in the amputation model (10.241). The final model specifications are reported in Table S22.

Table S22: Coefficients estimates for Ulcer and 1^st^ Amputation

|  | Ulcer | | 1^st^ Amputation | | 2^nd^ Amputation | |
| --- | --- | --- | --- | --- | --- | --- |
|  | Logistic | | Weibull | | Exponential | |
|  | Mean | Standard error | Mean | Standard error | Mean | Standard error |
| Lambda | -11.276 | 1.13 | -13.954 | 1.205 | -3.455 | 0.565 |
| Rho |  |  | 2.067 | 0.193 |  |  |
| Age at Diagnosis | 0.043 | 0.014 | 0.023 | 0.011 |  |  |
| Female | -0.962 | 0.255 | -0.445 | 0.189 |  |  |
| BMI | 0.053 | 0.019 |  |  |  |  |
| HbA1c | 0.160 | 0056 | 0.248 | 0.042 | 0.127 | 0.06 |
| HDL |  |  | -0.059 | 0.032 |  |  |
| Stroke |  |  | 1.299 | 0.245 |  |  |
| Foot Ulcer |  |  | 10.241 |  |  |  |

#### Nephropathy

We used the UKPDS outcomes model v1 to estimate the incidence of renal failure in individuals with HbA1c>48 mmol/mol (6.5%) (16). Early validation analyses identified that the UKPDS v2 model substantially overestimated the incidence of renal failure in the SPHR model. The Weibull model for renal failure assumes a baseline hazard:

$$h\left( t \right)=\rho t^{\rho-1}exp(\lambda)$$

where $\lambda$is also conditional on the coefficients and individual characteristics at time t. The parameters of the renal failure risk model are reported in Table S23.

Table S23: Parameters of the UKPDS2 Weibull renal failure survival model

|  | Mean | Standard error |
| --- | --- | --- |
| Lambda | -10.016 | 0.939 |
| Shape parameter | 1.865 | 0.387 |
| SBP | 0.404 | 0.106 |
| BLIND History | 2.082 | 0.551 |

##

## Cancer

The conceptual model identified breast cancer and colorectal cancer risk as being related to BMI. However, these outcomes were not frequently included in previous cost-effectiveness models for diabetes prevention. Discussion with stakeholders identified the EPIC Norfolk epidemiology cohort study as a key source of information about cancer risk in a UK population. Therefore, we searched publications from this cohort to identify studies reporting the incidence of these risks. In order to obtain the best quality evidence for the relationship between BMI and cancer risk we searched for a recent systematic review and meta-analysis using key terms ‘Body Mass Index’ and ‘Cancer’, filtering for meta-analysis studies.

### Breast cancer

Incidence rates for breast cancer in the UK were estimated from the European Prospective Investigation of Cancer (EPIC) cohort. This is a large multi-centre cohort study looking at diet and cancer. In 2004 the UK incidence of breast cancer by menopausal status was reported in a paper from this study investigating the relationship between body size and breast cancer (35). The estimates of the breast cancer incidence in the UK are reported in Table S24, in the simulation we assume the risk of breast cancer starts from age 50.

Table S24: UK breast cancer incidence

|  | Number of Cases | Person Years | Mean BMI | Incidence Rate of per person-year | Standard error | Reference |
| --- | --- | --- | --- | --- | --- | --- |
| UK pre-menopause | 102 | 103114.6 | 24 | 0.00099 | 0.00009 | (35) |
| UK post-menopause | 238 | 84214.6 | 24 | 0.00283 | 0.00004 | (35) |

A large meta-analysis that included 221 prospective observational studies has reported relative risks of cancers per unit increase in BMI, including breast cancer by menopausal status (36). We included a risk adjustment in the model so that individuals with higher BMI have a higher probability of pre-and post-menopausal breast cancer (36). In the simulation we adjusted the probability of breast cancer according to the difference in the individual’s BMI and the average BMI reported in the EPIC cohort. The relative risk and confidence intervals per 5mg/m^2^ increase in BMI are reported in Table S25.

Table S25: Relative risk of Breast cancer by BMI

|  | Mean Relative risk | 2.5^th^ Confidence Interval | 97.5^th^ Confidence Interval | Reference |
| --- | --- | --- | --- | --- |
| UK pre-menopause | 0.89 | 0.84 | 0.94 | (36) |
| UK post-menopause | 1.09 | 1.04 | 1.14 | (36) |

### Colorectal cancer

Incidence rates for colorectal cancer in the UK were reported from the European Prospective Investigation of Cancer (EPIC) cohort. The UK incidence of colorectal cancer is reported by gender in a paper from this study investigating the relationship between body size and colon and rectal cancer (37). The estimates of the colorectal cancer incidence are reported in Table S26, in the simulation we assume the risk of colorectal cancer starts from age 50.

Table S26: UK colorectal cancer incidence

|  | Number of Cases | Person Years | Mean Age | Mean BMI | Incidence Rate of per person-year | Standard error | Reference |
| --- | --- | --- | --- | --- | --- | --- | --- |
| Male | 125 | 118468 | 53.1 | 25.4 | 0.00106 | 0.0001 | (37) |
| Female | 145 | 277133 | 47.7 | 24.5 | 0.00052 | 0.0002 | (37) |

The risk of colorectal cancer has been linked to obesity. We included a risk adjustment in the model to reflect observations that the incidence of breast cancer is increased in individuals with higher BMI. A large meta-analysis that included 221 prospective observational studies has reported relative risks of BMI and cancers, including colon cancer by gender (36). We selected linear relative risk estimates estimated from pooled European and Australian populations. In the simulation we adjusted the incidence of colorectal cancer by adjusting the probability of colorectal cancer by the difference in the individual’s BMI and the average BMI reported in the EPIC cohort. The relative risk and confidence intervals per 5mg/m^2^ increase in BMI are reported in Table S27.

Table S27: Relative risk of colon cancer by BMI

|  | Mean Relative risk | 2.5^th^ Confidence Interval | 97.5^th^ Confidence Interval | Reference |
| --- | --- | --- | --- | --- |
| Male | 1.21 | 1.18 | 1.24 | (36) |
| Female | 1.04 | 1.00 | 1.07 | (36) |

##

## Osteoarthritis

Stakeholders suggested that diabetes and BMI should be included as independent risk factors for osteoarthritis. Osteoarthritis had not been included as a health state in previous cost-effectiveness models. The stakeholder group requested that BMI and diabetes be included as risk factors for osteoarthritis based on recent evidence (38). A search for studies using key words ‘Diabetes’, ‘Osteoarthritis’ and ‘Cohort Studies’ did not identify a UK based study with diabetes and body mass index included as independent covariates in the risk model. Therefore, the Italian study was used in the model.

A study from the Bruneck cohort, a longitudinal study of inhabitants of a town in Italy reported diabetes and BMI as independent risk factors for osteoarthritis (38).

The cohort may not be representative of a UK cohort. However, the individuals are from a European country, the study has a large sample size and has estimated the independent effects of BMI and diabetes on the risk of osteoarthritis. No UK based studies identified in our searches met these requirements. The data used to estimate the incidence of osteoarthritis in adults over the age of 45 is reported in Table S28. We did not identify any studies that described diabetes risk on a continuous scale.

Table S28: Incidence of osteoarthritis and estimated risk factors

|  | No cases | Person years | Mean BMI | Incidence rate | Standard error | Reference |
| --- | --- | --- | --- | --- | --- | --- |
| No diabetes | 73 | 13835 | 24.8 | 0.0053 | 0.0006 | (38) |
|  | Hazard ratio | 2.5th | 97.5th |  |  | Reference |
| HR Diabetes | 2.06 | 1.11 | 3.84 |  |  | (38) |
| HR BMI | 1.076 | 1.023 | 1.133 |  |  | (38) Personal communication |

## Depression

Depression was not included as a health state in previous cost-effectiveness models for diabetes prevention. However, a member of the stakeholder group identified that a relationship between diabetes and depression was included in the CORE diabetes treatment model (39). Therefore, the references used in this model were used.

Depression was included as a health state in the model. However, the severity of depression was not modelled. Some individuals enter the simulation with depression at baseline according to individual responses in the Health Survey for England 2014 questionnaire. Depression is described in the simulation as a chronic state from which individuals do not completely remit. We did not estimate the effect of depression on the longitudinal changes for BMI, glycaemia, SBP and cholesterol. As a consequence, it was not possible to relate the impact of depression to the incidence of diabetes and cardiovascular risk.

In the simulation, individuals can develop depression in any cycle of the model. The baseline incidence of depression among all individuals without a history of depression was estimated from a study examining the bidirectional association between depressive symptoms and type 2 diabetes (40). Although the study was not from a UK population, the US cohort included ethnically diverse men and women aged 45 to 84 years. We assumed that diagnosis of diabetes and/or CVD increased the incidence of depression in individuals who do not have depression at baseline. We identified a method for inflating risk of depression for individuals with diabetes from the US cohort study described above (40). The risk of depression in individuals who have had a stroke was also inflated according to a US cohort study (41). Odds of depression and odds ratios for inflated risk of depression due to diabetes or stroke are presented in Table S29.

Table S29: Baseline incidence of depression

| Baseline Risk of depression | | | |
| --- | --- | --- | --- |
|  | Mean | Standard error |  |
| Depression cases in NGT | 336 |  |  |
| Person years | 9139 |  |  |
| Odds of depression | 0.0382 | 0.002 |  |
| Log odds of depression | -3.266 |  |  |
| Inflated risk for Diabetes | | | |
|  | Mean | 2.5th CI | 97.5th CI |
| Odds ratio of diabetes | 1.52 | 1.09 | 2.12 |
| Log odds ratio of diabetes | 0.419 |  |  |
| Inflate risk of stroke | | | |
| Odds ratio of stroke | 6.3 | 1.7 | 23.2 |
| Log odds ratio stroke | 1.8406 |  |  |
| NGT Normal Glucose Tolerance | | | |

## Dementia

The risk dementia diagnosis is estimated from risk models estimated from the THIN database (42). The THIN dementia risk score uses data from The Health Improvement Network (THIN) database from across the UK. Routinely collected data was used to predict 5-year risk of recorded diagnosis of Dementia for those aged 60-79 and 80+. The sample size is large and the risk scores are representative of the United Kingdom and diagnosis practices between 2000-2011. The disadvantage of these risk scores are the relatively short follow-up of patients, the low predictive power of the older risk score, and narrow scope to predict dementia diagnosis but not dementia onset.

The parameters for the THIN 60-79 year old and 80-99 risk models are reported in Table S30.

Table S30: THIN dementia risk models

| THIN 60-79 Risk Score | | | THIN 80-99 Risk Score | | |
| --- | --- | --- | --- | --- | --- |
| Parameter label | mean | Standard error | Parameter label | mean | Standard error |
| Baseline hazard | 0.9969 |  | Baseline hazard | -0.9277 |  |
| Age | 0.2092 | 0.0047 | Age | 0.055 | 0.0041 |
| Age^2^ | -0.0034 | 0.0003 | Age^2^ | -0.005 | 0.0010 |
| Female | 0.1285 | 0.0278 | Female | 0.16 | 0.0286 |
| Calendar Year | 0.0448 | 0.0050 | Calendar Year | 0.074 | 0.0056 |
| Townsend quintile 2 | 0.0134 | 0.0390 | BMI | -0.05 | 0.0066 |
| Townsend quintile 3 | 0.1179 | 0.0392 | Anti-hypertensives | -0.249 | 0.0265 |
| Townsend quintile 4 | 0.2018 | 0.0402 | Systolic Blood Pressure | -0.006 | 0.0010 |
| Townsend quintile 5 | 0.2255 | 0.0447 | Lipid ratio | 0.042 | 0.0495 |
| BMI | -0.0616 | 0.0038 | Past Smoker | -0.178 | 0.0281 |
| BMI^2^ | 0.0025 | 0.0003 | Smoker | -0.134 | 0.0485 |
| Anti-hypertensives | -0.1320 | 0.0296 | Alcohol Porblems | 0.256 | 0.1352 |
| Past Smoker | -0.0679 | 0.0301 | Diabetes | 0.183 | 0.0413 |
| Smoker | -0.0866 | 0.0415 | Stroke | 0.242 | 0.0332 |
| Alcohol problems | 0.4435 | 0.0799 | Atrial Fibrillation | 0.057 | 0.0383 |
| Diabetes | 0.2867 | 0.0417 | Depression | 0.4 | 0.0332 |
| Depression | 0.8336 | 0.0325 | Anxiety | 0.136 | 0.0520 |
| Stroke | 0.5772 | 0.0394 | NSAIDs use | -0.157 | 0.0408 |
| Atrial Fibrillation | 0.2207 | 0.0514 | Aspirin use | 0.092 | 0.0281 |
| Aspirin use | 0.2528 | 0.0326 |  |  |  |

For the SPHR prevention model the 5-year Dementia risk was transformed into 1 year individual probabilities. The Dementia risk scores include fixed and time-varying patient characteristics. As a consequence, it is not possible to use standard methods of transforming probabilities over different time-horizons (43). We used a simple calibration technique to modify the baseline hazard to reflect simulated changes in the populations risk profile over 5 years. We calibrate the simulated 5 year incidence of dementia against the predicted incidence for each age group in the THIN database .

For each risk model we simulated 20,000 randomly sampled patients aged 60-79 and 80-95 in 50 model runs. For each sample we repeated simulations multiple times, in each simulation the baseline hazard was adjusted until the incidence of Dementia equalled the THIN risk score prediction based on baseline characteristics. The baseline hazard adjustment was estimated by averaging the adjustments needed for each of the 20 simulation runs to match the THIN prediction. The calibration was designed to calibrate to the predicted incidence, rather than the reported incidence from the THIN dataset to account for any differences in the baseline characteristics of the THIN data and HSE sample. For example, the mean age for the development cohort of the 60-79 model were 65.6, whereas the mean simulated ages was 70. Age is an important predictor of Dementia incidence so it is important to adjust for differences in baseline age between the observed and simulated data.

The THIN database reports a crude incidence of 1.88 per 1,000 persons years for 60-79 and 16.53 per 1,000 person years for ages 80-99 (column 2 Table S31). The 5 year risk score for individuals sampled from the Health Survey for England reports a 5 year incidence of 0.00255 and 0.01523 (column 3 Table S31). Using the adjustment factor identified by calibration (column 5 Table S31), we simulated a 5 year incidence of 0.00255 and 0.01510 (column 4 Table S31). The adjustment factor is applied to the baseline hazards of the THIN dementia risk scores.

Table S31: Dementia incidence rates used to derive the adjustment factor

|  | 5 year crude incidence from QResearch | 5 year risk score (SD) for Health Survey for England population | 5 year simulated incidence (SD) with adjustment factor | Adjustment factor (SE) |
| --- | --- | --- | --- | --- |
| THIN risk model 60-79 | 0.00188 | 0.00255 | 0.00255 | 7.628 (0.104) |
| THIN risk model 80+ | 0.01653 | 0.01523 | 0.01510 | 4.557 (0.020) |

### Dementia Diagnosis

A Swedish registry reporting MMSE scores at dementia diagnosis was identified (44). This study reported a mean MMSE score at diagnosis of 21.2 (SD 5.2). This data was used to generate MMSE scores at diagnosis in the model because it reflects cognitive function in a cohort diagnosed in routine care. In order to generate heterogeneity in cognitive function at diagnosis a Gamma distribution was fitted to this mean and standard distribution and patients MMSE score at diagnosed was sampled from this distribution. Sampled value outside the limits of the MMSE score, assuming a maximum score at diagnosis of 25, were re-sampled from a uniform distribution within these limits. The resulting distribution was compared against summary data from CFAS for an incident cohort to validate the simulated MMSE scores against this dataset (45).


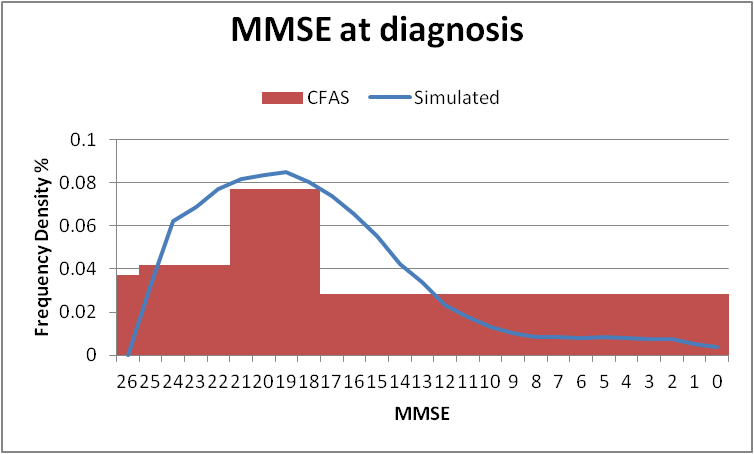


### Disease Progression

Dementia disease progression was characterized by a deterioration in MMSE score. This relatively simple characterization of the disease is sufficient to capture major cost escalations and quality of life deterioration. A more complex structure that explicity models insitutionalisation was considered (46). However, it was concluded that the data on insitutionalisation was out of date. Therefore, the modelling structure was aligned to the most up to date cost estimates for dementia (47).

Changes in MMSE score over time were estimated using data from a recent cost-effectiveness model for Donepezil (48). Although the data are from Canada and are relatively old, the sample size is large and model specification allows for detailed characterization of MMSE decline. The regression allows rate of change in MMSE to be conditional on age at baseline and includes splines to describe a different rate of change at different levels of MMSE. For example, the decline in MMSE slows as the score declines below 9.

## Mortality

### Cardiovascular Mortality

Cardiovascular mortality is included as an event within the QRISK2 (25) and the probability of subsequent cardiovascular events obtained from an HTA assessing statins (12), as described in the Cardiovascular disease section above.

### Cancer Mortality

Cancer mortality rates were obtained from the Office of National statistics (49). The ONS report one and five year net survival rates for various cancer types, by age group and gender. Net survival was an estimate of the probability of survival from the cancer alone. It can be interpreted as the survival of cancer patients after taking into account the background mortality that the patients would have experienced if they had not had cancer.

The age-adjusted 5-year survival rate for breast cancer and colorectal cancer were used to estimate an annual risk of mortality assuming a constant rate of mortality. We assume that the mortality rate does not increase due to cancer beyond 5 years after cancer diagnosis. The five year survival rate for breast cancer is 84.3%, which translated into a 3.37% annual probability of death from breast cancer. The five year survival rate for persons with colorectal cancer is 55.3%, which translated into a 11.16% annual probability of death from colorectal cancer.

### Other cause Mortality (including diabetes and Dementia risk)

Other cause mortality describes the risk of death from any cause except CVD, and cancer. All-cause mortality rates by age and sex were extracted from the 2014 Office of National Statistics life tables (5;7). The mortality statistics report the number of deaths by ICD codes for 5-year age groups. We subtracted the number of cardiovascular disease,diabetes, dementia, breast and colorectal cancer related deaths from the all-cause mortality total to estimate other cause mortality rates by age and sex (Table S32).

Table S32: All cause and derived other cause mortality from the Office of National statistics

|  | All cause | All cause | Other cause | Other cause |  | All cause | All cause | Other cause | Other cause |
| --- | --- | --- | --- | --- | --- | --- | --- | --- | --- |
|  | Men | Women | Men | Women |  | Men | Women | Men | Women |
| 1 | 0.0003 | 0.0003 | 0.0003 | 0.0003 | 51 | 0.0030 | 0.0021 | 0.0022 | 0.0015 |
| 2 | 0.0002 | 0.0001 | 0.0002 | 0.0001 | 52 | 0.0030 | 0.0021 | 0.0022 | 0.0015 |
| 3 | 0.0001 | 0.0001 | 0.0001 | 0.0001 | 53 | 0.0030 | 0.0021 | 0.0022 | 0.0015 |
| 4 | 0.0001 | 0.0001 | 0.0001 | 0.0001 | 54 | 0.0030 | 0.0021 | 0.0022 | 0.0015 |
| 5 | 0.0001 | 0.0001 | 0.0001 | 0.0001 | 55 | 0.0030 | 0.0021 | 0.0022 | 0.0015 |
| 6 | 0.0001 | 0.0001 | 0.0001 | 0.0001 | 56 | 0.0030 | 0.0021 | 0.0022 | 0.0015 |
| 7 | 0.0001 | 0.0001 | 0.0001 | 0.0001 | 57 | 0.0030 | 0.0021 | 0.0022 | 0.0015 |
| 8 | 0.0001 | 0.0001 | 0.0001 | 0.0001 | 58 | 0.0030 | 0.0021 | 0.0022 | 0.0015 |
| 9 | 0.0001 | 0.0001 | 0.0001 | 0.0001 | 59 | 0.0030 | 0.0021 | 0.0022 | 0.0015 |
| 10 | 0.0001 | 0.0001 | 0.0001 | 0.0001 | 60 | 0.0030 | 0.0021 | 0.0022 | 0.0015 |
| 11 | 0.0001 | 0.0001 | 0.0001 | 0.0001 | 61 | 0.0030 | 0.0021 | 0.0022 | 0.0015 |
| 12 | 0.0001 | 0.0001 | 0.0001 | 0.0001 | 62 | 0.0030 | 0.0021 | 0.0022 | 0.0015 |
| 13 | 0.0001 | 0.0001 | 0.0001 | 0.0001 | 63 | 0.0030 | 0.0021 | 0.0022 | 0.0015 |
| 14 | 0.0001 | 0.0001 | 0.0001 | 0.0001 | 64 | 0.0030 | 0.0021 | 0.0022 | 0.0015 |
| 15 | 0.0001 | 0.0001 | 0.0001 | 0.0001 | 65 | 0.0030 | 0.0021 | 0.0022 | 0.0015 |
| 16 | 0.0002 | 0.0001 | 0.0002 | 0.0001 | 66 | 0.0030 | 0.0021 | 0.0022 | 0.0015 |
| 17 | 0.0003 | 0.0001 | 0.0003 | 0.0001 | 67 | 0.0030 | 0.0021 | 0.0022 | 0.0015 |
| 18 | 0.0004 | 0.0002 | 0.0004 | 0.0002 | 68 | 0.0030 | 0.0021 | 0.0022 | 0.0015 |
| 19 | 0.0005 | 0.0002 | 0.0004 | 0.0002 | 69 | 0.0030 | 0.0021 | 0.0022 | 0.0015 |
| 20 | 0.0004 | 0.0002 | 0.0004 | 0.0002 | 70 | 0.0030 | 0.0021 | 0.0022 | 0.0015 |
| 21 | 0.0004 | 0.0002 | 0.0004 | 0.0002 | 71 | 0.0030 | 0.0021 | 0.0022 | 0.0015 |
| 22 | 0.0004 | 0.0002 | 0.0004 | 0.0002 | 72 | 0.0030 | 0.0021 | 0.0022 | 0.0015 |
| 23 | 0.0005 | 0.0002 | 0.0005 | 0.0002 | 73 | 0.0030 | 0.0021 | 0.0022 | 0.0015 |
| 24 | 0.0005 | 0.0002 | 0.0005 | 0.0002 | 74 | 0.0030 | 0.0021 | 0.0022 | 0.0015 |
| 25 | 0.0005 | 0.0002 | 0.0005 | 0.0002 | 75 | 0.0030 | 0.0021 | 0.0022 | 0.0015 |
| 26 | 0.0006 | 0.0002 | 0.0006 | 0.0002 | 76 | 0.0030 | 0.0021 | 0.0022 | 0.0015 |
| 27 | 0.0006 | 0.0003 | 0.0006 | 0.0002 | 77 | 0.0030 | 0.0021 | 0.0022 | 0.0015 |
| 28 | 0.0006 | 0.0003 | 0.0006 | 0.0003 | 78 | 0.0030 | 0.0021 | 0.0022 | 0.0015 |
| 29 | 0.0006 | 0.0003 | 0.0006 | 0.0003 | 79 | 0.0030 | 0.0021 | 0.0022 | 0.0015 |
| 30 | 0.0007 | 0.0003 | 0.0006 | 0.0003 | 80 | 0.0030 | 0.0021 | 0.0022 | 0.0015 |
| 31 | 0.0007 | 0.0004 | 0.0007 | 0.0003 | 81 | 0.0030 | 0.0021 | 0.0022 | 0.0015 |
| 32 | 0.0007 | 0.0004 | 0.0007 | 0.0003 | 82 | 0.0030 | 0.0021 | 0.0022 | 0.0015 |
| 33 | 0.0008 | 0.0005 | 0.0007 | 0.0004 | 83 | 0.0030 | 0.0021 | 0.0022 | 0.0015 |
| 34 | 0.0008 | 0.0005 | 0.0008 | 0.0004 | 84 | 0.0030 | 0.0021 | 0.0022 | 0.0015 |
| 35 | 0.0010 | 0.0005 | 0.0009 | 0.0004 | 85 | 0.0030 | 0.0021 | 0.0022 | 0.0015 |
| 36 | 0.0010 | 0.0006 | 0.0009 | 0.0005 | 86 | 0.0030 | 0.0021 | 0.0022 | 0.0015 |
| 37 | 0.0011 | 0.0006 | 0.0010 | 0.0005 | 87 | 0.0030 | 0.0021 | 0.0022 | 0.0015 |
| 38 | 0.0012 | 0.0007 | 0.0011 | 0.0006 | 88 | 0.0030 | 0.0021 | 0.0022 | 0.0015 |
| 39 | 0.0013 | 0.0008 | 0.0012 | 0.0006 | 89 | 0.0030 | 0.0021 | 0.0022 | 0.0015 |
| 40 | 0.0015 | 0.0008 | 0.0012 | 0.0006 | 90 | 0.0030 | 0.0021 | 0.0022 | 0.0015 |
| 41 | 0.0016 | 0.0009 | 0.0013 | 0.0007 | 91 | 0.0030 | 0.0021 | 0.0022 | 0.0015 |
| 42 | 0.0016 | 0.0010 | 0.0013 | 0.0008 | 92 | 0.0030 | 0.0021 | 0.0022 | 0.0015 |
| 43 | 0.0018 | 0.0011 | 0.0015 | 0.0008 | 93 | 0.0030 | 0.0021 | 0.0022 | 0.0015 |
| 44 | 0.0019 | 0.0012 | 0.0016 | 0.0009 | 94 | 0.0030 | 0.0021 | 0.0022 | 0.0015 |
| 45 | 0.0022 | 0.0013 | 0.0017 | 0.0010 | 95 | 0.0030 | 0.0021 | 0.0022 | 0.0015 |
| 46 | 0.0022 | 0.0014 | 0.0018 | 0.0010 | 96 | 0.0030 | 0.0021 | 0.0022 | 0.0015 |
| 47 | 0.0024 | 0.0016 | 0.0019 | 0.0011 | 97 | 0.0030 | 0.0021 | 0.0022 | 0.0015 |
| 48 | 0.0025 | 0.0017 | 0.0020 | 0.0012 | 98 | 0.0030 | 0.0021 | 0.0022 | 0.0015 |
| 49 | 0.0028 | 0.0018 | 0.0023 | 0.0013 | 99 | 0.0030 | 0.0021 | 0.0022 | 0.0015 |
| 50 | 0.0030 | 0.0021 | 0.0022 | 0.0015 | 100 | 0.0030 | 0.0021 | 0.0022 | 0.0015 |

The rate of other cause mortality by age and sex was treated as the baseline hazard. Following input from stakeholders, an increased risk of mortality was assigned to individuals with diabetes using data from a published meta-analysis (50). This study used data from 820,900 people from 97 prospective studies to calculate hazard ratios for cause-specific death, according to baseline diabetes status (50). Cause of death was separated into vascular disease, cancer and other cause mortality. From this study we estimated that individuals with a diagnosis of diabetes have a fixed increased risk of other cause mortality (Hazard ratio 1.8 (95% CI 1.71-1.9)). The estimates reported in the meta-analysis include increased risk of death from renal disease, therefore mortality from renal disease was not simulated separately to avoid double counting of benefits.

Mortality risk increases with the onset of dementia. As a consequence, all cause mortality was inflated after diagnosis of dementia. The hazard ratio of mortality was estimated from analysis of two United States cohorts (51). Participants were recruited to the studies without known dementia at baseline and received annual clinical evaluation and brain donation at death. The analysis included 2566 persons over 8 years and found a hazard ratio of death with all dementia of 4.54 (CI 3.54-5.83) for ages 75-84 and 2.77 (CI 2.37-3.23) for ages 85 and older . These hazard ratios were applied in the model to all cause mortality to describe mortality at younger ages 60-84 and older 85+ ages.

Mortality rates for individuals with diabetes and undiagnosed diabetes are also at increased risk of mortality, which is applied to all cause mortality. Given the correlation between risk factors of diabetes and dementia, and the high prevalence of multi-comorbidities in later life, it is necessary to adjust mortality risk for individuals with both dementia and diabetes. We believe that applying both mortality hazard ratios would over-estimate the mortality burden in these patients. In the model the higher hazard ratio for Dementia is applied.

# Direct Health Care Costs

At any given time period of the model individuals can have multiple health complications that incur direct healthcare costs. Some of the health states are mutually exclusive; however an individual can accrue multiple complications within the model. Each health state is associated with an average cost, which is accrued by all individuals for every time period for which the state is indicated. Resource use for each comorbidity is added together and no savings are assumed to be made from the use of the same resources for two or more comorbidities for an individual.

In some instances we have adopted costs and prices from old studies. We have inflated all prices and costs to 2020/21 prices using inflation indices reported in the Personal Social Services Research Unit (PSSRU) (52). This documents health related inflation up to 2020/21 prices. The retail price index was used to inflate costs to 2020/21 prices.

Primary care and community care costs were sought from the Personal Social Services Research Unit (PSSRU) (52), and secondary care costs from UK reference costs (53). Drug costs were obtained from the British National Formulary (54). In most instances costs for long term health outcomes were sought from recent Health Technology Appraisals as this was thought to be the best source of evidence for costs and resource use by disease area in the UK. If an HTA appraisal was not identified, searches for good quality cost-effectiveness analyses for the relevant disease area were conducted to identify the appropriate UK costs.

## GP attendance

The costs of each visit to a General Practitioner were estimated at £39 from the Personal Social Services Research Unit (PSSRU), assuming 9.22 minute appointments (52).

Diabetes diagnosis incurred a cost of £16 (2020 prices) in line with costs used for a previous evaluation of a Diabetes Prevention Programme (3).

Recent guidelines for hypertension have recommended that hypertension be confirmed with ambulatory blood pressure monitoring (ABPM) (18). The cost of ABPM assessment is included in the cost of diagnosis (£45.34) (55), however, we assume that the test does not alter the initial diagnosis.

The cost of identifying individuals to receive statins is assumed to be negligible because cases are detected using existing cardiovascular risk programmes used by the GP.

## Diabetes

We were advised by stakeholders to model a simplified diabetes treatment pathway. It was recommended that a single annual cost of prescriptions be applied to all patients diagnosed with diabetes. Initially we explored this as an option but concluded that the timing of more costly treatments for type 2 diabetes is important because treatment costs will be discounted. The model assesses interventions that lower HbA1c and so have the potential to impact on the level of treatment required.

We decided to implement a three stage treatment regimen as a trade-off between model simplicity and capturing key cost differences between the interventions. At diagnosis all patients are prescribed low cost treatments, such as Metformin and Sulfonylurea. We chose Metformin, 500mg/day to describe the average cost of these medications. If HbA1c increases above a threshold the individual is prescribed the more expensive Gliptins in addition to Metformin. The individual continues to receive Metformin plus Gliptins for a period of time until they require insulin.

### Metformin Monotherapy

Cost estimates from the British National Formulary indicate that the cost of Metformin is approximately £19 per annum, using a combination of standard and modified release (54).

Other resource use costs and resource utilisation assumptions for diabetics receiving Metformin monotherapy are detailed in Table 33. Drug costs was assumed to not vary, while the other costs were assumed to have a standard error equal to 1/10^th^ their mean.

Table S33: Drug costs and resource utilisation costs for low cost diabetes monotherapy

| **Resource** | **Assumption for costs** | **Source** | **Unit Cost 2020 prices** | **Annual utilisation** | **SE** | **Distribution** | **Cost per year** |
| --- | --- | --- | --- | --- | --- | --- | --- |
| Metformin | 500mg *bid* standard (85% of patients) or modified release (15%) tablets | (54) | 0.026 | 730 |  | CONSTANT | 18.98 |
| Nurse at GP | Advanced GP Nurse per hour for 15 minute appointment | (52) | 44.00 | 0.25 | 4.4 | GAMMA | 11.00 |
| GP Nurse | Band 4 – Qualified Nurse at GP practice per hour for 10 minute appointment | (52) | 33.00 | 0.167 | 3.3 | GAMMA | 5.50 |
| Urine sample | Clinical Biochemistry | (53) | 2.00 | 1 | 0.2 | GAMMA | 2.00 |
| Eye screening | Non-Consultant Lead Ophthalmology | (53) | 140.80 | 1 |  | CONSTANT | 140.80 |
| HbA1c | Haematology | (53) | 4 | 1 | 0.4 | GAMMA | 4 |
| Lipids | Clinical Biochemistry | (53) | 2.00 | 1 | 0.2 | GAMMA | 2.00 |
| Liver function | Clinical Biochemistry | (53) | 2.00 | 1 | 0.2 | GAMMA | 2.00 |
| B12 | Clinical Biochemistry | (53) | 2.00 | 1 | 0.2 | GAMMA | 2.00 |
|  | | | Total annual cost | | | | 188.28 |

The cost of diabetes in the year after diagnosis is assumed to be greater than subsequent years because the individual will receive more contact time whilst their diabetes is being controlled. The additional costs of diabetes in the year after diagnosis are reported in Table S34.

Table S34: Drug costs and resource utilisation costs for the first year after diabetes diagnosis

| **Resource** | **Assumption for costs** | **Source** | **Unit Cost 2020 prices** | **SE** | **Distribution** | **Annual utilisation** | **Cost per year** |
| --- | --- | --- | --- | --- | --- | --- | --- |
| Nurse at GP | Advanced GP Nurse per hour for 15 minute appointment | (52) | 44.00 | 4.4 | GAMMA | 0.5 | 22.00 |
| GP Nurse | Band 4 – Qualified Nurse at GP practice per hour for 10 minute appointment | (52) | 33.00 | 3.3 | GAMMA | 0.33 | 11.00 |
| Urine sample | Clinical Biochemistry | (53) | 2.00 | 0.2 | GAMMA | 2 | 4.00 |
| HbA1c | Haematology | (53) | 4 | 0.4 | GAMMA | 2 | 8.00 |
| Lipids | Clinical Biochemistry | (53) | 2.00 | 0.2 | GAMMA | 2 | 4.00 |
| Liver function | Clinical Biochemistry | (53) | 2.00 | 0.2 | GAMMA | 2 | 4.00 |
| B12 | Clinical Biochemistry | (53) | 2.00 | 0.2 | GAMMA | 2 | 4.00 |
| Smoking Cessation | Nicotine replacement therapy (Assumed 20% smoking prevalence and 50% uptake of smoking cessation services) | (52) | 128.00 | 12.80 | GAMMA | 0.1 | 12.80 |
|  | | | | | Total annual cost | | 69.80 |

### Metformin plus Gliptins

Simulated individuals experience an annual increase in HbA1c. Gillett et al. (2012) assume that individuals switch to dual treatment if HbA1c increases above 7.4% (56). Within the model, the individual is switched to a dual treatment in the first annual cycle in which HbA1c exceeds 7.4%. For costing purposes the second drug to be added to Metformin was Sitagliptin, which is reported in the British National Formulary to cost £1.19 per day (54). Belsey et al. (2009) report that 48% of patients used monitoring strips at a mean weekly consumption of 3.3 (57). Table S35 reports the other resource use costs and utilisation assumptions for diabetics receiving Metformin plus Gliptins.

Table S35: Drug costs and resource utilisation costs for Metformin and Gliptins

| **Resource** | **Assumption for costs** | **Source** | **Unit Cost 2020 prices** | **SE** | **Distribution** | **Annual utilisation** | **Cost per year** |
| --- | --- | --- | --- | --- | --- | --- | --- |
| Sitagliptin | 100mg per day from packet of 28-tabs of 100mg | (54) | 1.19 |  | CONSTANT | 365 | 434.35 |
| Metformin | 500mg *bid* standard (85% of patients) or modified release (15%) tablets | (54) | 0.026 |  | CONSTANT | 730 | 18.98 |
| Self-monitoring strips | 50 strip pack from National Diagnostic Products, 11p per strip | (54) | 0.11 |  | CONSTANT | 82.2 | 9.04 |
| Nurse at GP | Advanced GP Nurse per hour for 15 minute appointment | (52) | 44.00 | 4.4 | GAMMA | 0.25 | 11.00 |
| GP Nurse | Band 4 – Qualified Nurse at GP practice per hour for 10 minute appointment | (52) | 33.00 | 3.3 | GAMMA | 0.167 | 5.50 |
| Urine sample | Clinical Biochemistry | (53) | 2.00 | 0.2 | GAMMA | 1 | 2.00 |
| Eye screening | Non-Consultant Lead Ophthalmology | (53) | 140.80 |  | CONSTANT | 1 | 140.80 |
| HbA1c | Haematology | (53) | 4.00 | 0.4 | GAMMA | 1 | 4.00 |
| Lipids | Clinical Biochemistry | (53) | 2.00 | 0.2 | GAMMA | 1 | 2.00 |
| Liver function | Clinical Biochemistry | (53) | 2.00 | 0.2 | GAMMA | 1 | 2.00 |
| B12 | Clinical Biochemistry | (53) | 2.00 | 0.2 | GAMMA | 1 | 2.00 |
|  | | | | | Total annual cost | | £631.67 |

### Insulin plus Oral Anti-diabetics

The second major treatment change is assumed to be initiation of insulin. Gillett et al. (2012) assumed that individuals switch to insulin if HbA1c increases above 8.5% (56). Within the model the individual is switched to insulin in the first annual cycle at which HbA1c exceeds 8.5%. The insulin Glargine was chosen to represent insulin treatment in the UK and is consistent with Gillett et al. (2012) (56). The total resource use and costs of this health state are reported in Table S36 & Table S37.

Table S36: Costs of insulin treatment

|  | **Cost before Inflation (2006 prices)** | **Inflation** | **Unit cost 2020 prices** | **Source** |
| --- | --- | --- | --- | --- |
| Insulin Glargine | £628.44 |  |  | (58) |
| Oral anti-diabetics | £43.68 |  |  |  |
| Reagent test strips | £221.43 |  |  |  |
| Hypoglycaemic rescue | £23.43 |  |  |  |
| Pen delivery devices | £54.79 |  |  |  |
| Sharps | £68.82 |  |  |  |
| Total cost per year | £1,013.51 | 1.31 | 1328.02 |  |

Table S37: Drug costs and resource utilisation costs for insulin and oral anti-diabetics

| **Resource** | **Assumption for costs** | **Source** | **Unit Cost 2020 prices** | **SE** | **Distribution** | **Annual utilisation** | **Cost per year** |
| --- | --- | --- | --- | --- | --- | --- | --- |
| Insulin Treatment Costs | Total Annual Cost | See Table S37 | 1328.02 | 743.09 | GAMMA | 1 | 1328.02 |
| Nurse at GP | Advanced GP Nurse per hour for three 15 minute appointment | (52) | 44.00 | 4.4 | GAMMA | 0.75 | 33.00 |
| GP Nurse | Band 4 – Qualified Nurse at GP practice per hour for three 10 minute appointments | (52) | 33.00 | 3.3 | GAMMA | 0.5 | 16.50 |
| Urine sample | Clinical Biochemistry | (53) | 2.00 | 0.2 | GAMMA | 3 | 6.00 |
| Eye screening | Non-Consultant Lead Ophthalmology | (53) | 140.80 |  | CONSTANT | 1 | 140.80 |
| HbA1c | Haematology | (53) | 4.00 | 0.4 | GAMMA | 3 | 12.00 |
| Lipids | Clinical Biochemistry | (53) | 2.00 | 0.2 | GAMMA | 3 | 6.00 |
| Liver function | Clinical Biochemistry | (53) | 2.00 | 0.2 | GAMMA | 3 | 6.00 |
| B12 | Clinical Biochemistry | (53) | 2.00 | 0.2 | GAMMA | 3 | 6.00 |
|  | | | | | Total annual cost | | 1554.32 |

## Statins

We assumed that individuals who are prescribed statins receive a daily dose of 40mg of generic Simvastatin. The British National Formulary reports a cost of approximately 3p per 20mg tablet, generating an average annual cost of statins of £19.71 (54). The individual remains on statins for the rest of their life. The cost of GP attendance was not included in the cost of statins to avoid double counting of GP attendance.

## Anti-hypertensives

The estimated annual costs of hypertension were taken from the NICE guidelines for hypertension in adults (NG136) (59). The guideline reports the annual cost of medication, which we assume to be maintained in subsequent years of having hypertension, and annual monitorying costs for both the first year and subsequent years. The costs associated with hypertension are reported in Table S38 and Table S39.

Table S38: Annual cost of anti-hypertensive treatment expenditure per patient in first year (£)

| **Resource** | **Assumption for costs** | **Source** | **Unit Cost before inflation** | **Unit cost 2020 prices** | **SE** | **Distribution** | **Annual utilisation** | **Cost per year** |
| --- | --- | --- | --- | --- | --- | --- | --- | --- |
| Drug Costs | Weight averages of BNF drug costs based on distribution of number and class of drugs. | (59) | 18.02 | 18.58 | 1.86 | GAMMA | 1 | 18.575 |
| Monitoring Costs | Monitoring costs during first year of treatment | (59) | 121.5 | 125.24 | 12.52 | GAMMA | 1 | 125.242 |
|  | | | | | | Total annual cost | | 143.82 |

Table S39: Annual cost of anti-hypertensive treatment expenditure per patient in subsequent years (£)

| **Resource** | **Assumption for costs** | **Source** | **Unit Cost 2019 prices** | **Unit cost 2020 prices** | **SE** | **Distribution** | **Annual utilisation** | **Cost per year** |
| --- | --- | --- | --- | --- | --- | --- | --- | --- |
| Drug Costs | Weight averages of BNF drug costs based on distribution of number and class of drugs. | (59) | 18.02 | 18.58 | 1.86 | GAMMA | 1 | 18.575 |
| Monitoring Costs | Monitoring costs during subsequent years of treatment | (59) | 75 | 77.31 | 7.73 | GAMMA | 1 | 77.310 |
|  | | | | | | Total annual cost | | 95.89 |

## Cardiovascular Events

Costs for coronary heart disease disease were obtained from a 2009 HTA for high dose lipid-lowering therapy unless otherwise stated (17). The costs of stroke were obtained from a study estimating costs from the Oxford vascular cohort (60). Table S40 describes the costs and resource use assumptions that were used for this study. It also reports the health states to which we have applied each cost in the model. The costs of congestive heart failure were estimated from the UKPDS costing study for complications related to diabetes (61). The unit costs for cardiovascular events are detailed in Table S40.

Table S40: Unit costs for Cardiovascular cost estimates

| **Event** | **Time** | **Assumption** | **Mean cost** | **Source** | **Mean cost 2020 prices** | **SE** | **Distribution** |
| --- | --- | --- | --- | --- | --- | --- | --- |
| Stable Angina |  |  | £533.40 | See Table S41 | £533.40 |  |  |
| Unstable Angina | Event year | First 6 month cost plus 6 * monthly average cost | £2244.93 | (62) | 2507.48 | 250.75 | GAMMA |
|  | Subsequent Years | 12* Monthly average cost | £131.38 | (62) | 146.74 | 14.67 | GAMMA |
| Transient Ischemic Attack | Event year | First 6 month cost plus 6 * monthly average cost | £1677.83 | (62) | 1874.06 | 187.41 | GAMMA |
|  | Subsequent Years | 12* Monthly average cost | £281.90 | (62) | 314.87 | 31.49 | GAMMA |
| Stroke | Event year | First year cost | £13459.00 | (63) | 14980.61 | 1498.06 | GAMMA |
|  | Subsequent Years | Average of remaining 4 years of costs | £1118.00 | (63) | 1244.40 | 1995.82 | GAMMA |
| MI | Event year | First 6 month cost plus 6 * monthly average cost | £4459.90 | (62) | 4981.48 | 498.15 | GAMMA |
|  | Subsequent Years | 12* Monthly average cost | £368.97 | (62) | 412.12 | 41.21 | GAMMA |
| Congestive Heart Failure | Event year | Inpatient + non-inpatient minus subsequent year costs |  | (64) | 1964.33 |  | GAMMA |
|  | Subsequent Years | Inpatient + non-inpatient |  | (64) | 2786.97 |  | GAMMA |
| Fatal CHD | Event year |  | £2071 | (65) | £2399.81 | 239.98 | GAMMA |
| Fatal stroke | Event year |  | £1737 | (65) | £2012.78 | 201.28 | GAMMA |

Table S41: Unit costs for Stable Angina

| **Resource** | **Assumption** | **Mean Cost** | **Source** | **Mean (2020 prices)** | **SE** | **Distribution** | **Utilisation** | **Annual Cost** |
| --- | --- | --- | --- | --- | --- | --- | --- | --- |
| Initial Appointment | 3 GP visits | £117 | (52) (17) | £127.52 |  | CONSTANT |  |  |
| Subsequent Appointment | Weighted average of all CC scores of cardiac non-consultant outpatients surgery | £207.1 | (66) | £226.12 | 22.61 | GAMMA |  |  |
| Glytrin Spray | 90% of patients receive glytrin spray, isobide monoitrate, one of verapamil or atenolol, and aspirin | 10.47 | (17) | £12.84 |  | CONSTANT | 0.9 | £71.13 |
| Isosorbide mononitrate |  | 11.24 | (17) | £13.78 |  | CONSTANT |  |  |
| Verapamil |  | 41.98 | (17) | £51.46 | 5.15 | GAMMA |  |  |
| Atenolol |  | 30.24 | (17) | £37.07 | 3.71 | GAMMA |  |  |
| Aspirin |  | 6.65 | (17) | £8.15 | 0.82 | GAMMA |  |  |
| Ramipril | 90% of patients receive rampiril | 75.09 | (17) | £92.05 | 9.21 | GAMMA | 0.9 | £82.84 |
| ARB | 10% of patients receive ARB | 210.27 | (17) | £257.77 | 25.78 | GAMMA | 0.1 | £25.78 |
| **Stable Angina** | **Total cost is assumed to be a combination of initial and subsequent appointments plus average medication use.** |  |  |  |  |  |  | **£533.40** |

## Renal Failure

The cost of renal failure taken from NICE Health economic model report on type 2 diabetes (67). for the UK using relevant published studies. The reported healthcare costs in the first year of occurring the event are £20,897 and the costs or subsequent years after the event are £8,332 at 2020 prices. Both costs are assumed to have a standard error equal to 1/10^th^ of the mean and sampled using a gamma distribution.

## Foot Ulcers

The annual cost of foot ulcers was taken from a study using Hospital Episodic Statistics data for England to estimate healthcare costs of diabetic foot disease (68). The costs were inflated to 2020 prices and an annual NHS cost of £3,932 was applied in the model. This cost was sampled, assuming a standard error of 1/10^th^ the mean, using a gamma distribution.

## Amputation

The cost of amputation in the first year of surgery and subsequent years has been reported in a UKPDS costing study (64). The costs were extracted and inflated to 2020 prices. The cost of amputation in the first year was £13,952 and in subsequent years was £3,877. The unit costs for amputation are reported in Table S43.

Table S42: Unit costs for amputation

| **Unit Cost** | **Assumption** | **Mean** | **Source** | **Mean (2020 prices)** | **SE** | **Distribution** | **Annual Cost** |
| --- | --- | --- | --- | --- | --- | --- | --- |
| Inpatient | Event Year | 9546 | (64) | 10876.71 | 1819.55 | GAMMA | £13,952 |
| Non-inpatient | Event Year | 2699 | (64) | 3075.24 | 749.91 | GAMMA |  |
| Inpatient | Subsequent Years | 1792 | (64) | 2041.81 | 425.53 | GAMMA | £3,877 |
| Non-inpatient | Subsequent Years | 1611 | (64) | 1835.57 | 242.99 | GAMMA |  |

## Blindness

The cost of blindness in the first year of surgery and subsequent years has been reported In a UKPDS costing study (64). The costs were extracted and inflated to 2020 prices. The cost of blindness in the first year was £3,583 and in subsequent years was £1,357, the sum of inpatient and non-inpatient costs. The unit costs for blindeness are reported in Table S44.

Table S43: Unit costs of Blindness

| **Resource** | **Assumption** | **Mean** | **Source** | **Mean (2020 prices)** | **SE** | **Distribution** | **Annual Cost** |
| --- | --- | --- | --- | --- | --- | --- | --- |
| Inpatient | Event Year | 1355 | (64) | 1543.89 | 546.45 | GAMMA | £3,583 |
| Non-inpatient | Event Year | 1790 | (64) | 2039.53 | 530.17 | GAMMA |  |
| Inpatient | Subsequent Years | 453 | (64) | 516.15 | 80.22 | GAMMA | £1,357 |
| Non-inpatient | Subsequent Years | 738 | (64) | 840.88 | 94.17 | GAMMA |  |

## Cancer

The cost of breast and colorectal cancer is estimated as a one-off fixed cost at diagnosis in the model. This simplifying assumption means that the cost of cancer treatment is independent of survival. We acknowlegde that this assumption will affect the timing of costs because all costs are imposed in the first year and subject to less discounting. However, we anticipate that the impact on overall outcomes will not be substantial. A large proportion of costs are will be incurred in the first year of treatment (surgery, chemotherapy, radiotherapy). Costs in subsequent years will be lower for patients who achieve remission and survival will be short in patients who relapse. Therefore, the costs are likely to be skewed to the early years post diagnosis.

The cost of breast and colorectal cancer were taken from a retrospective cohort study which estimated the total cost of care for breast and colorectal cancer for 9 years post diagnosis (69). The study reported cost by age category and by stage of cancer. These were weighted by the proportion of patients facing each reported in the paper. **Table S45** reports the overall cost of breast cancer by stage of disease at diagnosis and age. Table S46 reports the overall cost of colorectal cancer by stage of disease at diagnosis and age. Samples of total cost were taken using an assumed standard error of 1/10^th^ of the mean cost, using a gamma distribution.

**Table S44: Estimated cost of breast cancer**

| **Resource component** | **Annual price** | **Price Year** | **Inflation** | **Proportion** | **Weighted Average Cost** |
| --- | --- | --- | --- | --- | --- |
| Total cost (9 years after diagnosis) for stages 1-2 for ages 18-64 | £22,502 | 2010 | 1.183 | 0.49 | £11,054.65 |
| Total cost (9 years after diagnosis) for stages 3-4 for ages 18-64 | £35,244 | 2010 | 1.183 | 0.07 | £2,316.45 |
| Total cost (9 years after diagnosis) for stages 1-2 for ages 65+ | £19,479 | 2010 | 1.183 | 0.35 | £7,205.38 |
| Total cost (9 years after diagnosis) for stages 3-4 for ages 65+ | £25,698 | 2010 | 1.183 | 0.07 | £1,878.40 |
| Propotion stages 1-2 for ages 18-64 | 0.882 | | | |  |
| Propotion stages 1-2 for ages 65+ | 0.835 | | | |  |
| Proportion aged 65+ | 0.443 | | | |  |
|  | | Total cost (2010 prices) | | | £22,454.87 |
|  | | Total Inflated Cost (2020 prices) | | | £26,566.45 |

Table S45: Estimated cost of colorectal cancer

| **Resource component** | **Total Cost** | **Price Year** | **Inflation** | **Proportion** | **Weighted Average Cost** |
| --- | --- | --- | --- | --- | --- |
| Total cost (9 years after diagnosis) for stages 1-2 for ages 18-64 | £12,938 | 2010 | 1.183 | 0.12 | £1,582.14 |
| Total cost (9 years after diagnosis) for stages 3-4 for ages 18-64 | £21,128 | 2010 | 1.183 | 0.15 | £3,057.52 |
| Total cost (9 years after diagnosis) for stages 1-2 for ages 65+ | £13,843 | 2010 | 1.183 | 0.38 | £5,276.40 |
| Total cost (9 years after diagnosis) for stages 3-4 for ages 65+ | £17,282 | 2010 | 1.183 | 0.35 | £6,080.50 |
| Propotion stages 1-2 for ages 18-64 | 0.46 | | | |  |
| Propotion stages 1-2 for ages 65+ | 0.52 | | | |  |
| Proportion aged 65+ | 0.73 | | | |  |
|  | Total cost (2010 prices) | | | | £15,996.55 |
|  | Total Inflated Cost (2020 prices) | | | | £18,925.58 |

## Osteoarthritis

The annual cost of osteoarthritis were estimated in a report in 2010 (70). In this report the authors estimated the expected cost of osteoarthritis from three previous costing studies. The costs include GP attendance, nurse consultations, replacement surgery, help at home and prescription medications. The estimated annual cost of osteoarthritis was £783 in 2008. In the study 93% of the costs were attributable to direct medical costs and 7% to social care. Therefore, cost of direct medical care in 2020 prices is £894.91. Samples are taken assuming a standard eror that is 1/10^th^ of the mean cost, using a gamma distribution.

## Depression

Depression is modelled as a chronically recurrent disorder, with patients experiencing further depressive episodes after remission. In the model it is assumed that patients continue to incur costs of depression following an initial diagnosis. These costs reflect ongoing resource use to deal with relapse and prevention of relapse.

A recent trial to prevent secondary depressive episodes collected comprehensive cost data from a sample of individuals with depression (71). The resource uses identified in the control arm were extracted to estimate the costs of depression, with staff costs updated using NHS unit cost collection (52). The costs from this data (inflated to 2020 prices) were not implemented directly into the SPHR diabetes prevention model as this would have over-estimated the number of GP visits. The model already accounts for GP attendance due to depression. Therefore, a revised estimate of the cost of depression, excluding GP consultation was estimated using updated unit costs. All costs used were sampled assumoing a standard error that is 1/10^th^ of the mean, using a gamma distribution. The resource use estimates and revised unit cost estimates used to generate a cost of depression excluding GP utilisation are reported in Table S47.

Table S46: Depression utilisation of services and total estimated cost

|  | Assumption for costs | Unit cost | Source of cost | Price year | Inflation | Unit cost 2020 prices | | Annual utilisation | Source of utilisation | Cost per year |
| --- | --- | --- | --- | --- | --- | --- | --- | --- | --- | --- |
| Practice nurse at surgery | GP nurse face to face assume 10 mins | £7.33 | (52) | 2020 | 1 | £7.33 | | 1.52 | (71) | £11.14 |
| Practice nurse at home visit | GP nurse face to face assume 30 mins | £22.00 | (52) | 2020 | 1 | £22.00 | | 0.02 | (71) | £0.44 |
| Practice nurse telephone | GP nurse face to face assume 10 mins | £7.33 | (52) | 2020 | 1 | £7.33 | | 0.11 | (71) | £0.81 |
| Health visitor | GP nurse face to face assume 30 mins | £22.00 | (52) | 2020 | 1 | £22.00 | | 0.05 | (71) | £1.10 |
| District nurse | GP nurse face to face assume 30 mins | £22.00 | (52) | 2020 | 1 | £22.00 | | 0.01 | (71) | £0.22 |
| Other nurse | GP nurse face to face assume 10 mins | £7.33 | (52) | 2020 | 1 | £7.33 | | 0.13 | (71) | £0.95 |
| HCA phlebotomist | Clinical support worker 10 mins | £2.17 | (71) | 2009 | 1.219 | £2.64 | | 0.31 | (71) | £0.82 |
| Other primary care | Advanced nurse with qualifications | £14.48 | (71) | 2009 | 1.219 | £17.65 | | 0.19 | (71) | £3.35 |
| Out of hours | Inflated of trial costs | £5.13 | (71) | 2009 | 1.2045 | £6.25 | | 0.23 | (71) | £1.44 |
| NHS direct | Inflated of trial costs | £1.89 | (71) | 2009 | 1.2045 | £2.30 | | 0.09 | (71) | £0.21 |
| Walk-in centre | Inflated of trial costs | £6.77 | (71) | 2009 | 1.2045 | £8.25 | | 0.21 | (71) | £1.73 |
| Prescribed medications | Inflated of trial costs | £61.87 | (71) | 2009 | 1.2045 | £75.40 | | 7.74 | (71) | £583.55 |
| Secondary care | Emergency Medicine, Any Investigation | £23.85 | (71) | 2009 | 1 | £29.06 | | 0.26 | (71) | £7.56 |
|  | | | | | | | Total Annual Cost | | | £613.32 |

## Dementia

### Cost of Diagnosis

A one off cost of diagnosis is incurred in the first year of the disease to account for the costs associated with assessing and diagnosing patients. The most recent cost study of Dementia for the UK estimated the cost of Dementia diagnosis at £650 in 2012/13 prices (47) inflated to £740.61.

### Ongoing healthcare costs

The direct health care costs of dementia to the NHS were estimated in an Alzheimers UK report in 2014. The costs were estimated from a modelling study based on PSSRU aggregeate long term care model and PSSRU dementia care model. The report describes costs of care for patients with dementia in 2013 £. Full details of the costing model are reported elsewhere. Samples were taken assuming a standard error that is 1/10^th^ of the mean, using a gamma distribution. Table S48 reports the costs of dementia for individuals in community or residential care according to MMSE cognitive score. In the model it is assumed that healthcare costs are met entirely by the NHS. These costs are applied in the model to patients with a dementia diagnosis on an annual basis.

Table S47: Average annual direct healthcare dementia costs

|  | Healthcare costs | | Proportion of patients residential care | Total cost 2012 prices | Total Costs 2020 prices |
| --- | --- | --- | --- | --- | --- |
|  | Community | Residential |  |  |  |
| Mild (MMSE 21-26) | 2,751 | 4,504 | 10.4% | 2932 | £3340.72 |
| Moderate (MMSE 10-20) | 2,695 | 9,438 | 76.2% | 7837 | £8929.48 |
| Severe (MMSE 0-9) | 11,258 | 8,689 | 76.2% | 9300 | £10596.42 |

# Social Care costs

In this analysis the social care costs refer to the public and private costs incurred with social care as a consequence of a diagnosis with osteoarthritis, stroke or dementia. Social care costs associated with the other health outcomes of the model are not included in this estimate. This is likely to under-estimate the overall cost of social care in the population. However, reliable social care costs for other conditions are very hard to obtain because they are less commonly incurred in the prevalent patient population and more likely to be attributed to other factors or ageing more generally.

**Osteoarthritis**

The annual cost of osteoarthritis were estimated in a report in 2010 (70). The estimated annual cost of osteoarthritis was £783 in 2008. In the study 93% of the costs were attributable to direct medical costs and 7% to social care. Therefore, cost of social care costs in 2020 prices at £65.

## Stroke

The community costs in the first year following stroke were estimated in a cost-effectivness analtsis using the Sentinel Stroke National Audit Program (63). They report the social care cost at 1 year post-event, £8,716, and the total cost 5 years post-event, £27,301. These, at 2015 prices, were inflated to 2020 prices. Individuals as assumed to face a social care cost of £9701.39 in the event year, and then a subsequent yearly cost of £5171.53 which was calculated by dividing the remaining total 5 year cost, after subtracting the first years costs, by 4. Samples of these costs were taken assuming a standard error of 1/10^th^ of the mean, using a gamma distribution. The unit costs for the social care associated with a stroke are reported in Table S49.

Table S48: Unit costs of social care for Stroke events

|  | Assumption | Annual Cost | Source | Price year | Inflation | Final annual cost |
| --- | --- | --- | --- | --- | --- | --- |
| Event year |  | £8,716 | (63) | 2015 | 1.11 | £9701.39 |
| Subsequent Years | Removing event year costs from total 5 year cost and finding average yearly cost | $£4,646.25$ | (63) | 2015 | 1.11 | £5171.53 |

## Dementia

The social care costs of dementia were estimated in an Alzheimers UK report in 2014 (47). The costs were estimated from a modelling study based on PSSRU aggregeate long term care model and PSSRU dementia care model. The report describes costs of care for patients with dementia in 2012 prices. Table S50 reports the costs of dementia for individuals in community or residential care according to MMSE cognitive score. We used estimates from the Alzheimers UK report to estimate the pubic and private social care costs of dementia in line with the methods used in this report. We do not include the productivity costs of informal carers or other public costs in the model. These costs are applied in the model to patients with a dementia diagnosis on an annual basis.

Table S49: Average annual dementia costs

|  | Healthcare costs | | Proportion of patients residential care | Total cost 2012 prices | Total Costs 2020 prices |
| --- | --- | --- | --- | --- | --- |
|  | Community | Residential |  |  |  |
| Mild (MMSE 21-26) | 3,121 | 24,737 | 10.4% | £5362 | £6109.46 |
| Moderate (MMSE 10-20) | 7,772 | 25,715 | 76.2% | £21455 | £24445.83 |
| Severe (MMSE 0-9) | 10,321 | 25,874 | 76.2% | £22176 | £25267.34 |

# Utilities

## Baseline Utility

Baseline utilities for all individuals in the cohort were extracted from the HSE 2018. Utility was assumed to decline due to ageing independent of health status. In the simulation, utility declines by an absolute decrement of 0.004 per year. This estimate is based on previous HTA modelling in cardiovascular disease (12). Utility estimates are also adjusted for changes to BMI. In line with evidence from longitudinal follow-up of individuals seeking weight loss, we apply a utility adjustment to weight loss or weight gain through a relationship with BMI (72). Weight gain over time is associated with worsening utility at -0.0111 (s.e. 0.0018) per unit change in BMI, whereas weight loss is associated with equivalent improvement in utility.This change to utility is only applied to individuals in the model with BMI>25.

## Utility Decrements

The utility decrements for long term chronic conditions were applied to the age adjusted EQ-5D score. In consultation with stakeholders, we assumed that a diagnosis of diabetes was not associated with a reduction in EQ-5D independent of the utility decrements associated with complications, comorbidities or depression. Cardiovascular disease, renal failure, amputation, foot ulcers, blindness, cancer, osteoarthritis and depression were all assumed to result in utility decrements. The utility decrements are measured as a factor which is applied to the individual’s age adjusted baseline. If individuals have multiple chronic conditions the utility decrements are multiplied together to give the individual’s overall utility decrement from comorbidities and complications, in line with current NICE guidelines for combining comorbidities (73).

Due to the number of health states it was not practical to conduct a systematic review to identify utility decrements for all health states. A pragmatic approach was taken to search for health states within existing health technology assessments for the relevant disease area or by considering studies used in previous economic models for diabetes prevention. Discussions with experts in health economic modeling were also used to identify prominent sources of data for health state utilities.

Two sources of data were identified for diabetes related complications. A study from the UKPDS estimated the impact of changes in health states from a longitudinal cohort (74). They estimated the impact of myocardial infarction, ischaemic heart disease, stroke, heart failure, amputation and blindness on quality of life using seven rounds of EQ-5D questionnaires administered between 1997 and 2007. This data was used to estimate the utility decrement for amputation and congestive heart failure. The absolute decrement for amputation was converted into utility decrement factors that could be multiplied by the individuals’ current EQ-5D to estimate the relative effect of the complication. Blindness was included in the statistical model used for this analysis however the UKPDS analysis reported an increase in health state utility following a diagnosis with blindness. Discussions with the authors highlighted that this was due to treatment following formal classification with blindness and it was decided that this increase in health state utility should not be included in the cost-effectiveness model.

Utility decrements for renal failure and foot ulcers were not available from the UKPDS study described above. A study by Coffey et al. (2000) was used to estimate utility decrements for renal failure and foot ulcers (75). In this study, 2,048 subjects with type 1 and type 2 diabetes were recruited from specialty clinics. The Self-Administered Quality of Well Being index (QWB-SA) was used to calculate a health utility score.

A meta-analysis of utility values for diabetes and diabetes related complications estimated utility decrements for amputation, ulcer, end stage renal failure and blindness (76). The study pooled utility measures using different health state valuation measures in a meta-analysis. Pooling health state utility values is problematic because of the fact that different valuation methods and different preference-based measures (PBMs) can generate different values on exactly the same clinical health state (77).There were not sufficient studies in the meta-analysis to adjust for the effects of health state valuation measure on the result. This is a limitation of the analysis and we decided that it was preferable to use estimates from single studies.

Utility decrements for cardiovascular events were taken from an HTA assessing statins to reflect the utility decrements in all patients (7) rather than using the UKPDS, which is only representative of a diabetic population. The study conducted a literature review to identify appropriate utility multipliers for stable angina, unstable angina, myocardial infarction and stoke. We used these estimates in the model and assume that transient ischaemic attack is not associated with a utility decrement in line with this HTA.

We identified a systematic review of breast cancer utility studies following consultation with colleagues with experience in this area. The review highlighted a single burden of illness study with a broad utility decrement for cancer (78), rather than utilities by cancer type or disease status. This study was most compatible with the structure of the cost-effectiveness structure. Within this study 1823 cancer survivors and 5469 age-, sex-, and educational attainment-matched control subjects completed EQ-5D questionnaires to estimate utility with and without cancer.

The utility decrement for osteoarthritis was taken from a Health Technology Assessment that assessed the clinical effectiveness and cost-effectiveness of glucosamine sulphate/hydrochloride and chondroitin sulphate in modifying the progression of osteoarthritis of the knee (79).

A review of cost-effectiveness studies highlights the scarcity of studies of health-related quality of life in depression (80). The utility studies identified in the review described depression states by severity and did not adjust for comorbid conditions. Furthermore, the valuations were variable between studies suggesting poor consistency in the estimations. Therefore, it was difficult to apply these in the model. We decided to use a study which had used the EQ-5D in an RCT, for consistency with our utility measure (81). They report an average post treatment utility of 0.67, from which we estimated the utility decrement compared with the average utility reported in the HSE dataset. The decrement was then converted into a relative utility reduction.

The quality of life impact of dementia is estimated from a study by Jonsson and colleagues (82). These utility values were idenfied and used in the most recent NICE HTA for Alzheimer disease (46). A systematic review of health state utilities for alzheimers disease discusses differences in health related quality of life in different settings (83). It is often assumed that patients in institutional settings will be more disabled and have poorer quality of life. However, the studies that compared utility between settings did not identify a statistically significant difference. Therefore, we only related utility to MMSE.

Table S51 reports the multiplicative utility factors that are used in the model to describe health utility decrements from comorbid complications. The mean absolute decrement estimated in each study is reported alongside the baseline utility for each study. The utility factor was estimated by dividing the implied health utility with the comorbidity by the baseline utility.

Table S50: Utility decrement factors

|  | Mean Absolute decrement | St. error absolute decrement | Baseline Utility | Multiplicative Utility Factor | Source |
| --- | --- | --- | --- | --- | --- |
| BMI | -0.011 | 0.002 |  |  | Breeze (72) |
| Foot ulcer | -0.099 | 0.013 | 0.689 | 0.856 | Coffey (75) |
| Amputation | -0.172 | 0.045 | 0.807 | 0.787 | UKPDS (74) |
| Blind |  |  |  | 1.00 | Assumption |
| Renal failure | -0.078 | 0.026 | 0.689 | 0.887 | Coffey (75) |
| Stable Angina |  |  |  | 0.801 | Ward HTA (12) |
| Unstable Angina y1 |  |  |  | 0.770 | Ward HTA (12) |
| Unstable Angina y2 |  |  |  | 0.770 | Ward HTA (12) |
| Myocardial Infarction y1 |  |  |  | 0.760 | Ward HTA (12) |
| Myocardial Infarction y2 |  |  |  | 0.760 | Ward HTA (12) |
| Transient Ischaemic Attack |  |  |  | 1.000 | Ward HTA (12) |
| Stroke y1 |  |  |  | 0.629 | Ward HTA (12) |
| Stroke y2 |  |  |  | 0.629 | Ward HTA (12) |
| Breast Cancer | -0.060 | 0.008 | 0.791 | 0.913 | Yabroff (78) |
| Colorectal Cancer | -0.060 | 0.008 | 0.791 | 0.913 | Yabroff (78) |
| Osteoarthritis | -0.101 | 0.069 | 0.791 |  | Black HTA (79) |
| Depression | -0.116 |  | 0.791 | 0.875 | Benedict (81) |
| Congestive Heart Failure | -0.101 | 0.032 |  | 0.875 | UKPDS (74) |
| MMSE 26-30 |  |  | 0.690 |  | Jonsson (82) |
| MMSE 21-25 | -0.05 |  | 0.690 | 0.93 | Jonsson (82) |
| MMSE 15-20 | -0.19 |  | 0.690 | 0.725 | Jonsson (82) |
| MMSE 10-14 | -0.20 |  | 0.690 | 0.710 | Jonsson (82) |
| MMSE 0-9 | -0.36 |  | 0.690 | 0.478 | Jonsson (82) |
| UKPDS baseline utility 0.807; HSE baseline 0.7905 | | | | | |

# Intervention Effectiveness

## Intervention Effectiveness

### Elicitation Report

Four experts across 2 workshops were asked to estimate the difference in weight between the treatment arm receiving SWiM and the control arm receiving usual care at two different time points after beginning the program.

*Difference = SWiM weight (kg) – Usual Care weight (kg)*

If experts felt that those receiving SWiM would have a lower weight than those receiving usual care, the difference would be a negative value, and visa versa. As part of the elicitation exercise each expert reported values for lower plausible limit, upper plausible limit, median, and lower and upper quartiles. From these estimates we used SHELF software to fit a probability density function (PDF) to their values for the 12-month time point and 24-month time point conditional on the 12-month median difference. The workshops also sought to produce an aggregated PDF to represent the views and uncertainty of all experts through a deliberative process.

Workshop 1 took place first with 3 experts. Experts individually chose values for the upper and lower plausible limits, the quartiles, and the median. These were then collated and discussed as a group. Experts were tasked with combining all their thoughts and estimate one distribution that a rational and impartial observer would have chosen based on their individual views. Workshop 2 was carried out afterwards with a single expert. The participant chose values for the quantities of interest independently then compared their individual values to the outcome of workshop 1 and suggested adjustments to combine their estimates. The views of how the expert in workshop 2 felt about the distribution elicited from workshop 1 were noted. We report here a proposed PDF to reflect all views and aim to verify the combined distribution with all participants to ensure that it reflects their individual beliefs combined with the views of the other participants.

#### QOI 1: 12 months after the trial start date, what is the weight difference (kg) between the eligible population who receive the SWiM weight maintenance programme compared to the eligible population who receive the comparator?

##### Elicited values

There were some similarities between workshop views. Both workshops felt it was implausible that the treatment arm would have a higher weight than the control arm, thus all values elicited were negative to reflect lower weight for those receiving SWiM. Both workshops expressed a lower plausible limit of -4kgs. Workshop 2 felt that no difference in weight could be plausible but felt uncomfortable setting the upper limit at 0 as felt it was most likely that SWiM would improve weight outcomes. The medians differed slightly, with workshop 2 feeling on average the difference would be larger than that estimated by workshop 1. The differences in the interquartile range between experts were notable and workshop 1 opted for a wider interquartile range of to demonstrate more uncertainty in weight difference, while workshop 2 opted to set an interquartile range more centred around the median.

##### Distribution Fitting

There was variation in the best fitting distribution to the individually elicited quantities of interest. Both workshops set a negatively skewed distribution, with a larger probability that the weight difference will be closer to the upper plausible limit than the lower plausible limit. In workshop 1 the final agreed quantities and resulting distribution reflects a confidence amongst the group that the intervention would not result in fast weight regain. There was a lot of uncertainty about the true value it might take, with a belief that it could either reduce weight, or have negligible effect. The experts discussed that the intervention was just as likely to offer modest or strong benefits, which aligns well with the flat shaped beta distribution. In workshop 2 the adjusted distribution revised their reported upper plausible limit from -1 to 0, but the reported quantities produced a skewed distribution shape with a peak compared with the flatter beta distribution. Figure S4 illustrates the differences in the consensus distribution between workshop 1 and expert D’s individual distribution.


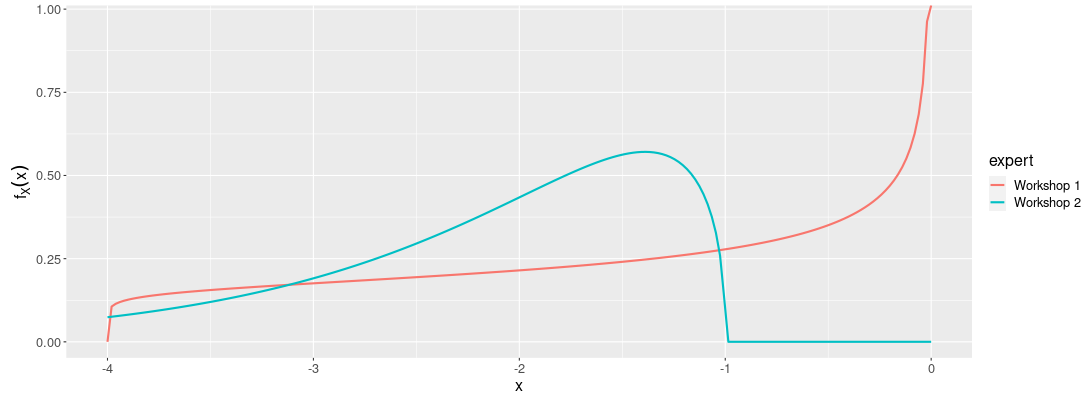

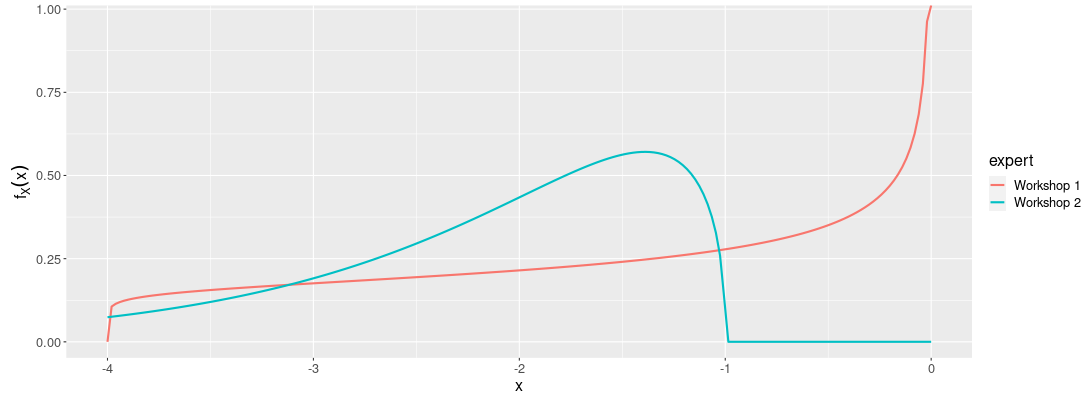


Figure S4: The distributions of 12-month difference in weight between treatment arms elicited in workshop 1 and workshop 2.

When discussing workshops 1 elicited distribution during workshop 2, expert D commented that the distribution was too flat and that a hump closer to the median would be more appropriate. To try and reflect this view on the distribution chosen in workshop 1, discussions with expert D resulted in a distribution that places the quartile 3 closer to the median at 0.8kgs.

##### Consensus Distribution

The consensus emerging from workshop 2 made the interquartile range narrower that that elicited from the first workshop (Table S52). The best fitting distribution to these values in a mirror log-normal distribution (Figure S5).

Table S51: A mathematical combination of both workshops elicited values.

| Workshop | L | 0.25 | 0.5 | 0.75 | U | Distribution |
| --- | --- | --- | --- | --- | --- | --- |
| Proposed consensus distribution | -4 | -2.5 | -1.3 | -0.8 | 0 | Mirror Log normal |


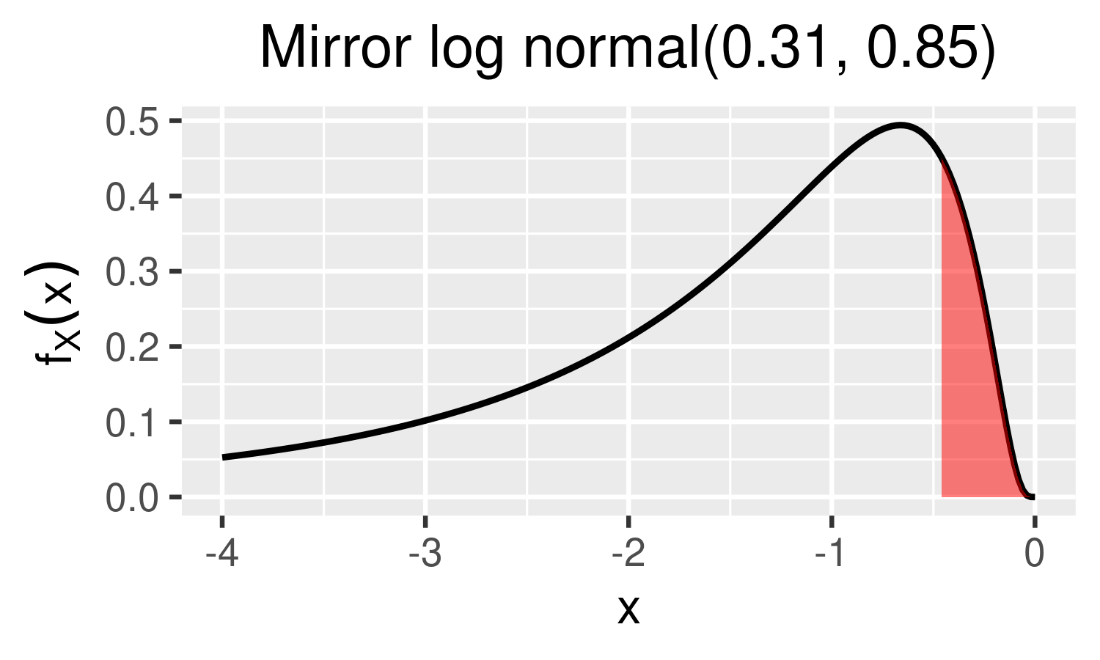


Figure S5: The consensus distributions of 12-month difference in weight between treatment arms

Sampling 10000 draws from the consensus distribution produces a median effect of -1.36, mean effect size of -1.96 and standard deviation 1.97.

#### QOI 2: What do you judge to be the likely values for the weight difference (kg) between SWiM and the comparator at 24 months?

##### Elicited values

Expectations of the effectiveness of SWiM at 24 months will depend on what happens at 12 months. For this quantity of interest we asked,

*“Given that SWiM changes weight by -1.3 (kg), what do you judge to be the likely values for the weight difference (kg) between SWiM and the comparator at 24 months?”*

The workshops here had very similar views. Both workshops felt was likely that if the intervention was effective at 12 months it was likely to maintain some benefits at 24 months. They also felt that, given a difference of -1.3kgs after 12 months, this difference would not increase to anymore than -3kgs. The medians were similar at -1.2kgs and -1kgs, however, as before, workshop 1 felt a wider interquartile range better represented their uncertainty, while workshop 2 was more confident setting a narrower IQ range around the median. Although, the difference here were small.

Both workshops felt they had to weigh up clinical experience with the evidence suggested of ACT and BT programs. Clinical experience often found weight to increase after a weight loss. However, the evidence of ACT has a more ‘positive’ outlook with either a longer maintenance of weight or further weight loss.

##### Distribution Fitting

There was variation in the best fitting distribution to the individually elicited quantities of interest. In workshop 1 the final resulting distribution reflects a confidence amongst the group that the intervention would not result in fast weight regain. There was a lot of uncertainty about the true value it might take, with a belief that it could either reduce weight, or have negligible effect. The facilitator fed back that the beta-distribution produces a flat shaped curve, which aligned with the discussion. Workshop 1 produced a flat distribution to reflect the uncertainty of difference between the upper and lower limit, while workshop 2 values fitted well with a more humped shape. In workshop 2 expert D revised their upper values in line with the views from workshop 1. They preferred a skewed distribution with lower probability of outcomes close to zero. Figure S6 shows a comparison of the consensus distribution from each workshop.


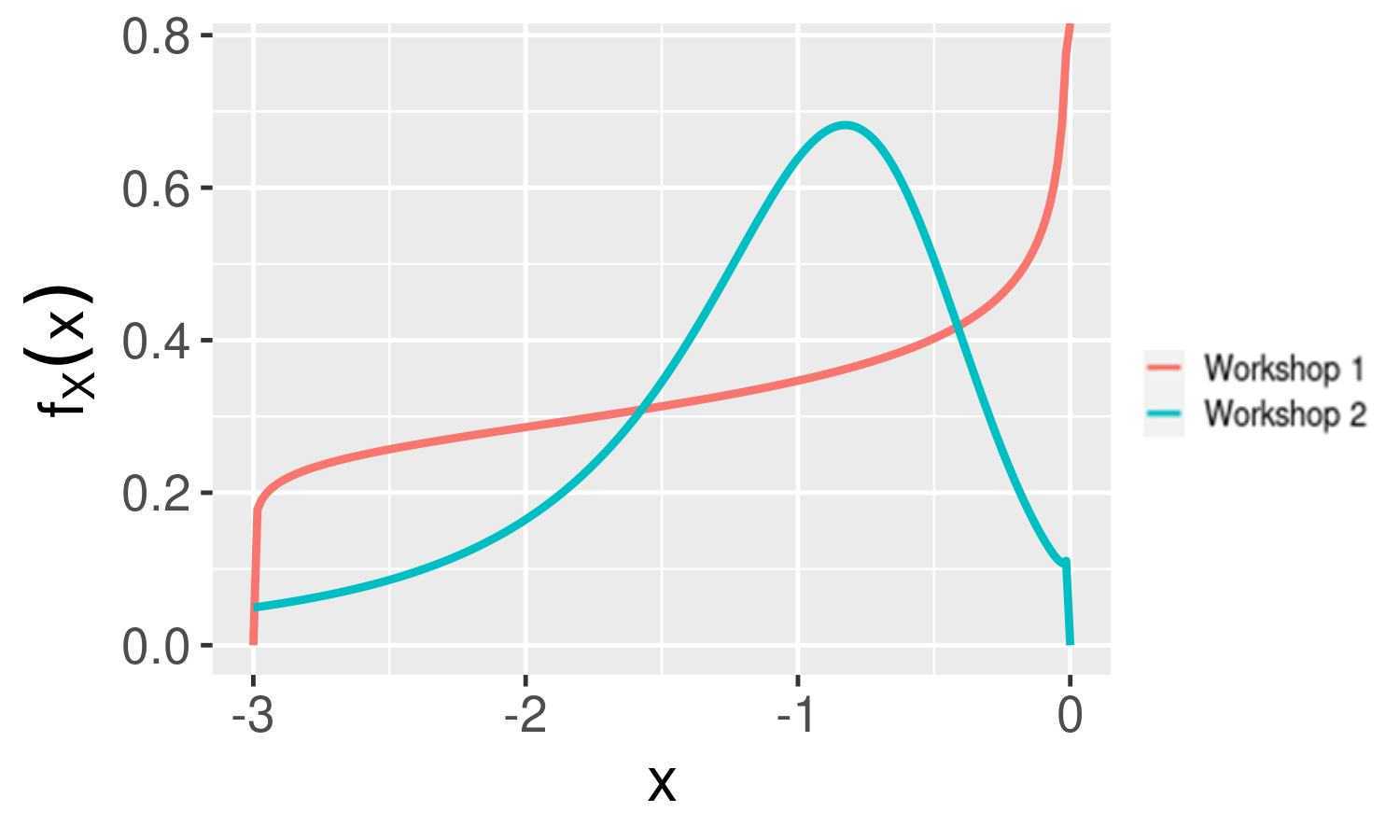


Figure S6: The distributions of 24-month difference in weight between treatment arms elicited in workshop 1 and workshop 2.

##### Consensus Distribution

The lower and upper quartiles remained as this was in agreement. The views of workshop 2 pulled the quartiles towards the median to produce a slightly narrower interquartile range compared with workshop 1. The median was also changed from -1.2 to -1. The revised consensus values are reported in Table S52 and the best fitting distribution is a mirror log normal (Figure S7).

Table S52: Final elicitation values for consensus distribution following workshop 1 and workshop 2 combining views from all experts

| Workshop | L | 0.25 | 0.5 | 0.75 | U | Distribution |
| --- | --- | --- | --- | --- | --- | --- |
| Proposed consensus distribution | -3 | -1.75 | -1 | -0.75 | 0 | Mirror Log-normal |


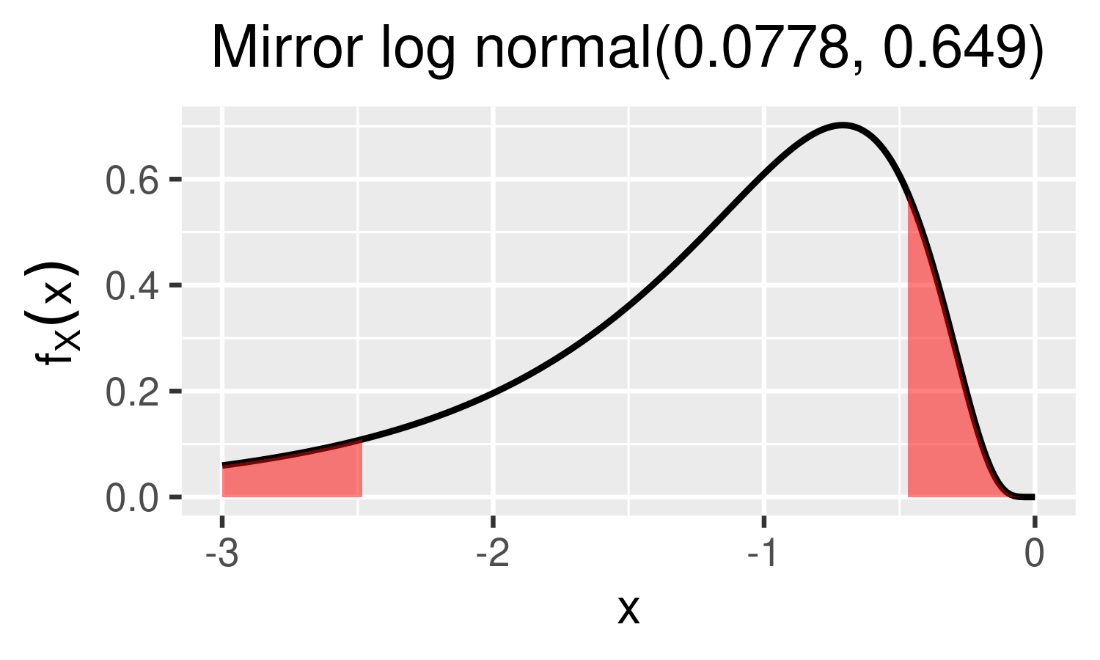


Figure S7: Proposed consensus distribution following workshop 1 and 2 combining all views from experts

Sampling 1000 draws from the consensus distribution produces a median effect of -1.08, mean effect size of -1.33 and standard deviation 0.99, assuming that weight at 12 months is sampled at the median.

## Estimating change in HbA1c conditional on chang in weight

Data from the Weight loss Referral for Adults in Primary care (WRAP) and Glucose Lowering through Weight management (GLoW) trials were pooled to create the sample population. The relationship between a change in BMI and a change in HbA_1c_ (mmol/mol), total cholesterol (mmol/L) or SBP (mmHg) was analysed with a linear mixed-effects regression. The sample population was divided into three subgroups: those with normoglycemia, those with hyperglycaemia and those with T2D. Participants were defined as not having T2D with normoglycemia (NDN) if their HbA1c at baseline was below 42 mmol/mol and they were recorded as not having T2D. Participants were defined as having T2D if they were recorded as being diagnosed with T2D or had an HbA1c of 48 mmol/mol or higher at baseline. Any individual not recorded as being diagnosed with T2D with an HbA1c between 42 mmol/mol and 48 mmol/mol at baseline were assigned to the non-diabetic hyperglycaemia (NDH) group. Table S53 reports the association between change in BMI and HbA1c (%) used to predict trial outcomes for HbA1c conditional on weight loss maintenance.

Table S53: Estimated Change in HbA1c per 1kg/m^2^ change in BMI

|  |  | Mean change in HBa1c (%) | Standard error |
| --- | --- | --- | --- |
| non-diabetic hyperglycaemia | Per 1kg/m^2^ change in BMI | 0.05 | 0.009 |
|  | Weight loss | -0.017 | 0.028 |
|  | Weight loss * 1kg/m2 change in BMI | -0.031 | 0.011 |
| Non-diabetic hyperglycaemia | Per 1kg/m^2^ change in BMI | 0.055 | 0.011 |
| Type 2 diabetes | Per 1kg/m^2^ change in BMI | 0.138 | 0.018 |

## Duration of Intervention Effect

Beyond 24 months we assumed that some reduction in weight due to SWIM is maintained for eight years, after which patients continue on their natural history weight trajectory. Over the 8 years the benefits simulated at 24 months linearly decline.

# Intervention Costs

The micro-costing for the SWiM intervention is reported in Table S53.

Table S54: Unit costs for SWiM intervention

|  | Units | Unit cost | Source |
| --- | --- | --- | --- |
| Scalable costs  Staff time in minutes: Automated email | 150 | £0.43 | University of Cambridge Salary scales |
| Website maintenance | 1 | £7,113.75 | Estimated from trial costs |
| Total scalable costs | 10,000 | £7,178.41 | Assumed population of 10,000 receiving SWiM |
| Phone calls | 7 | £31.50 | https://nationalcareers.service.gov.uk/job-profiles/health-trainer#CareerPathAndProgression |
| Per patient cost |  | £221.22 |  |

# Probabilistic Sensitivity Analysis

Probabilistic sensitivity analysis (PSA) was enabled in the model to describe the uncertainty in parameter inputs of the model and how this translates into uncertainty in the outcomes of the model. A suitable distribution was selected for each parameter, based upon its mean and standard error. Random sampling simultaneously across all input parameter distributions allowed parameter uncertainty to be quantified. 3000 different random samples of parameter values were selected, and each was applied to a simulated cohort of 50,000 individuals meeting the eligibility criteria. These values were chosen based on model stability of the average expected net benefit (Figure S8 and Figure S9). For each PSA sample, the model was run and results compiled. Given the large number of parameters in the model and thus the capacity for error, a thorough process of checking that mean sampling values corresponded to mean parameter values was undertaken to ensure that the results were as accurate as possible.


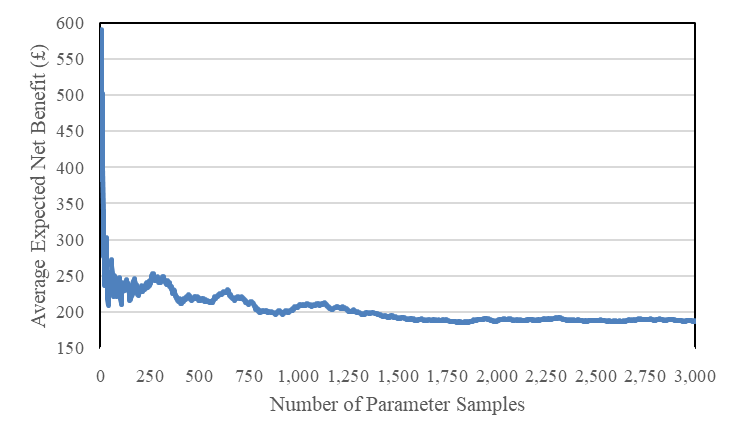


Figure S8: Average Expected Net Benefit by number of parameter samples


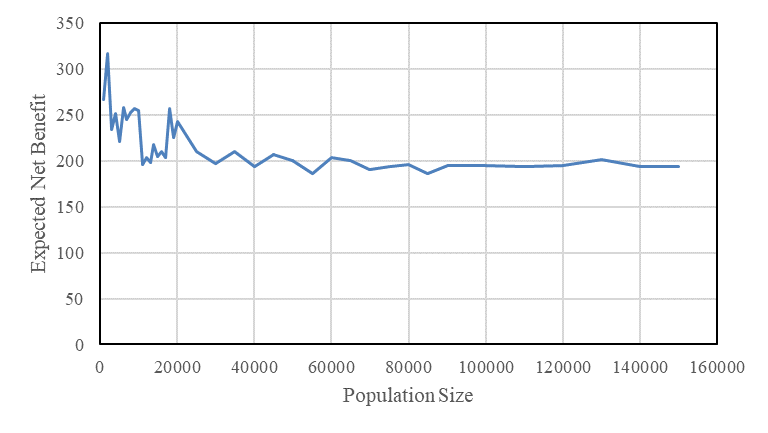


Figure S9: Expected Net Benefit by population size

# Model Validation

The SPHR model has undergone a thorough process of error checking and internal and external validations. Validation of the model to predict metabolic data, diabetes and cardiovascular disease have been reported elsewhere. Verification of the Cumulative incidence of major health outcomes were aligned with expected health outcomes for in this population.


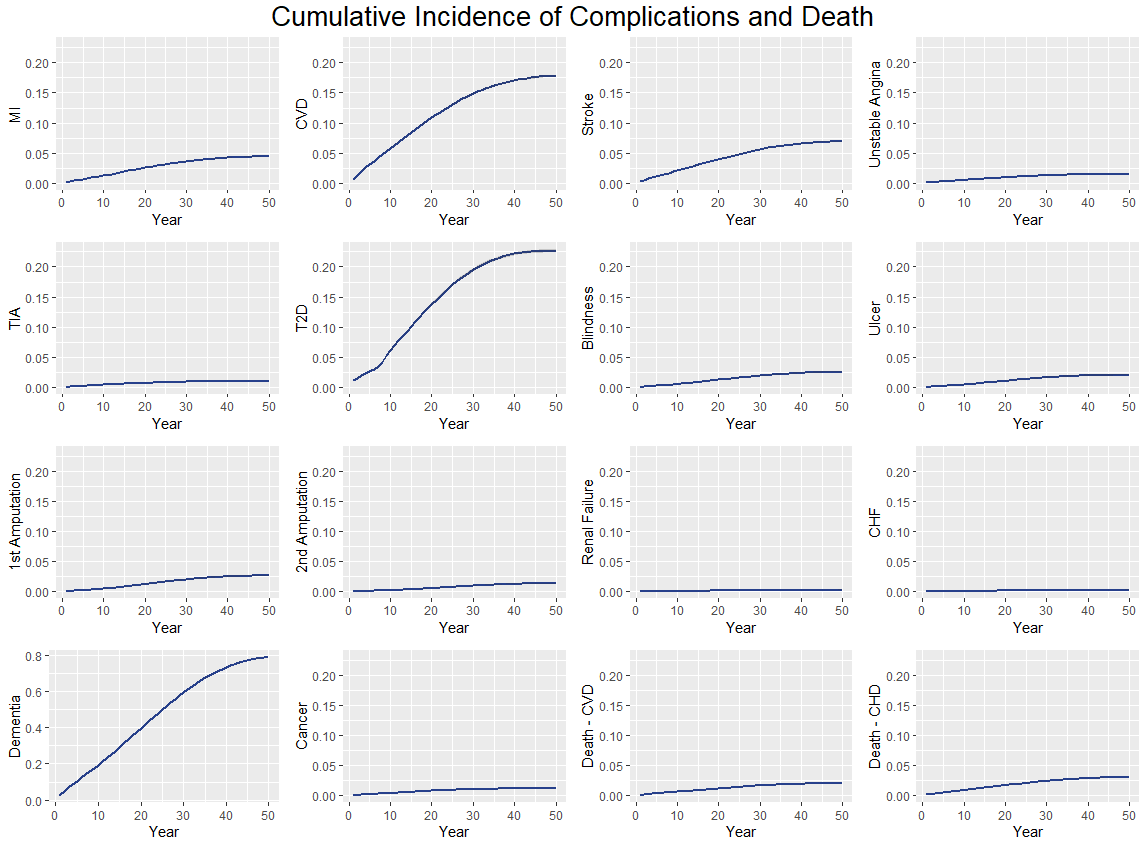


For this analysis we conducted a series of validation tests to compare the simulated outcomes with the 5 year WRAP follow-up data. Given that the unadjusted survival function for the QRISK2 algorithm was calibrated to the 5-year trial data, we included an additional validation test to compare the cardiovascular and diabetes incidence rates to data from the Whitehall II cohort (84).

Table S55: Validation statistics for diabetes and cardiovascular disease incidence

|  |  | Observed | Time-period | Simulated | 95%CI lower | 95% CI upper |  |
| --- | --- | --- | --- | --- | --- | --- | --- |
| Diabetes incidence per 1000 person years | All | 13.149 | 2 years | 12.833 | 4.029 | 24.990 |  |
| Cardiovascular disease incidence per 1000 person years | All | 8.951 | 5 years | 10.223 | 5.701 | 18.711 |  |
|  | Men | 14.988 | 5 years | 16.206 | 8.853 | 27.903 |  |
|  | Women | 5.897 | 5 years | 8.365 | 3.495 | 25.442 |  |
| Diabetes incidence per 1000 person years in Whitehall II | | 18.10* | 17 years | 14.110 | 4.029 | 24.990 |  |
| Cardiovascular disease incidence per 1000 person years in Whitehall II | | 12.728* | 17 years | 12.013 | 6.682 | 20.309 |  |
| * Data obtained for overweight and obese subgroup at baseline with a median follow-up of 17.5 years | | | | | | | |

# Expected Value of Sample Information data

The input data used to estimate the research costs associated with each trial design are summarised in Table S55-Table S58.

Table S56: Research costs for a trial of 500 participants with 12 months follow-up

|  | Staff grade Salary | Salary | Full Time Equivalent/number | Years |
| --- | --- | --- | --- | --- |
| Staff costs | Principle Investigator | £130,000 | 0.2 | 4 |
|  | Senior Invesitgator | £130,000 | 0.05 | 4 |
|  | Project advisor | £70,000 | 0.05 | 4 |
|  | Prog Man | £70,000 | 0.6 | 4 |
|  | Prog Admin | £30,000 | 0.6 | 4 |
|  | Study Coordinator | £60,000 | 1 | 3.5 |
|  | Study Coord Assistant | £30,000 | 0.5 | 4 |
|  | Research Associate | £60,000 | 1 | 4 |
|  | Research Associate | £60,000 | 0 | 1 |
|  | Research Associate | £60,000 | 0 | 4 |
| Consumables | Travel, qualitative interviews, web hosting, other research costs | £200,000 | 1 | 1 |
| Participant costs | Year 1 including honoraria, measurements and samples, consumables. | £103 | 500 | 1 |
|  |  |  | Total | £1,543,500 |

Table S57: Research costs for a trial of 2000 participants with 12 months follow-up

|  | Staff grade Salary | Salary | Full Time Equivalent/number | Years |
| --- | --- | --- | --- | --- |
| Staff costs | Principle Investigator | £130,000 | 0.2 | 4 |
|  | Senior Invesitgator | £130,000 | 0.05 | 4 |
|  | Project advisor | £70,000 | 0.05 | 4 |
|  | Prog Man | £70,000 | 0.6 | 4 |
|  | Prog Admin | £30,000 | 0.6 | 4 |
|  | Study Coordinator | £60,000 | 1 | 3.5 |
|  | Study Coord Assistant | £30,000 | 0.5 | 4 |
|  | Research Associate | £60,000 | 1 | 4 |
|  | Research Associate | £60,000 | 0 | 1 |
|  | Research Associate | £60,000 | 0 | 4 |
| Consumables | Travel, qualitative interviews, web hosting, other research costs | £200,000 | 1 | 1 |
| Participant costs | Year 1 including honoraria, measurements and samples, consumables. | £103 | 2000 | 1 |
|  |  |  | Total | £2,010,996 |

Table S58: Research costs for a trial of 500 participants with 24 months follow-up

|  | Staff grade Salary | Salary | Full Time Equivalent/number | Years |
| --- | --- | --- | --- | --- |
| Staff costs | Principle Investigator | £130,000 | 0.2 | 5 |
|  | Senior Invesitgator | £130,000 | 0.05 | 5 |
|  | Project advisor | £70,000 | 0.05 | 5 |
|  | Prog Man | £70,000 | 0.6 | 5 |
|  | Prog Admin | £30,000 | 0.6 | 5 |
|  | Study Coordinator | £60,000 | 1 | 5.5 |
|  | Study Coord Assistant | £30,000 | 0.5 | 5 |
|  | Research Associate | £60,000 | 1 | 5 |
|  | Research Associate | £60,000 | 0 | 3 |
|  | Research Associate | £60,000 | 0 | 5 |
| Consumables | Travel, qualitative interviews, web hosting, other research costs | £200,000 | N/A | N/A |
| Participant costs | Year 1 including honoraria, measurements and samples, consumables. | £103 | 500 | N/A |
| Participant costs | Year 2 costs measurements and samples | £10 | 500 | N/A |
|  |  |  | Total | £1,999,167 |

Table S59: Research costs for a trial of 2000 participants with 24 months follow-up

|  | Staff grade Salary | Salary | Full Time Equivalent/number | Years |
| --- | --- | --- | --- | --- |
| Staff costs | Principle Investigator | £130,000 | 0.2 | 5 |
|  | Senior Invesitgator | £130,000 | 0.05 | 5 |
|  | Project advisor | £70,000 | 0.05 | 5 |
|  | Prog Man | £70,000 | 0.6 | 5 |
|  | Prog Admin | £30,000 | 0.6 | 5 |
|  | Study Coordinator | £60,000 | 1 | 5.5 |
|  | Study Coord Assistant | £30,000 | 0.5 | 5 |
|  | Research Associate | £60,000 | 1 | 5 |
|  | Research Associate | £60,000 | 0 | 3 |
|  | Research Associate | £60,000 | 0 | 5 |
| Consumables | Travel, qualitative interviews, web hosting, other research costs | £200,000 | N/A | N/A |
| Participant costs | Year 1 including honoraria, measurements and samples, consumables. | £103 | 2000 | N/A |
| Participant costs | Year 2 | £10 | 2000 | N/A |
|  |  |  | Total | £2,568,667 |

**Incidence of eligible populations**

To evaluate the total estimated value of information for a trial based on different potential eligible populations, 6 subgroups were sampled based on individuals leaving a Tier 2 weight management program, a Tier 3 wight management program, a digital Tier 2 weight management, diabetes prevention program and diabetes path to remission program and a final population made up of an even portion of individuals leaving each of these pathways. These subgroups formed potential populations eligible for the weight maintenance program. We estimated the number of individuals that would be eligible for the weight maintenance program after completing each pathway. For the purpose of the estimate a completer was defined as someone who was partially or fully engaged with the programme. Therefore, participants who attended one session and withdrew were not defined as completers and competition was not conditional on outcome.

A number of sources were utilized for our estimates, where possible these were based on government reports. To improve the accuracy of these experts they were reviewed in consultation with an expert in diabetes services commissioning. This consultation highlighted that recent expansion and limited reporting of some of these services may have resulted in out of date or inaccurate estimates. The estimated number of eligible individuals provided during consultation with experts for Digital Tier 2 weight management, Diabetes prevention program and Path to Remission program, with the estimates for Tier 2 and Tier 3 pathways found using reported data, were used during the EVSI analysis. The estimated eligible populations size, sources and assumptions made are reported in Table S55 along with the simulated weight loss.

Table S60: Estimates for the incidence of eligible populations by weight management pathway.

| Weight Management Pathway | Assumptions | Number Referred | Completion Rate | Estimated  Number Completed | Comments | Simulated average weight loss in kg  Mean (SD) |
| --- | --- | --- | --- | --- | --- | --- |
| Tier 2 | April 2021 and March 2022 funded by existing public health grant funding (proportion funded by existing 66%). | 92,045* 0.66 = 61,189 (8) | 24% | 14,901 (8) | Compared to obesity audit referrals 56,260 and using the completion rate from source data of 24% to get annual completion of 13,700. | -4.91 (1.88) |
| Tier 3 | Tier 3 referrals January to March 2023 multiplied by 4. Completion rate based on proportion completing phase 2 of weight management program. | 15560 (85) | 35% | 5,466 (86) | Alternative completion rates found were 40% (87) or 50% (9), however, these studies had small sample sizes. | -6.23 (1.67) |
| Diabetes prevention program | Total referrals between December 2017 to December 2018. | 233,899 (88) | 19% | 44,441 (88) | Revised estimate received from expert consultation 53,000 | -4.68 (1.71) |
| Digital Tier 2 | Tier 2 services delivered digitally between April 2021 to March 2022. | 31,861 (89) | 45% | 14,268 (89) |  | -5.61 (1.91) |
| Diabetes Path to Remission. | A contract award allowing provision of up to 10,500 interventions annually. | 7540 (90) | 13% | 960 (90)  (estimate 1174 per 12 months for 42 ICBs) | Data from 10 out of 42 ICBs over 16 months (01/09/2020-31/12/2022), and 21 ICBs over 12 months (01/01/2022-31/12/2022). Population estimate based on future referrals for 42 ICBs | -3.86 (1.77) |
| All | Sum of all subgroups minus the overlap between Tier 2 and digital Tier 2, 1619 (8). |  |  | 73,794 |  | -4.95 (1.91) |

# References-

1. Squires H, Chilcott J, Akehurst R, Burr J, Kelly MP. A Framework for Developing the Structure of Public Health Economic Models. Value Health. 2016;19(5):588-601.

2. National Institute for Health and Care Excellence. PH35: Preventing type 2 diabetes: population and community-level interventions. National Institute for Health and Care Excellence [Internet]. 2011; NICE public health guidance 35. Available from: <https://www.nice.org.uk/guidance/ph35>.

3. National Institute for Health and Care Excellence. PH38 Preventing type 2 diabetes - risk identification and interventions for individuals at high risk: guidance. National Institute for Health and Care Excellence [Internet]. 2012; NICE public health guidance 38. Available from: <http://guidance.nice.org.uk/PH38/Guidance/pdf/English>.

4. NatCen Social Research UCL, Department of Epidemiology and Public Health. Health Survey for England, 2018.: UK Data Service; 2022.

5. Mortality Statistics: Deaths registered in England and Wales (Series DR), 2014. Office of National Statistics [Internet]. 2017. Available from: <http://webarchive.nationalarchives.gov.uk/20160105160709/http://www.ons.gov.uk/ons/publications/re-reference-tables.html?edition=tcm%3A77-378961>.

6. Breeze P, Squires H, Chilcott J, Stride C, Diggle PJ, Brunner E, et al. A statistical model to describe longitudinal and correlated metabolic risk factors: the Whitehall II prospective study. J Public Health (Oxf). 2015.

7. Lovelace R, Dumont M. Spatial microsimulation with R: Chapman and Hall/CRC; 2017.

8. Office for Health Improvement and Disparities (OHID) Adult tier 2 weight management services provisional data for April 2021 to December 2022 (experimental statistics). 2023.

9. Jennings A HC, Kumaravel B, Bachmann MO, Steel N, Capehorn M, Cheema K. Evaluation of a multidisciplinary T ier 3 weight management service for adults with morbid obesity, or obesity and comorbidities, based in primary care. . Clinical obesity. 2014;4(5):254-66.

10. Howarth E BP, Kontopantelis E, Soiland-Reyes C, Meacock R, Whittaker W, Cotterill S. ‘Going the distance’: an independent cohort study of engagement and dropout among the first 100 000 referrals into a large-scale diabetes prevention program. . BMJ Open Diabetes Research and Care 2020;8(2):e001835.

11. Ahern AL WJ, Wells E, Sharp SJ, Islam N, Lawlor ER, Duschinsky R, Hill AJ, Doble B, Wilson E, Morris S. . Clinical and cost-effectiveness of a diabetes education and behavioural weight management programme versus a diabetes education programme in adults with a recent diagnosis of type 2 diabetes: Study protocol for the Glucose Lowering through Weight management (GLoW) randomised controlled trial. BMJ open 2020;10(4):e035020.

12. Ward S, Lloyd JM, Pandor A, Holmes M, Ara R, Ryan A, et al. A systematic review and economic evaluation of statins for the prevention of coronary events. Health Technol Assess. 2007;11(14):1-iv.

13. Rabe-Hesketh S, Skrondal A. Multilevel and Longitudinal Modelling Using Stata. Second edition ed. College Station: StataCorp; 2008 2008.

14. Arbeev KG, Ukraintseva SV, Akushevich I, Kulminski AM, Arbeeva LS, Akushevich L, et al. Age trajectories of physiological indices in relation to healthy life course. Mech Ageing Dev. 2011;132(3):93-102.

15. Young KG, McGovern AP, Barroso I, Hattersley AT, Jones AG, Shields BM, et al. The impact of population-level HbA1c screening on reducing diabetes diagnostic delay in middle-aged adults: a UK Biobank analysis. Diabetologia. 2022:1-10.

16. Clarke PM, Gray AM, Briggs A, Farmer AJ, Fenn P, Stevens RJ, et al. A model to estimate the lifetime health outcomes of patients with type 2 diabetes: the United Kingdom Prospective Diabetes Study (UKPDS) Outcomes Model (UKPDS no. 68). Diabetologia. 2004;47(10):1747-59.

17. Ara R, Pandor A, Stevens J, Rees A, Rafia R. Early high-dose lipid-lowering therapy to avoid cardiac events: a systematic review and economic evaluation. Health Technol Assess. 2009;13(34):1-118.

18. National Institute for Health and Care Excellence. Hypertension: Clinical management of primary hypertension in adults. 2011 2011. Report No.: CG 127.

19. Wald DS, Law M, Morris JK, Bestwick JP, Wald NJ. Combination therapy versus monotherapy in reducing blood pressure: meta-analysis on 11,000 participants from 42 trials. Am J Med. 2009;122(3):290-300.

20. National Institute of Health and Care Excellence. Statins for the prevention of cardiovascular events in patients at increased risk of developing cardiovascular disease or those with established cardiovascular disease. 2006 2006. Report No.: Technology appraisals, TA94.

21. Hippisley-Cox J, Coupland C. Development and validation of QDiabetes-2018 risk prediction algorithm to estimate future risk of type 2 diabetes: cohort study. BMJ. 2017;359:j5019.

22. Watson P, Preston L, Squires H, Chilcott J, Brennan A. Modelling the Economics of Type 2 Diabetes Mellitus Prevention: A Literature Review of Methods. Appl Health Econ Health Policy. 2014;12(3):239-53.

23. Hayes AJ, Leal J, Gray AM, Holman RR, Clarke PM. UKPDS outcomes model 2: a new version of a model to simulate lifetime health outcomes of patients with type 2 diabetes mellitus using data from the 30 year United Kingdom Prospective Diabetes Study: UKPDS 82. Diabetologia. 2013;56(9):1925-33.

24. D'Agostino RB, Sr., Vasan RS, Pencina MJ, Wolf PA, Cobain M, Massaro JM, et al. General cardiovascular risk profile for use in primary care: the Framingham Heart Study. Circulation. 2008;117(6):743-53.

25. Hippisley-Cox J, Coupland C, Vinogradova Y, Robson J, Minhas R, Sheikh A, et al. Predicting cardiovascular risk in England and Wales: prospective derivation and validation of QRISK2. BMJ. 2008;336(7659):1475-82.

26. McEwan P, Bennett H, Ward T, Bergenheim K. Refitting of the UKPDS 68 risk equations to contemporary routine clinical practice data in the UK. Pharmacoeconomics. 2015;33(2):149-61.

27. ClinRisk. QResearch [Internet]. 2019. Available from: <http://www.qrisk.org/>.

28. Hippisley-Cox J, Coupland C, Robson J, Brindle P. Derivation, validation, and evaluation of a new QRISK model to estimate lifetime risk of cardiovascular disease: cohort study using QResearch database. BMJ. 2010;341:c6624. doi: 10.1136/bmj.c6624.:c6624.

29. Khaw KT, Wareham N, Luben R, Bingham S, Oakes S, Welch A, et al. Glycated haemoglobin, diabetes, and mortality in men in Norfolk cohort of european prospective investigation of cancer and nutrition (EPIC-Norfolk). BMJ. 2001;322(7277):15-8.

30. Kannel WB, D'Agostino RB, Silbershatz H, Belanger AJ, Wilson PW, Levy D. Profile for estimating risk of heart failure. Arch Intern Med. 1999;159(11):1197-204.

31. Kaffashian S, Dugravot A, Brunner EJ, Sabia S, Ankri J, Kivimaki M, et al. Midlife stroke risk and cognitive decline: a 10-year follow-up of the Whitehall II cohort study. Alzheimers Dement. 2013;9(5):572-9.

32. Johansen NB, Vistisen D, Brunner EJ, Tabak AG, Shipley MJ, Wilkinson IB, et al. Determinants of aortic stiffness: 16-year follow-up of the Whitehall II study. PLoS One. 2012;7(5):e37165.

33. Dadvand P, Rankin J, Shirley MD, Rushton S, Pless-Mulloli T. Descriptive epidemiology of congenital heart disease in Northern England. Paediatr Perinat Epidemiol. 2009;23(1):58-65.

34. Davies M, Hobbs F, Davis R, Kenkre J, Roalfe AK, Hare R, et al. Prevalence of left-ventricular systolic dysfunction and heart failure in the Echocardiographic Heart of England Screening study: a population based study. Lancet. 2001;358(9280):439-44.

35. Lahmann PH, Hoffmann K, Allen N, van Gils CH, Khaw KT, Tehard B, et al. Body size and breast cancer risk: findings from the European Prospective Investigation into Cancer And Nutrition (EPIC). Int J Cancer. 2004;111(5):762-71.

36. Pischon T, Lahmann PH, Boeing H, Friedenreich C, Norat T, Tjonneland A, et al. Body size and risk of colon and rectal cancer in the European Prospective Investigation Into Cancer and Nutrition (EPIC). J Natl Cancer Inst. 2006;98(13):920-31.

37. Renehan AG, Tyson M, Egger M, Heller RF, Zwahlen M. Body-mass index and incidence of cancer: a systematic review and meta-analysis of prospective observational studies. Lancet. 2008;371(9612):569-78.

38. Schett G, Kleyer A, Perricone C, Sahinbegovic E, Iagnocco A, Zwerina J, et al. Diabetes is an independent predictor for severe osteoarthritis: results from a longitudinal cohort study. Diabetes Care. 2013;36(2):403-9.

39. Palmer AJ, Roze S, Valentine WJ, Minshall ME, Foos V, Lurati FM, et al. The CORE Diabetes Model: Projecting long-term clinical outcomes, costs and cost-effectiveness of interventions in diabetes mellitus (types 1 and 2) to support clinical and reimbursement decision-making. Curr Med Res Opin. 2004;20(Suppl. 1):S5-S26.

40. Golden SH, Lazo M, Carnethon M, Bertoni AG, Schreiner PJ, Diez Roux AV, et al. Examining a bidirectional association between depressive symptoms and diabetes. JAMA. 2008;299(23):2751-9.

41. Whyte EM, Mulsant BH, Vanderbilt J, Dodge HH, Ganguli M. Depression after stroke: a prospective epidemiological study. J Am Geriatr Soc. 2004;52(5):774-8.

42. Walters K, Hardoon S, Petersen I, Iliffe S, Omar RZ, Nazareth I, et al. Predicting dementia risk in primary care: development and validation of the Dementia Risk Score using routinely collected data. Bmc Medicine. 2016;14.

43. Briggs A, Claxton K, Sculfer M. Decision Modelling for Health Economic Evaluation2006 2006.

44. Wimo A, Religa D, Spangberg K, Edlund AK, Winblad B, Eriksdotter M. Costs of diagnosing dementia: results from SveDem, the Swedish Dementia Registry. Int J Geriatr Psychiatry. 2013;28(10):1039-44.

45. Xie J, Brayne C, Matthews FE. Survival times in people with dementia: analysis from population based cohort study with 14 year follow-up. BMJ. 2008;336(7638):258-62.

46. Bond M, Rogers G, Peters J, Anderson R, Hoyle M, Miners A, et al. The effectiveness and cost-effectiveness of donepezil, galantamine, rivastigmine and memantine for the treatment of Alzheimer's disease (review of Technology Appraisal No. 111): a systematic review and economic model. Health Technol Assess. 2012;16(21):1-470.

47. Prince M, Knapp M, Guerchet M, McCrone P, Prina P, Comas-Herrera A, et al. Dementia UK Update. Cognitive Function and Ageing Study [Internet]. 2014. Available from: <http://www.cfas.ac.uk/files/2015/07/P326_AS_Dementia_Report_WEB2.pdf>.

48. Getsios D, Blume S, Ishak KJ, Maclaine GD. Cost effectiveness of donepezil in the treatment of mild to moderate Alzheimer's disease: a UK evaluation using discrete-event simulation. Pharmacoeconomics. 2010;28(5):411-27.

49. Cancer Survival in England: Patients Diagnosed, 2006–2010 and Followed up to 2011. Office of National Statistics [Internet]. 2012. Available from: <http://www.ons.gov.uk/ons/publications/re-reference-tables.html?edition=tcm%3A77-277733>.

50. Seshasai SR, Kaptoge S, Thompson A, Di AE, Gao P, Sarwar N, et al. Diabetes mellitus, fasting glucose, and risk of cause-specific death. N Engl J Med. 2011;364(9):829-41.

51. James BD, Leurgans SE, Hebert LE, Scherr PA, Yaffe K, Bennett DA. Contribution of Alzheimer disease to mortality in the United States. Neurology. 2014;82(12):1045-50.

52. Jones KC BA. Unit costs of health and social care. PSSRU; 2021 2021.

53. National Cost Collection - National Schedule of NHS Costs 2020/21. In: Digital N, editor. NHS Digital2022.

54. British National Formulary. <https://bnfniceorguk/> [Internet]. 2022.

55. CG127 Hypertension: costing template. National Institute for Care and Clinical Excellence [Internet]. 2011. Available from: <http://guidance.nice.org.uk/CG127/CostingTemplate/xls/English>.

56. Gillett M, Royle P, Snaith A, Scotland G, Poobalan A, Imamura M, et al. Non-pharmacological interventions to reduce the risk of diabetes in people with impaired glucose regulation: a systematic review and economic evaluation. Health Technol Assess. 2012;16(33):1-iv.

57. Belsey JD, Pittard JB, Rao S, Urdahl H, Jameson K, Dixon T. Self blood glucose monitoring in type 2 diabetes. A financial impact analysis based on UK primary care. Int J Clin Pract. 2009;63(3):439-48.

58. Poole C, Tetlow T, McEwan P, Holmes P, Currie C. The prescription cost of managing people with type 1 and type 2 diabetes following initiation of treatment with either insulin glargine or insulin determir in routine general practice in the UK: a retrospective database analysis. Current Medical Research and Opinion. 2007;23(1):S41-S8.

59. National Institute for Health and Care Excellence. Hypertension in adults: diagnosis and management. Cost effectiveness analysis: Treatment initiation threshold for people with stage 1 hypertension.; 2019.

60. Luengo-Fernandez R, Gray AM, Rothwell PM. A population-based study of hospital care costs during 5 years after transient ischemic attack and stroke. Stroke. 2012;43(12):3343-51.

61. Clarke P, Gray A, Legood R, Briggs A, Holman R. The impact of diabetes-related complications on healthcare costs: results from the United Kingdom Prospective Diabetes Study (UKPDS Study No. 65). Diabet Med. 2003;20(6):442-50.

62. Danese MD GM, Kutikova L, Griffiths RI, Azough A, Khunti K, Seshasai SR, Ray KK. Estimating the economic burden of cardiovascular events in patients receiving lipid-modifying therapy in the UK. . BMJ open 2016;6(8):e011805.

63. SSNAP NGC. Sentinel Stroke National Audit Programme: Cost and Cost-effectiveness analysis (Technical Report)2016. Available from: <https://www.strokeaudit.org/SupportFiles/Documents/Health-economics/Health-economic-report-2016.aspx>.

64. Alva ML, Gray, A., Mihaylova, B., Leal, J. and Holman, R.R. The impact of diabetes‐related complications on healthcare costs: new results from the UKPDS (UKPDS 84). Diabetic medicine. 2015;32(4):459-66.

65. Walker S AM, Manca A, Palmer S, Gale CP, Shah AD, Abrams KR, Crowther M, Timmis A, Hemingway H, Sculpher M. . Long-term healthcare use and costs in patients with stable coronary artery disease: a population-based cohort using linked health records (CALIBER). . European Heart Journal–Quality of Care and Clinical Outcomes. 2016;2(2):125-40.

66. **National Cost Collection - National Schedule of NHS Costs 2021/22**

In: Digital N, editor. 2022.

67. National Institute for Health and Care Excellence. NG28 Health Economic Model Report2015 18.10.2023. Available from: <https://www.nice.org.uk/guidance/ng28/evidence/health-economic-model-report-pdf-10959500845>.

68. Kerr M BE, Chadwick P, Evans T, Kong WM, Rayman G, Sutton‐Smith M, Todd G, Young B, Jeffcoate WJ. The cost of diabetic foot ulcers and amputations to the National Health Service in England. Diabetic Medicine. 2019;36(8):995-1002.

69. Laudicella M WB, Burns E, Smith PC. Cost of care for cancer patients in England: evidence from population-based patient-level data. . British journal of cancer 2016;114(11):1286-92.

70. The economic costs of arthritis for the UK economy. Oxford Economics [Internet]. 2014. Available from: <https://www.oxfordeconomics.com/publication/open/222531>.

71. Chalder M, Wiles NJ, Campbell J, Hollinghurst SP, Searle A, Haase AM, et al. A pragmatic randomised controlled trial to evaluate the cost-effectiveness of a physical activity intervention as a treatment for depression: the treating depression with physical activity (TREAD) trial. Health Technol Assess. 2012;16(10):1-iv.

72. Breeze P, Gray LA, Thomas C, Bates SE, Brennan A. Estimating the impact of changes in weight and BMI on EQ-5D-3L: a longitudinal analysis of a behavioural group-based weight loss intervention. Quality of Life Research. 2022;31(11):3283-92.

73. Ara R, Wailoo A. NICE DSU Technical Support Document 12: The use of health state utility values in decision models. 2011 2011.

74. Alva M, Gray A, Mihaylova B, Clarke P. The Effect of Diabetes Complications on Health-Related Quality of Life: The importance of longitudinal data to address patient heterogeneity. Health economics. 2014;23(4):487-500.

75. Coffey JT, Brandle M, Zhou H, Marriott D, Burke R, Tabaei BP, et al. Valuing health-related quality of life in diabetes. Diabetes Care. 2002;25(12):2238-43.

76. Lung TW, Hayes AJ, Hayen A, Farmer A, Clarke PM. A meta-analysis of health state valuations for people with diabetes: explaining the variation across methods and implications for economic evaluation. Qual Life Res. 2011;20(10):1669-78.

77. Peasgood T, Brazier J. Is Meta-Analysis for Utility Values Appropriate Given the Potential Impact Different Elicitation Methods Have on Values? Pharmacoeconomics. 2015;33(11):1101-5.

78. Yabroff KR, Lawrence WF, Clauser S, Davis WW, Brown ML. Burden of illness in cancer survivors: findings from a population-based national sample. J Natl Cancer Inst. 2004;96(17):1322-30.

79. Black C, Clar C, Henderson R, MacEachern C, McNamee P, Quayyum Z, et al. The clinical effectiveness of glucosamine and chondroitin supplements in slowing or arresting progression of osteoarthritis of the knee: a systematic review and economic evaluation. Health Technol Assess. 2009;13(52):1-148.

80. Zimovetz EA, Wolowacz SE, Classi PM, Birt J. Methodologies used in cost-effectiveness models for evaluating treatments in major depressive disorder: a systematic review. Cost Eff Resour Alloc. 2012;10(1):1-10.

81. Benedict A, Arellano J, De CE, Baird J. Economic evaluation of duloxetine versus serotonin selective reuptake inhibitors and venlafaxine XR in treating major depressive disorder in Scotland. J Affect Disord. 2010;120(1-3):94-104.

82. Jonsson L, Andreasen N, Kilander L, Soininen H, Waldemar G, Nygaard H, et al. Patient- and proxy-reported utility in Alzheimer disease using the EuroQoL. Alzheimer Dis Assoc Disord. 2006;20(1):49-55.

83. Shearer J, Green C, Ritchie CW, Zajicek JP. Health state values for use in the economic evaluation of treatments for Alzheimer's disease. Drugs Aging. 2012;29(1):31-43.

84. Hinnouho GM, Czernichow S, Dugravot A, Nabi H, Brunner EJ, Kivimaki M, et al. Metabolically healthy obesity and the risk of cardiovascular disease and type 2 diabetes: the Whitehall II cohort study. Eur Heart J. 2015;36(9):551-9.

85. NHS. National Obesity Audit (England): Weight Management Services, Quarter 1 to Quarter 4 2022/23. Community Services Dataset of Weight Management Services. NHS Digital2023.

86. Logue J AG, Gillies M, Forde L, Morrison DS. Outcomes of a specialist weight management programme in the UK National Health Service: prospective study of 1838 patients. BMJ open 2014;4(1):e003747.

87. Nield L KS. Outcomes of a community‐based weight management programme for morbidly obese populations. Journal of Human Nutrition and Dietetics 2016;29(6):669-76.

88. Valabhji J BE, Bradley D, Bakhai C, Fagg J, O’Neill S, Young B, Wareham N, Khunti K, Jebb S, Smith J. Early outcomes from the English National health service diabetes prevention programme. . Diabetes care 2020;43(1):152-60.

89. Taylor K, Indulkar T, Thompson B, Pinkard C, Barron E, Frost T, et al. Early outcomes of referrals to the English National Health Service Digital Weight Management Programme. Obesity. 2024;32(6):1083-92.

90. Valabhji J, Gorton T, Barron E, Safazadeh S, Earnshaw F, Helm C, et al. Early findings from the NHS Type 2 Diabetes Path to Remission Programme: a prospective evaluation of real-world implementation. The Lancet Diabetes & Endocrinology. 2024.

1. The model did not converge when BMI slope was included as a predictor for HDL growth. [↑](#footnote-ref-1)
